# Enhanced Antiviral Activity of Novel Umifenovir Derivatives against SARS-CoV-2: Insights from an International Collaborative Study

**DOI:** 10.1021/acsomega.5c13080

**Published:** 2026-04-06

**Authors:** Melina Mottin, Christopher D. Jurisch, Sabrina Silva-Mendonça, Donald Seanego, Paulo R. P. da Silva Ramos, Caroline S. Freitas, Mayara Mattos, Natalia Fintelman-Rodrigues, Carolina Q. Sacramento, Florence Guivel-Benhassine, Timothée Bruel, Liezl Krugmann, Ana C. Puhl, Fabio Urbina, Thomas R. Lane, Eric M. Merten, Kenneth H. Pearce, Alexander Lepioshkin, Artem Poromov, Natalia Monakhova, Olivier Schwartz, Kelly Chibale, Thiago M. L. Souza, Sean Ekins, Vadim Makarov, Richard K. Gessner, Carolina H. Andrade

**Affiliations:** 1 Center for the Research and Advancement in Fragments and Molecular Targets (CRAFT), Faculdade de Ciências Farmaceuticas de Ribeirão Preto, Universidade de São Paulo, Ribeirão Preto, SP 05508-060, Brazil; 2 Laboratory for Molecular Modeling and Drug Design (LabMol), Faculdade de Farmácia, 67824 Universidade Federal de Goiás, Goiânia, GO 74605-170, Brazil; 3 Holistic Drug Discovery and Development Centre (H3D), University of Cape Town (UCT), Rondebosch 7701, South Africa; 4 Laboratory of Immunopharmacology, Oswaldo Cruz Institute (IOC), Oswaldo Cruz Foundation (Fiocruz), Rio de Janeiro, RJ 21040-360, Brazil; 5 National Institute for Science and Technology on Innovation in Diseases of Neglected Populations (INCT/IDPN), Center for Technological Development in Health (CDTS), Fiocruz, Rio de Janeiro, RJ 21040-900, Brazil; 6 Virus and Immunity Unit, Institut Pasteur, Université Paris Cité, 28 rue du Dr Roux, Paris Cedex 15 75724, France; 7 Collaborations Pharmaceuticals, Inc., 840 Main Campus Drive, Lab 3510, Raleigh, North Carolina 27606, United States; 8 Center for Integrative Chemical Biology and Drug Discovery, Chemical Biology and Medicinal Chemistry, Eshelman School of Pharmacy, University of North Carolina, Chapel Hill, North Carolina 27599, United States; 9 Research Center of Biotechnology RAS, 33-1 Leninsky prospect, Moscow 119071, Russia; 10 Center for Excellence in Artificial Intelligence (CEIA), Instituto de Informática, 67824 Universidade Federal de Goiás, Goiânia, GO 74605-220, Brazil

## Abstract

Despite the availability of vaccines and treatments for COVID-19, the emergence of new SARS-CoV-2 variants continues to challenge vaccine-induced immunity, emphasizing the need to develop antiviral therapies to combat these variants and other viruses. Umifenovir (UMF), marketed as Arbidol, is a broad-spectrum antiviral drug approved in Russia and China for influenza viruses A and B. Recent studies have suggested its potential against SARS-CoV-2, demonstrating its ability to inhibit viral replication and obstruct viral entry by targeting the spike protein, despite having low oral bioavailability and short half-life. In this work, we conducted an initial antiviral screening that identified a hit compound (a UMF analogue), followed by the rational design, synthesis, and evaluation of novel UMF derivatives against SARS-CoV-2. This process employed generative models and structure–activity relationship studies (SAR), focusing on the UMF binding site on the S2 subunit of the spike protein. The derivatives demonstrated potent antiviral activity, with EC_50_ values ranging from 0.05 to 1.23 μM in S-Fuse assays against the Omicron variant (BA.2.86.1 lineage) and from 1.4 to 1.53 μM in Calu-3 cells, while showing low cytotoxicity and high selectivity. The most promising compound, **11**, predicted by the machine learning-based generative models, exhibited an antiviral potency (EC_50_) of 1.53 μM in Calu-3 cells infected with the B.1. variant, a CC_50_ of 93.9 μM, with a selectivity index of 61.37. Additionally, **11** displayed substantial antiviral activity against various SARS-CoV-2 variants, including against the Omicron variant (BA.2.86.1), with an EC_50_ of 0.73 μM in the S-Fuse assay. Furthermore, **11** demonstrated favorable mouse pharmacokinetic properties, including improved aqueous solubility at physiological pH, a prolonged terminal half-life, and increased systemic exposure. Overall, virucidal assays demonstrated that **11** lacks direct virucidal activity, and viral adsorption assays further showed that this compound does not impair viral attachment. Consistent with these findings, exploratory molecular docking results should be regarded as hypothesis-generating, suggesting a potential involvement of compound **11** in later viral entry events rather than in direct viral inactivation. These findings provide a foundation for advancing **11** as a hit compound for further optimization within antiviral strategies against SARS-CoV-2 variants.

## Graphical abstract

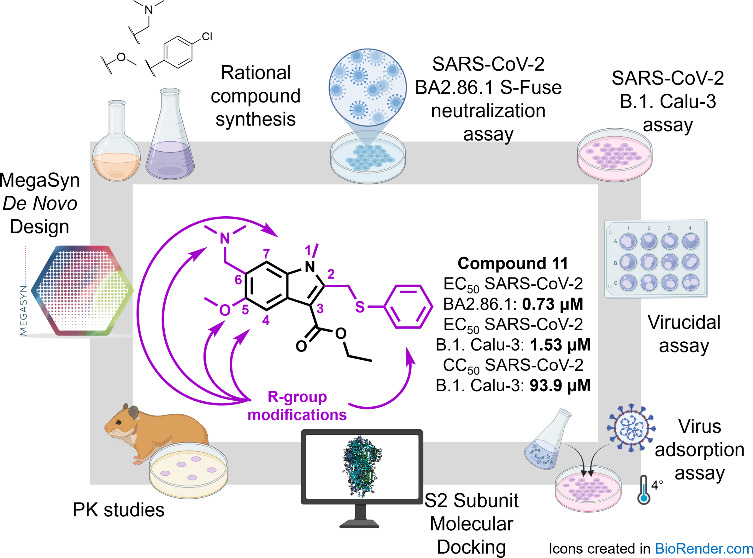

## Introduction

The SARS-CoV-2 pandemic posed significant challenges to global healthcare systems, with its rapid spread resulting in over 7 million^1^ fatalities and severe economic disruptions.^2,3^ This virus, belonging to the Coronaviridae family,^4^ is the third zoonotic coronavirus, following SARS-CoV and MERS-CoV, to cause a major outbreak in humans. Infection with this virus leads to extensive morbidity and mortality, and a broad range of clinical symptoms such as cough, loss of smell and taste, respiratory distress, pneumonia, and extrapulmonary events characterized by a sepsis-like disease collectively called coronavirus disease 2019 (COVID-19).^5^

While the rapid development and deployment of vaccines have significantly reduced the global health burden (and saved countless lives), the inevitable emergence of new variants^6^ threatens the efficacy of host immune responses induced by vaccination. Combined with the virus’s high transmissibility and the diversity of clinical manifestations, ranging from mild symptoms to severe respiratory failure, these variants continue to challenge public health efforts. As a result, the discovery and development of new antiviral agents remain imperative.

The molecular targets most examined for small-molecule inhibitors of SARS-CoV-2 ^7^ include the main protease (M^pro^),^8^ papain-like protease (PL^pro^),^9–11^ RNA-dependent RNA polymerase (RdRp),^12^ spike (S) protein,^13^ Nsp14 exoribonuclease,^14^ and Nsp13 helicase.^15^ These proteins are essential for viral replication, entry, and immune evasion. The spike protein (S) is a surface glycoprotein that plays a crucial role in viral entry by mediating the attachment and fusion within the host cell membrane, making it a key target for antiviral therapies.^16^ Several S protein inhibitors, such as monoclonal antibodies^17–19^ and small molecules,^20^ have shown promise in blocking the interaction between the S protein and the angiotensin-converting enzyme 2 (ACE2) receptor, thereby preventing viral entry. However, discovering effective S protein inhibitors is challenging due to the high mutation rate of this protein, which can lead to the emergence of variants that evade these inhibitors, reducing their efficacy over time.

Umifenovir (UMF), trade name Arbidol, is an indole-derived broad-spectrum antiviral compound first synthesized by Russian chemists in 1990.^21^ It has been licensed in Russia (1993) and China (2006) for the prevention and treatment of human influenza viruses A and B^22^ and has also been used to treat postinfluenza complications. More recently, studies have demonstrated that UMF exhibits *in vitro* activity against other viruses,^23^ including Hepatitis B and C,^24^ Ebola,^25^ and arthropod-borne Zika^26^ and West Nile flaviviruses.^27^ Moreover, UMF has demonstrated *in vitro* antiviral activity against SARS-CoV-2 infections^28–31^ with an EC_50_ value of 4.11 μM and a CC_50_ of 31.79 μM in Vero E6 cells^32^ infected with the nCoV-2019BetaCoV strain and produced therapeutic effects^33–38^ when given to patients infected with COVID-19, either as a monotherapy or combined with other drugs. UMF exhibits activity against SARS-CoV-2 by binding to the S protein on the S2 domain, potentially preventing viral entry into host cells,^39–41^ which highlights the potential of repurposing known antiviral agents to treat COVID-19.^42–44^

Despite these promising laboratory findings and clinical observations, UMF efficacy in treating COVID-19 remains uncertain. Some clinical studies^36^ and meta-analyses^45^ have yielded inconsistent results, showing no significant improvements in key outcomes such as viral clearance, symptom resolution, or overall recovery, particularly in non-intensive care unit patients. A recent meta-analysis^45^ concluded that UMF provided no significant advantage over nonantiviral treatments or other therapeutic agents for COVID-19. To address these gaps and conflicting findings, high-quality randomized controlled clinical trials are needed to definitively evaluate the efficacy and safety of UMF in treating COVID-19.

Additionally, UMF has several limitations that hinder its broader therapeutic potential. It exhibits poor human pharmacokinetics (PK),^46,47^ including low oral bioavailability and a short half-life,^48^ necessitating frequent dosing to maintain therapeutic levels. Additionally, UMF undergoes rapid metabolism,^46^ leading to reduced systemic exposure^46^ and, in some cases, limited efficacy.^46^ Reports of side effects, such as mild gastrointestinal disturbances,^38^ are minimal and generally infrequent, suggesting a favorable safety profile^36^ when administered alone.^29^ These issues highlight the need for structural optimization and development of derivatives with improved PK profiles and reduced side effects.

This work was conducted as part of an international project collaboration involving research laboratories from BRICS countries, Brazil, South Africa, and Russia, along with partners from the United States and France, during and following the COVID-19 pandemic, to find new drug candidates against COVID-19. For that, we screened a diverse chemical library from the Research Center of Biotechnology (RAS) for antiviral activity. Both UMF and its close analogs are effective against multiple SARS-CoV-2 variants. Building on these findings, we collaboratively designed, synthesized, and evaluated novel UMF derivatives with enhanced antiviral efficacy and improved PK properties. Our comprehensive approach included structure–activity relationship (SAR) analysis and generative design.

## Results and Discussion

Figure 1 illustrates the overall workflow, integrating *in silico*, *in vitro,* and *in vivo* approaches. Initial S-fuse cell culture assays screened a chemically diverse library of compounds for antiviral activity against SARS-CoV-2. Subsequent evaluations assessed the cytotoxicity of the most promising candidates, including **UMF** and its analogues. Following this primary screening, computational analyses employing Random Forest (RF) generative models guided the design of new synthesis candidates. Finally, chemical synthesis and experimental assays validated these designed compounds.

**Figure 1:**
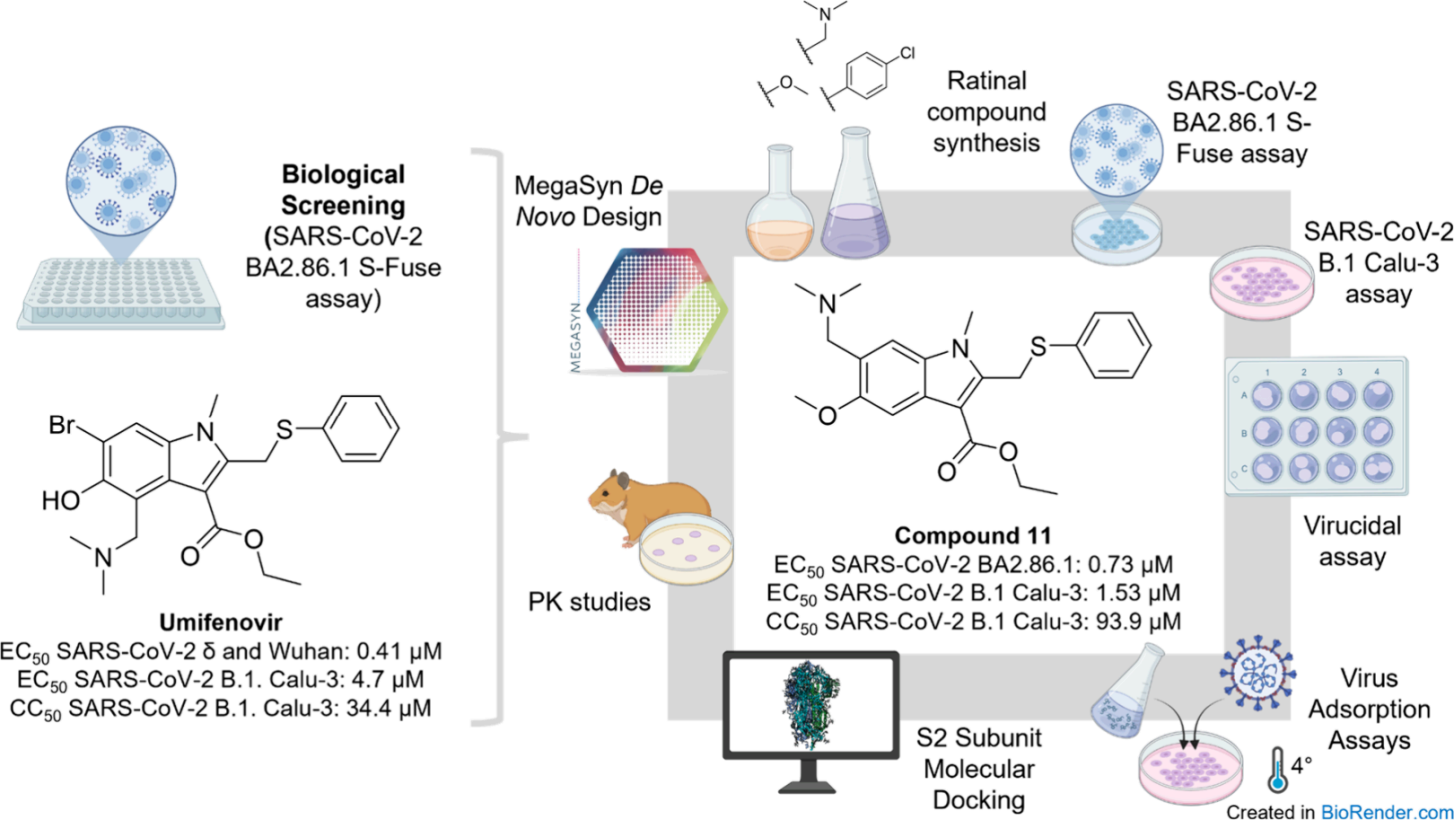
Schematic overview of the workflow employed to identify and evaluate new UMF analogs as inhibitors of SARS-CoV-2.

This approach aimed to predict binding affinities and interactions with the SARS-CoV-2 spike protein. Subsequent S-fuse and Calu-3 antiviral assays evaluated the most potent compounds. Further investigations employed virucidal assays to determine whether the compounds directly inactivate viral particles, while viral adsorption assays assessed their effects on the early stages of viral entry. Exploratory molecular docking at the SARS-CoV-2 spike S2 subunit served as a hypothesis-generating model. Finally, in vitro assays determined the metabolic stability of the most promising antiviral candidates in relevant liver microsomes, guiding the subsequent preclinical in vivo mouse pharmacokinetic (PK) evaluations.

### Screening of Initial Compounds via Antiviral S-Fuse Assays (Wuhan and Omicron Variants)

Initial screening of a diverse in-house chemical library, comprising over 5000 original compounds from the Research Center of Biotechnology (RAS), utilized the S-fuse assay^49^ to quantitatively measure viral inhibition against the SARS-CoV-2 Wuhan strain (Table 1). This early evaluation took place in September 2021; although no longer the predominant circulating variant at that time, the Wuhan strain served as a standard reference virus owing to its well-characterized *in vitro* profile and ancestral lineage status. Upon the availability of new strains, subsequent validation studies incorporated testing against Omicron variants, including BA.2.86.1. Ultimately, the assays yielded quantitative measurements of antiviral efficacy (EC_50_) and cytotoxicity (CC_50_) across both the Wuhan and Omicron lineages.

**Table 1:**
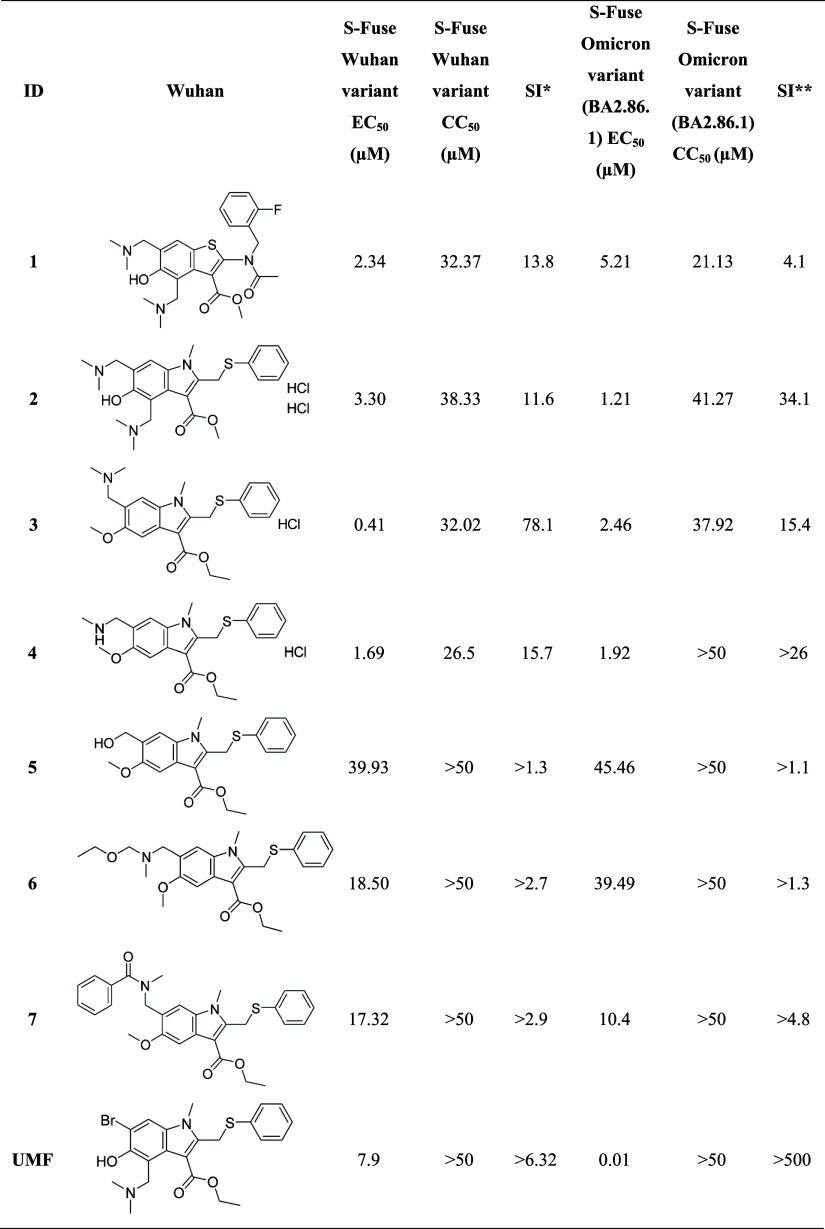
Antiviral Activity and Cytotoxicity of the First Round of Compounds against SARS-CoV-2 Wuhan and Omicron (BA2.86.1 Lineage) Variants^a^

a SI*: selectivity index for S-Fuse Wuhan variant (SI_Wuhan)_. SI**: selectivity index for S-Fuse Omicron BA2.86.1 variant (SI_Omicron_).

The screening protocol incorporated UMF as a benchmark for comparison with the structurally similar indole analogues. In the S-fuse Wuhan assay, UMF exhibited an EC_50_ of 7.9 μM alongside low cytotoxicity (CC_50_ ≥50 μM). Furthermore, the compound demonstrated significantly higher activity in the S-fuse Omicron assay, achieving an EC_50_ of 0.01 μM and maintaining high selectivity (CC_50_ >50 μM).

Compound **3**, a 4-*H*-5-methoxy-6-dimethylaminomethyl-substituted indole analogue of UMF, demonstrated the most potent Wuhan variant antiviral activity with an EC_50_ value of 0.41 μM and a CC_50_ value of 32.02 μM (SI of 78.1), which is ∼19-fold more active than UMF. Against the Omicron variant, **3** demonstrated an EC_50_ of 2.46 μM and a favorable CC_50_ of 37.92 μM (SI of 15.4). However, this is significantly less active than UMF (∼246-fold).

Additional indole analogues that demonstrated significant antiviral activity and adequate safety margins included compounds **1** (SI_Wuhan_ = 13.8 and SI_Omicron_ = 4.1), **2** (SI_Wuhan_ = 11.6 and SI_Omicron_ = 34.1), and **4** (SI_Wuhan_ = 15.7 and SI_Omicron_ >26). Although compounds **2** and **4** exhibited better EC_50_ and SI values against the Omicron variant, compound **3** showed the best balance of potency, selectivity, and structural features suitable for further derivatization. Moreover, it retained reasonable activity against Omicron (EC_50_ = 2.46 μM; SI = 15.4) and demonstrated cross-variant efficacy, including strong activity against the Delta variant (EC_50_ = 0.41 μM).

These promising initial *in vitro* results for the 4-*H*-5-methoxy-6-(di)­alkylamino methyl-substituted indole analogues **3** and **4** led to the selection of compound **3** as the prototype (hit) for structural optimization, substituent modification, and rational design. The resulting structural insights subsequently directed the proposal of a second round of compounds for synthesis, SAR evaluation, and biological assessment.

We also performed S-fuse neutralization assays against Delta and Omicron (XBB1.5 lineage) variants for several promising compounds (Supporting Information, Table S1). Compound **3** maintained a strong cross-variant efficacy against Delta (EC_50_ = 0.41 μM, CC_50_ = 32 μM). Compound **2** showed an EC_50_ of 0.5 μM and a CC_50_ of 1.3 μM for Delta. For Omicron XBB.1.5, compound **1** exhibited EC_50_ values of 1.69 μM and 2.34 μM, with CC_50_ values of 26.5 and >50 μM, respectively.

### ML Generative Models—*De Novo* Design

In our earlier-described MegaSyn generative method,^50,51^ integrated machine learning-based models focused on SARS-CoV-2, comprising 506 molecules (72 active and 434 inactive). A 5-fold nested cross-validation approach generated seven classification models from this data set, yielding the statistical metrics detailed in Table S2 (Supporting Information). The MegaSyn pipeline subsequently employed the Random Forest (RF) model. Using the most promising initial hit, compound **3**, the generative process produced 200 designs, available in the Supporting Information. Subsequent analysis of these 200 MegaSyn-derived designs identified two structures (13 and 18) for synthesis and follow-up experimental evaluation (Figure 2). These compounds combined high predicted activity scores with structural modifications that specifically block known metabolic hotspots of UMF.

**Figure 2:**
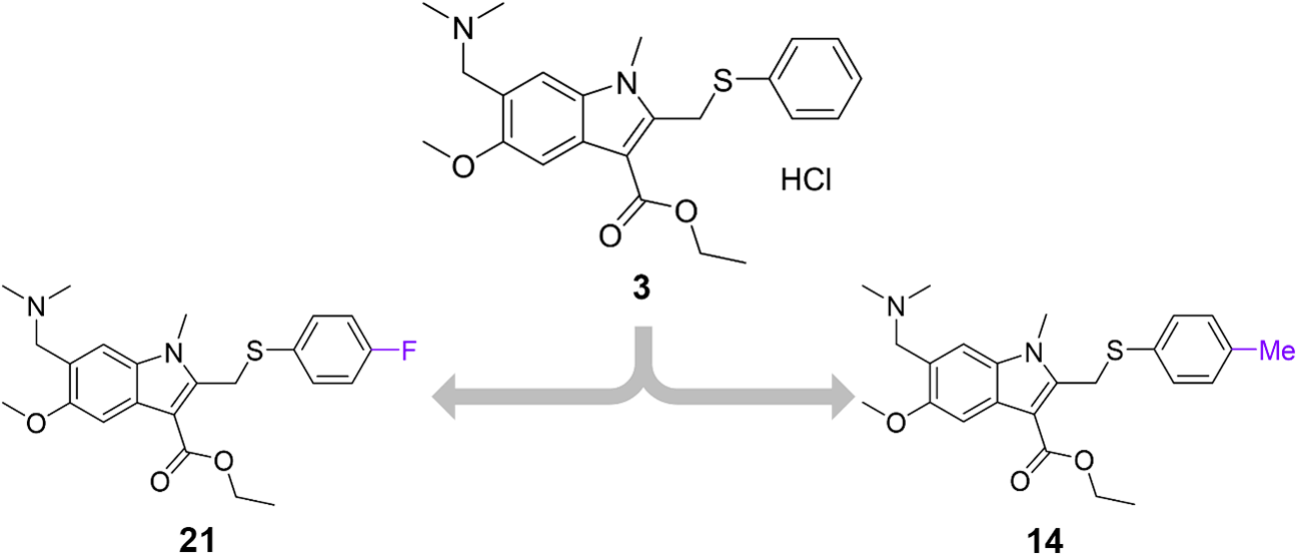
New molecular structures designed using the MegaSyn generative approach. The purple groups represent the structural modifications suggested by the MegaSyn model to optimize the hit’s properties.

### Rational Design

Beyond exploring structure–activity relationships (SAR) to enhance antiviral activity and selectivity against COVID-19, the optimization process targeted several known limitations of UMF. Previous reports highlight various drawbacks of UMF, including poor human pharmacokinetics (PK),^46,47^ low oral bioavailability, a short human half-life,^48^ rapid metabolism,^46^ and diverse side effects.^46^ Furthermore, metabolic profiling also identifies both O-glucuronide and O-sulfate conjugates as major metabolites within human plasma.^52^

Given these literature findings and building on our initial screening results and ML generative modeling, we designed UMF analogs without the free 5-hydroxyl group and prioritized the derivatization of 5-methoxyindoles.

The structural design strategy targeted two distinct regions of the indole core scaffold (Figure 3) for modification. Left-hand-side (LHS) optimization involved substitutions at the 4′-position on the indole core (R_1_) alongside modifications of the 5′- and 6′-positions (R_2_ and R_3_, respectively). In contrast, right-hand-side (RHS) modifications focused on exploring different alkyl substituents at the indole nitrogen (1′-position/R4) and on derivatizing the thiophenol moiety (2′-position/R5).

**Figure 3:**
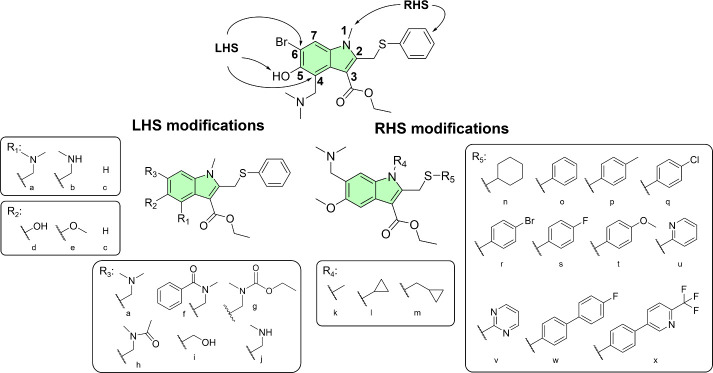
Proposed structural modifications on the UMF indole core scaffold for synthesis and screening against SARS-CoV-2.

To evaluate the impact of positional changes on *in vitro* antiviral activity, the molecular design strategy targeted structural isomer derivatives retaining the basic amino group at R_1_, based on the UMF core (RHS). Consequently, the synthesis of several structural isomers (Figure 3a**–**c) probed the influence of these modifications while preserving the essential pharmacophore. Initial screening results identified the R_2_ hydroxy group (Figure 3c) as a metabolic liability. Conversely, data indicated that the methoxy group (Figure 3d) retained sufficient antiviral activity, comparable to that of the ether analogue (Figure 3e).

Additionally, structural modifications at the R_3_ position incorporated more prominent and sterically rigid cycloalkyl groups (Figure 3a,f–j) to prevent potential metabolic demethylation of the indole nitrogen and further enhance antiviral activity. Subsequent analogue designs adopted this modification as a core element, serving as a proactive measure to prevent O-glucuronide conjugation and improve pharmacokinetic (PK) properties. Further exploration at R_3_ assessed the essentiality of the basic amino group by introducing nonbasic amide alternatives and substituting the nitrogen with an oxygen (Figure 3g). This strategy evaluated the importance of the moiety’s hydrogen-bond acceptor properties and determined whether basicity itself acts as a prerequisite for activity.

Moreover, methyl and methylcyclopropane substituents at R_4_ (Figure 3k–m) probed potency differences arising from increased hydrophobicity and steric bulk, factors that directly modulate the overall PK profile.

At R_5_, structural diversification explored the essentiality of the lipophilic phenyl substituent on the thiomethyl moiety by incorporating nonaromatic and heteroaromatic groups, as well as specific phenyl ring substitutions. Supported by machine learning (ML)-based generative modeling (Figure 2), these changes investigated impacts on antiviral activity, influenced solubility, and aimed to block potential metabolic oxidation of the phenyl ring. Furthermore, fluorine substitutions (Figure 3s,w,x) served a strategic purpose beyond merely altering the electronic and spatial properties to impact activity. Replacing hydrogen atoms with fluorine systematically blocks metabolism, as the strong carbon–fluorine (C–F) bond resists cytochrome P450 (CYP)-catalyzed oxidative metabolism, ultimately improving metabolic stability and prolonging the *in vivo* half-life of the compounds.

### Synthesis of Target Compounds

The synthesis of target compounds featuring R_1_ and R_2_ substituents used 5-methoxyindole intermediates bearing either a methyl, cyclopropyl, or cyclopropylmethane *N*-alkyl group as starting materials. Standard literature procedures^53–55^ yielded these common intermediates from ethyl acetoacetate, which were subsequently radical-chlorinated to afford the desired chloromethylindole intermediates, as outlined in Scheme 1. Finally, a substitution reaction with the appropriate 2′-position thiolate, followed by the addition of the key dimethylamino methane group under Mannich reaction conditions, produced the targeted indole analogues.

**Scheme 1:**
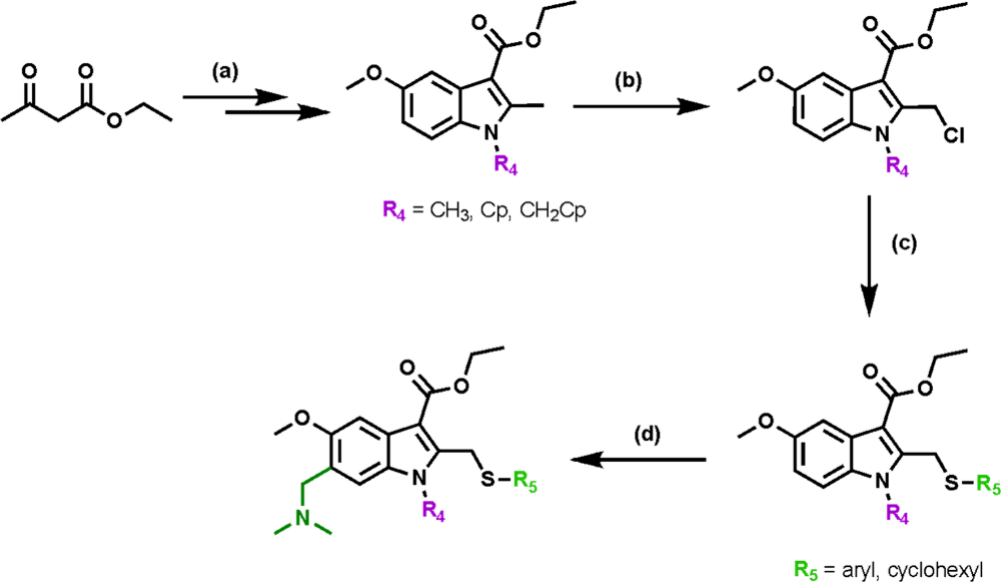
General Synthetic Strategy for the Synthesis of Targets with R_1_ and R_2_ Modifications^a^

a Reagents and conditions: (a) Literature-reported procedures;^53–55^ (b) NCS, ABCN, CHCl_3_; (c) (i) R_4_SH, KOH, MeOH, (ii) chloroindole; (d) Me_2_NCH_2_NMe_2_, AcOH.

### Screening of UMF Analogues via Antiviral S-Fuse Assays (Omicron Variant)

The second round of compound screening evaluated a new set of 25 synthesized compounds, proposed by the generative models and rational design, against the SARS-CoV-2 Omicron variant (BA.2.86.1 lineage) using the S-Fuse assay (Table 2). Remdesivir (RDV), a broad-spectrum antiviral drug, was used as a positive control due to its established efficacy and well-characterized antiviral properties.^56,57^

**Table 2:**
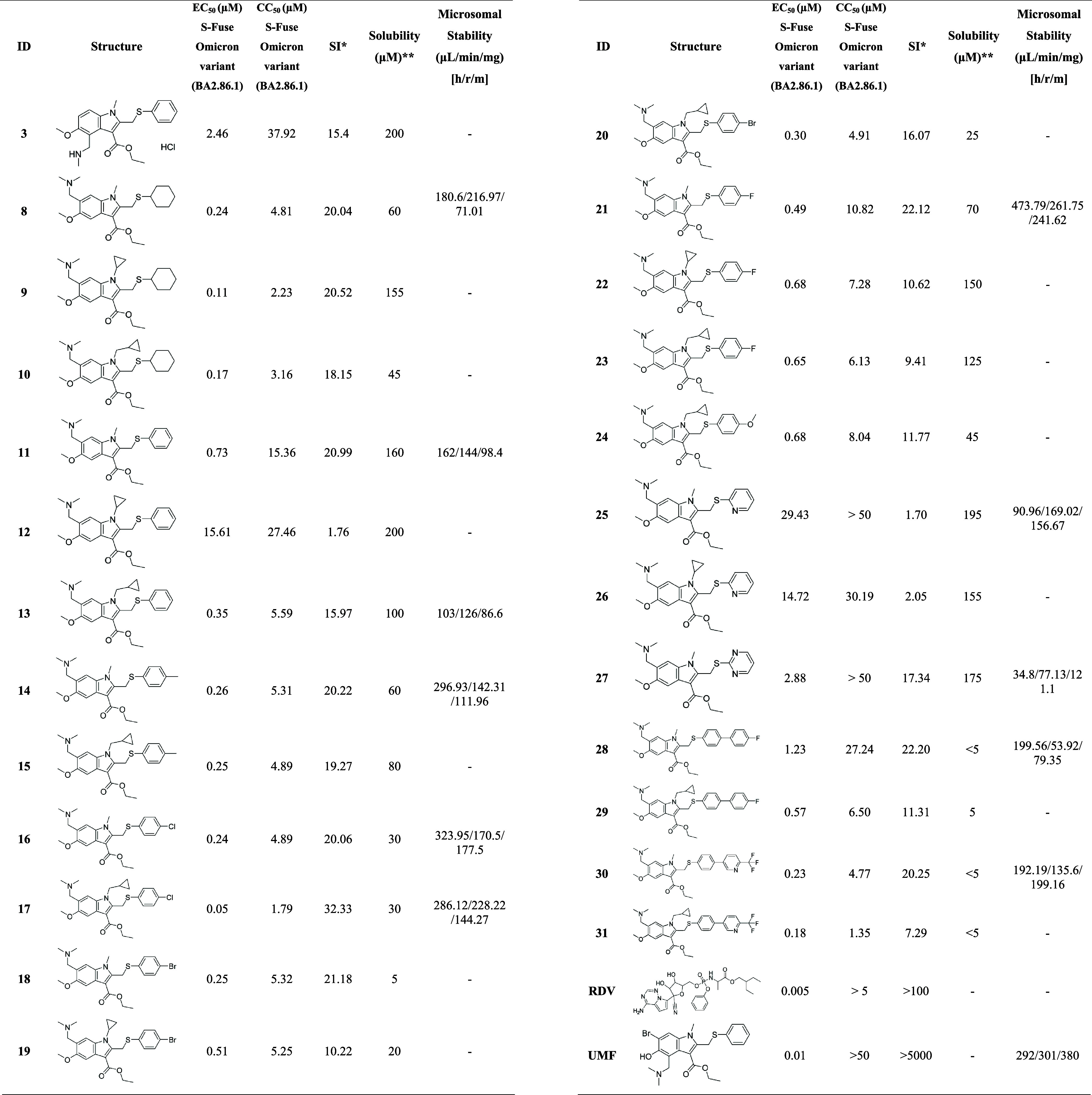
Antiviral Activity and Cytotoxicity of the Second Round of UMF Analogues against the Omicron Variant (BA2.86.1 Lineage)^a^

a SI**: selectivity index for S-Fuse test on Omicron BA2.86.1 variant (SI_Omicron_)**PBS at pH 7.4.

Table 2 summarizes the antiviral activity and physicochemical properties of the screened compounds. Candidates **8**, **9**, **11**, **14**, **16**, **21**, and **30** exhibited high potency (EC_50_ <0.5 μM) and favorable selectivity indices (SI ≥20). Among these, compound **11** stands out due to its balanced profile of antiviral potency (EC_50_ = 0.73 μM), selectivity (SI = 20.99), high solubility (160 μM), and moderate microsomal turnover. Similarly, compound **8** showed promising results (EC_50_ = 0.24 μM; SI = 20.04). In contrast, compound **17** exhibited high clearance in human liver microsomes, suggesting rapid metabolism and potential pharmacokinetic (PK) limitations. Although compound **9** demonstrated significant potency (EC_50_ = 0.11 μM; SI = 20.5), limited material availability precluded further investigation.

Structure–activity relationship (SAR) analysis of R_1_ and R_2_ indole derivatizations indicates that replacing a dimethylamino group with a methylamino group reduces potency, as evidenced by the EC_50_ increase from 0.73 to 1.92 μM. Further substitutions with phenyl formamide, ethoxy methylamine, methylacetamide, or hydroxymethyl groups caused a 10- to 40-fold decrease in activity (EC_50_ values ranging from 10.4 to 45.46 μM), underscoring the requirement for a protonatable group to maintain high potency. Regarding the indole N-substitution (R_4_), SAR data reveal that increasing lipophilicity through larger aliphatic groups (N-Me < N-cyPr < N–CH2-cyPr) proportionally enhances activity against both the virus and host cells. This trend suggests that compound permeability, rather than specific drug–target interactions, primarily drives the observed biological effects.

Derivatization of the thiophenol group (R_5_) with electron-withdrawing groups, such as chlorophenyl and trifluoropyridinyl, significantly increases potency. However, incorporating electronegative rings, such as 3- or 4-methoxyphenyl and fluorophenyl, increases cytotoxicity by up to 20-fold. Consequently, based on the integrated profiles of potency, selectivity, solubility, and stability, this study prioritized compounds **11**, **18**, **13**, **23**, and **3** for additional biological evaluation in Calu-3 human lung epithelial cells.

### Inhibition of SARS-CoV-2 on Human Lung Epithelial Calu-3 Cells

Following the S-Fuse assays, subsequent evaluations advanced the promising compounds (**3**, **11**, **13**, **18**, and **23**) to the human lung epithelial cell line Calu-3, a relevant model that recapitulates type II pneumocytes.^58,59^ The initial screening assessed these candidates at a 10 μM concentration in SARS-CoV-2-infected Calu-3 cells. Plaque assays quantified infectious virus particles as plaque-forming units per milliliter (PFU/mL) and reported the final data as a percentage of inhibition (Figure 4A). In this single-dose test, the compounds inhibited virus replication by >85%, except for a single replicate of compound **13**. All novel **UMF** analogues are more effective than the reference compound, which aligns with previous assays directed toward the S protein. RDV at 10 μM inhibits 99.9% of SARS-CoV-2 B.1. lineage production, in line with other published studies^57,60^ as a control for virus inhibition.

**Figure 4:**
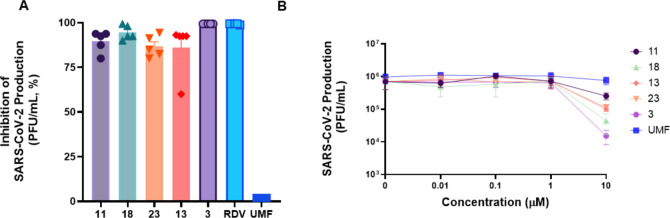
Antiviral activity of UMF analogues against SARS-CoV-2 in Calu-3 cells. (A) Initial screening evaluated selected compounds against SARS-CoV-2 at 10 μM. (B) Subsequent dose–response assays assessed the inhibitory molecules at various concentrations, enabling calculation of EC_50_ values. RDV and UMF served as positive controls throughout the experiments.

Given the logarithmic replication of viruses such as SARS-CoV-2, dose–response assays evaluated the effect of the compounds on viral production at concentrations ranging from 0.01 to 10 μM. Figure 4B displays the viral RNA copies on a logarithmic scale to emphasize the pronounced antiviral effects at higher concentrations. This representation, however, limits the visualization of moderate inhibitory effects, particularly those below 90%. To overcome this limitation, Figure S2 displays the same data set on a linear scale, with the percentage of viral replication shown.

Figure 4B displays the original virus titers in PFU/mL. Compound **11** exhibited substantial activity, reducing viral titers by 2 log10 (99%). Regarding antiviral potency, the half-maximal effective concentrations (EC_50_) indicate that all evaluated compounds retain activity in the low micromolar range, with compounds **11** and **18** emerging as the most potent (Table 3). Furthermore, cytotoxicity assessments indicate that while compound **3** exhibits moderately higher toxicity than other **UMF** analogues, compounds **11** and **18** demonstrate a superior safety profile with markedly lower cytotoxicity (Table 3).

**Table 3:** Antiviral Activity, Cytotoxicity, and SI of UMF Analogues in Calu-3 Cells Infected with SARS-CoV-2 B.1. Lineage

**compound**	**EC**_**50**_ **(μM)**	**CC**_**50**_ **(μM)**	SI***^a^
**11**	1.53	93.9 ± 4.7	61.37
**18**	1.40	56.4 ± 2.8	40.3
**13**	7.7	37.3 ± 1.8	4.84
**23**	9.9	42.1 ± 2.1	4.25
**3**	4.7	34.4 ± 1.7	7.3
**UMF**	5.7	85.5 ± 4.2	15

a SI***: selectivity index for antiviral Calu-3 cells.

Compound **11** exhibited the most favorable antiviral activity and cytotoxicity profile among the tested compounds, achieving an SI of 61.37 (Table 3), indicating a substantial difference between its CC_50_ and EC_50_ values. In comparison, compound **18** demonstrated notable potency with an EC_50_ of 1.4 μM, a CC_50_ of 56.4 ± 2.8 μM, and an SI of 40.3, which, while significant, is lower than that of compound **11** (Table 3).

### Virucidal Assays

To investigate potential direct effects on SARS-CoV-2 virions, the experimental protocol preincubated viral particles with compounds **11**, **18**, **UMF**, and **RDV** at concentrations ranging from 0.01 to 10 μM before Calu-3 cell infection. After 48 h, plaque assays quantified the viral titers in the supernatant. As shown in Figure S5, none of the compounds significantly reduced viral infectivity, confirming a lack of virucidal activity across all evaluated concentrations. While previous Calu-3 antiviral assays establish that compounds **11** and **18** effectively inhibit SARS-CoV-2 replication in human lung epithelial cells, these virucidal results rule out direct viral inactivation. Consequently, the findings suggest that intracellular or entry-associated mechanisms mediate the observed antiviral efficacy.

### Virus Adsorption Assays

To investigate whether compound **11**, which exhibited the most favorable antiviral activity, interferes with SARS-CoV-2 entry at the attachment stage, the molecule was added before and during infection at 4 °C (Figure 5). The experimental protocol utilized compound concentrations of 0.01, 0.1, 1, and 10 μM, matching those employed in the antiviral assays. Compounds **11** (Figure 5A) and the control **UMF** (Figure 5B) did not impair viral adsorption in any of the conditions tested.

**Figure 5:**
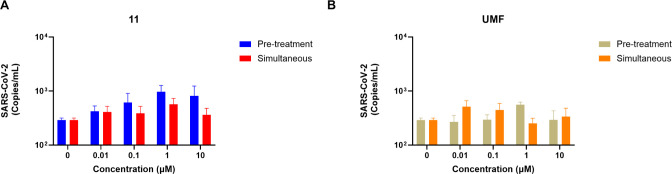
Effects of compounds **11** and UMF on viral adsorption. The experimental protocol exposed SARS-CoV-2 (MOI = 0.5) to varying concentrations (0.01, 0.1, 1, and 10 μM) of compound **11** (A) and **UMF** (B), either as a 1 h pretreatment at 37 °C or concurrently during a 1 h infection phase at 4 °C. Subsequent lysis of the cell monolayers enabled virus titration via RT-qPCR. *P < 0.05.

The adsorption assays, which selectively probe the attachment step of viral entry, showed that neither compound **11** nor **UMF** significantly impaired viral adsorption. Taken together, these results indicate that the antiviral activity of compound **11** is not associated with virucidal effects or inhibition of viral attachment but instead occurs at a later stage of the infection process, either during downstream entry events (such as fusion or internalization) or at postentry steps of the viral replication cycle.

### Exploratory Molecular Docking at the SARS-CoV-2 Spike S2 Subunit

Previous studies have shown that UMF binds to the spike protein, particularly the S2 subunit, and prevents the conformational changes required for membrane fusion without inactivating the virus itself.^38–41^ Accordingly, the observed absence of virucidal activity fully aligns with a nonvirucidal, fusion-related mode of action. Guided by this experimental evidence, exploratory molecular docking served as a hypothesis-generating approach to investigate potential interactions between the UMF derivatives and structural regions of the spike protein involved in the fusion step of viral entry.

The SARS-CoV-2 spike protein contains several critical regions suitable for small-molecule binding,^61^ primarily within the S1 and S2 subunits. In the S1 subunit, the RBD is a primary target, with compounds binding near residues essential for ACE2 recognition, potentially stabilizing the spike’s closed conformation. The S1/S2 interface is another key site where compounds can interfere with conformational rearrangements required for membrane fusion. In the S2 subunit, the heptad repeat regions (HR1/HR2) support fusion; disruption of the six-helix bundle (6HB) assembly within this region effectively blocks viral entry. Additionally, ligands targeting the prefusion S2 region may interfere with spike trimerization and overall structural stability, thereby hindering membrane fusion.

Consistent with this mechanistic framework, previous literature characterizes several small molecules as entry inhibitors targeting the conserved S2 subunit.^61^ Specifically, toremifene^62^ binds to the S1/S2 interface and stabilizes the prefusion state of the spike protein, thereby preventing fusion. Salvianolic acid C^63^ disrupts 6HB assembly within the S2 subunit, a critical step in membrane fusion. Similarly, antifungal agents such as posaconazole^64^ and itraconazole^65^ inhibit the 6HB core, exhibiting broad-spectrum potential against various SARS-CoV-2 variants. Furthermore, navitoclax targets the HR1 region to block 6HB formation, effectively preventing infection across multiple viral strains.^66^

A study by Shuster and co-workers^39^ demonstrated that **UMF** inhibits the entry of SARS-CoV-2 spike protein-pseudotyped viruses (PVs) into ACE2-expressing human cells. Evaluations against variants such as the Wuhan, UK (B.1.1.7), and South African (B.1.351) strains revealed that **UMF** blocks the cell entry of S protein-pseudotyped MLV viruses with a half-maximal effective concentration (EC_50_) of approximately 5 μM. Mechanistically, this inhibitory effect likely stems from **UMF** binding directly to the spike protein, destabilizing the macromolecule and promoting its lysosomal degradation, ultimately preventing the conformational changes necessary for viral infection.

To identify the **UMF** binding site on the spike protein, researchers used limited proteolysis coupled with mass spectrometry (LiP-MS),^39^ along with molecular docking and molecular dynamics simulations. This study revealed that **UMF** protected the S2 domain region from proteolytic digestion and induced thermal destabilization of the spike protein, as demonstrated in thermal shift assays (TSA), suggesting that this interaction compromises the protein’s structural stability and promotes lysosomal degradation in infected cells.^39^ The binding site is located on the S2 subunit region (residues 1021A, 1024A, 1027A, 1062A, and 1064A), an essential region for the membrane fusion and spike trimerization.

Based on this experimentally validated **UMF** binding site, we performed molecular docking studies to explore the potential binding modes of the **UMF** derivatives investigated in this work (Figure 6). Docking calculations utilized the SARS-CoV-2 spike protein trimer in the prefusion apo form (PDB ID: 6VXX)^67^ (Figure 6A). These calculations focused on the amino acids previously reported to destabilize the S2 subunit upon ligand binding: Ser1021A, Leu1024A, Thr1027A, Phe1062A, and His1064A.^39^

**Figure 6:**
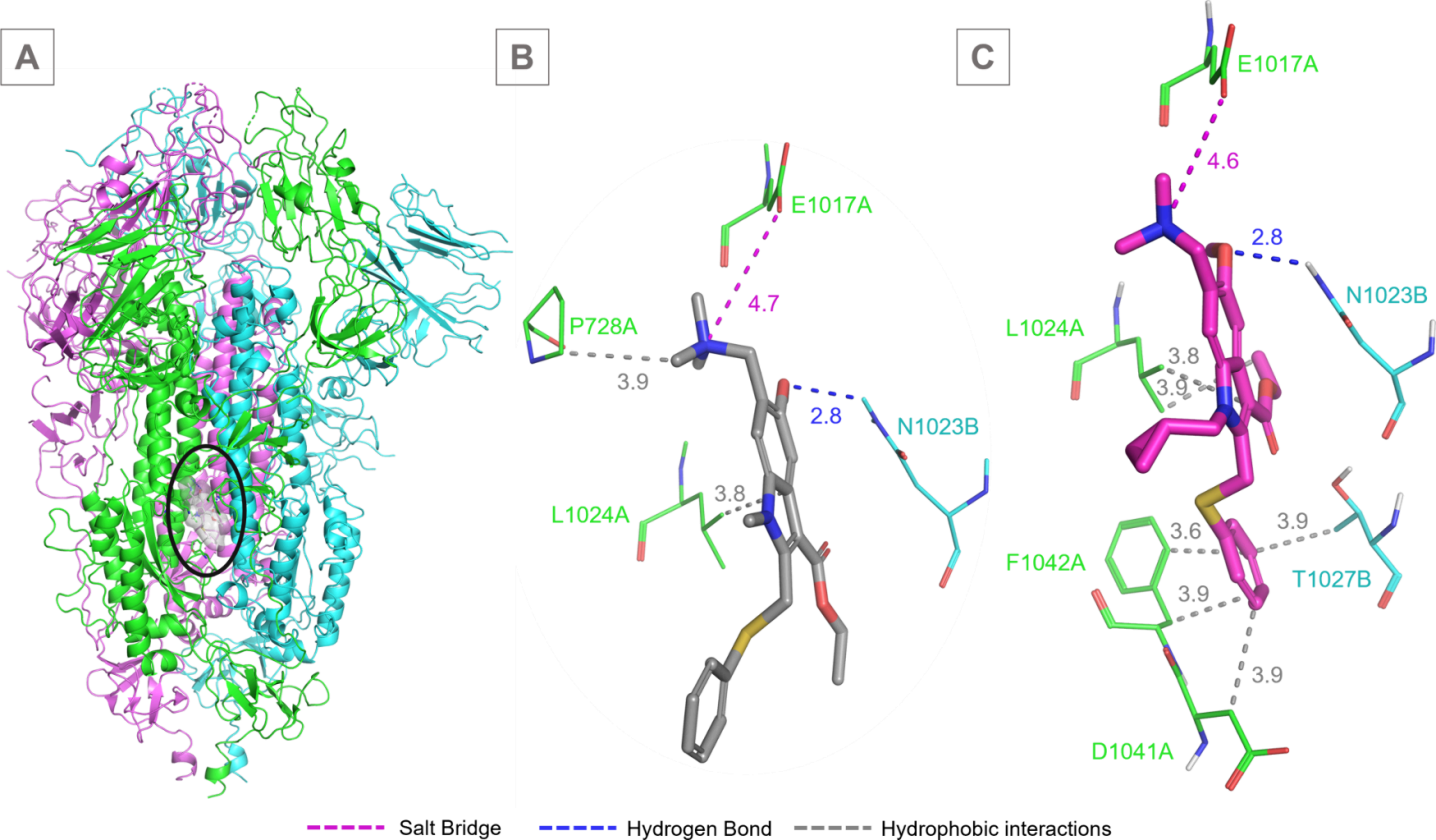
Predicted molecular interactions of compounds **11** and **UMF** with the S protein by docking calculations. (A) Overall structure of the SARS-CoV-2 spike protein trimer, with each monomer in a different color (green, cyan, and magenta). The binding site for the compounds on the S2 subunit is highlighted by a black circle, with zoomed views provided for detailed interaction analysis. (B) Compound **11** (carbon in gray) and (C) **UMF** (carbon in magenta) with the SARS-CoV-2 spike protein S2 subunit, predicted by molecular docking.

Molecular docking simulations predict that compound **11** forms a hydrogen bond with Asn1023B, a salt bridge with Glu1017A, and hydrophobic interactions with Pro728A and Leu1024A (Figure 6B). In comparison, the reference drug **UMF** establishes a hydrogen bond with Asn1023B, salt bridges with Glu780B and Lys776B, and an extended network of hydrophobic interactions involving Leu727A, Leu1024A, Phe1042A, Thr1027B, Ala1026B, and Arg1019B (Figure 6C).

A comparison of the protein–ligand interaction profiles revealed that both **UMF** and compound **11** conserve the hydrogen bond with Asn1023B. However, compound **11** replaces the **UMF**-chain B salt bridge with a new interaction involving Glu1017A. Additionally, both molecules retain only the hydrophobic interactions with Leu1024A. Overall, the number of hydrophobic contacts decreases from six in **UMF** to two in compound **11**, a reduction that likely contributes to differences in binding affinity and antiviral potency. Furthermore, **UMF** displays protonation at both the hydroxyl and tertiary amine groups, whereas compound **11** exhibits protonation solely at the tertiary amine within the aliphatic group.

Molecular docking predictions suggested that compound **11** may interact with the S2 subunit, interacting with residues previously associated with conformational rearrangements required for membrane fusion. Previous studies have shown that UMF binds to the S2 subunit and interferes with spike-mediated membrane fusion without directly inactivating viral particles.^39–41^ In agreement with this mechanism, our virucidal assays demonstrated that neither compound **11** nor **UMF** directly inactivates SARS-CoV-2 virions. Accordingly, these docking results should be regarded as hypothesis-generating, suggesting a potential role for compound **11** in later stages of viral entry rather than in direct viral inactivation.

### Physicochemical Properties, *In Vitro* Microsomal Stability, and *In Vivo* Pharmacokinetics

The improved antiviral activity in infected Calu-3 cells and the higher selectivity index (SI) of compound **11** (Table 3) prompted its advancement to mouse pharmacokinetic (PK) evaluation, alongside the antiviral drug **UMF** as a benchmark (Table 4). Furthermore, compound **11** exhibited a 10-fold increase in solubility at pH 7.4 relative to **UMF** (Table 2).

**Table 4:** Solubility, Microsomal Stability, and Low-Dose Mouse Pharmacokinetic Data of **UMF** and Compound **11** ^a^

	**11**	**UMF**
solubility at pH 7.4 (μM)	160	15
microsomal CL_int,app_ (mL/min/kg) [h/r/m]	200/260/387	361/541/1497
hPPB (*F*_u_)	0.07	0.03

mouse PK	**IV (48 h)**	**PO (48 h)**	**IV (48 h)**	**PO (48 h)**
*C*_max_ (μM)		0.03		0.04
*T*_max_ (h)		0.7		0.5
*T*_1/2_ terminal (h)	7.2	21.5	1.5	0.9
mouse CL_b_(mL/min/kg)	81.5		137.8	
mouse CL_u_(mL/min/kg)	1164		4593	
*V*_ss_(L/kg)	16.5		10.2	
*V*_ss,u_(L/kg)	236		340	
AUC 0–t (min·μmol/L)	96	8	66	4
*F* (%)		3.3		1.9

a Microsomal stability in human, rat, and mouse (h/r/m); hPPB, human plasma protein binding shown as the fraction unbound (*F*_u_); PO, oral dose of 10 mg/kg; IV, intravenous dose of 3 mg/kg; *C*_max_, maximum concentration; *T*_max_, time required to reach the maximum concentration; *t*_1/2_, elimination half-life; *V*_ss_, apparent volume of distribution at steady state; *V*_ss,u_, unbound apparent volume of distribution at steady state; CL_b_, whole-blood clearance; CL_u_, unbound whole-blood clearance AUC_0–*t*_, an area under the curve from time 0 to the last experimental time point; *F*, bioavailability.

*In vivo* PK studies evaluated **UMF** and compound **11** in healthy male BALB/c mice following intravenous (IV) and oral (PO) administration at 3 and 10 mg/kg, respectively (Table 4). Consistent with *in vitro* data, both compounds exhibited high total blood clearance. Interestingly, adjusting the total blood clearance values to determine *in vivo* unbound clearance revealed that compound **11** achieves a 4-fold improvement over **UMF**. This enhanced *in vivo* unbound clearance correlates strongly with the *in vitro* mouse liver microsome data (Table 4).

The increase in solubility and the reduction in unbound clearance resulted in a slight improvement in oral bioavailability, indicating that future compounds must improve *in vitro* microsomal stability to achieve meaningful gains in *in vivo* mouse exposure levels. Accordingly, beyond synthesizing further analogues aimed at systematically mitigating the structural metabolic hotspots identified in this chemotype, future studies should focus on improving intrinsic metabolic stability through rational MedChem optimization, including modulation of lipophilicity, rigidity, and steric protection around labile regions. After identifying a more advanced, metabolically stable hit, strategies such as prodrug approaches or specialized formulation technologies may be considered.

To complement these findings, we included plasma concentration–time profiles for both UMF and compound **11** in Figures S3 and S4. These curves provide a visual assessment of *in vivo* exposure relative to the antiviral EC_50_ values, supporting the interpretation of systemic exposure limitations observed in Table 4.

## Conclusions

This study successfully applied integrated computational and experimental approaches to identify and optimize novel UMF analogues with promising antiviral activity against SARS-CoV-2 and improved pharmacokinetic properties. Inspired by structural variations, generative models, and rational synthesis strategies, enhanced antiviral efficacy while maintaining low cytotoxicity. Among the tested compounds, compound **11** emerged as the most promising hit candidate, exhibiting a satisfactory balance of potency, safety, and selectivity. Unfortunately, compound **11** demonstrated only limited improvements in mouse PK compared with the antiviral drug UMF, including increased aqueous solubility at physiological pH, a prolonged terminal half-life, and increased systemic exposure. Overall, the antiviral effect of compound **11** is not associated with virucidal activity or viral attachment inhibition, but rather with later stages of the infection process. Consistent with this interpretation, the molecular docking results should be regarded as hypothesis-generating, suggesting a potential involvement of compound **11** in downstream viral entry events rather than in direct viral inactivation.

In conclusion, these findings provide a foundation for advancing **11** as a hit for further diversification and development, including possible formulation studies, to identify compounds with further enhanced metabolic stability and systemic exposure and, ultimately, to support the expansion of antiviral strategies against SARS-CoV-2 variants.

## Methods

### Experimental Section

#### S-Fuse Neutralization Assay

U2-OS Ace2 GFP 1–10 and 1–11 (terms S-Fuse cells) were split the day of infection.^49^ The molecules and viruses are preincubated for 2 h at 37 °C. Briefly, 100 μL of molecules was diluted in growth medium (DMEM, 10% FCS, 1% PS), and 10 μL of virus (either D614G or BA.2.86) was added per well (final MOI 0.1). The final DMSO concentration was at 1% or lower. After this preincubation, the mixture of molecules and viruses was added to the cells for a 20 h incubation at 37 °C, followed by fixation with 3% PFA for 30 min at RT and a final wash with PBS. To stain the nuclei and measure viability, 100 μL of Hoechst solution was added to each well. The plates were read with an automated confocal microscope (Opera Phoenix), which measures infection (number of syncytia per well, quantified by GFP signal) and viability (number of nuclei per well, quantified by Hoechst signal). The analysis was performed using the Harmony Software, with a fixed threshold determined from the signal obtained in noninfected conditions. The EC_50_ values were calculated in GraphPad Prism using a sigmoidal 4PL model with X as the Log Concentration. The top and bottom were constrained to 100% and 0% inhibition, respectively.

#### General Synthesis Methods

All reagents and solvents were purchased from commercial suppliers and used without further purification. ^1^H and ^13^C spectra were recorded on a Bruker AC-200 (200 MHz, ^1^H; 50 MHz, ^13^C) NMR spectrometer. Chemical shifts were measured in DMSO-*d*_6_ with tetramethylsilane as an internal standard and were reported in ppm. High-resolution mass spectrometry (HRMS) analysis was performed on an Impact II QqTOF high-resolution mass-spectrometer equipped with an Apollo II ESI ion source according to the following conditions: direct sample infusion at a rate of 0.25 mL/min, ion source in positive mode, high-voltage capillary at 4.5 kV, spray gas–nitrogen at 2.5 bar, dry gas–nitrogen at 6 L/min 220 °C, scan range *m*/*z* 50–1500, 3 Hz scan rate, and automatic internal calibration with sodium trifluoroacetate solution. Spectra were processed with Compass DataAnalysis 5.1. The purity of the final compounds was analyzed by analytical high-performance liquid chromatography (HPLC) on an Elute HPLC system equipped with an Azura UVD 2.1S UV detector set at 254 nm and an acquisition rate of 1 Hz. Chromatographic separation was carried out on an ACQUITY HSS T3 column (2.1 × 100 mm, 1.3 μm, 100 Å) at 30 °C, with a sample injection volume of 2.0 μL. A mobile phase consisting of 0.1% formic acid in water (A) and 0.1% formic acid in acetonitrile (B) was programmed with a gradient elution from 30 to 95% at a flow rate of 250 μL/min. Mass spectrometric detection was operated in positive-ion mode. Data were processed using Compass DataAnalysis 5.1 software. All final compounds were >95% pure. Melting points were determined on an Electrothermal 9001 melting point apparatus (10 °C/min) and were uncorrected. Merck KGaA silECa gel 60 F_254_ plates were used for analytical thin-layer chromatography. A UV lamp was used to detect spots. Column chromatography was performed using silica gel Merck 60 (70–230 mesh). Yields were reported for purified products and were not optimized.

1-Methyl-2-phenylmercaptomethyl-3-carbethoxy-5-methoxyindole and 1-methyl-2-phenylmercaptomethyl-3-carbethoxy-5-hydroxyindole were synthesized according to the procedure described by Trofimov et al.^68^

#### Antiviral Calu-3 Assays

SARS-CoV-2 replication inhibition assays were performed in Calu-3 cells. To this end, cells were seeded in 96-well culture plates (2 × 10^4^ cells per well) and, after 96 h, Calu-3 cells were infected with SARS-CoV-2 (B.1 lineage—GenBank #MT710714) at an MOI of 0.1 for 1 h at 37 °C. The inoculum was removed, and the cells were treated with the compounds at 10, 1, 0.1, and 0.01 μM diluted in a fresh culture medium. RDV and UMF were used as positive controls for inhibiting viral replication. After 48 h, virus-containing supernatants were collected and titrated by plaque assay.

All experiments were carried out at two independent times, and each data set was analyzed using Prism GraphPad software 10 (GraphPad Software, San Diego, California, USA). Triplicate experiments were performed for each data point, and the value was presented as mean ± standard deviation (SD). EC_50_ values for antiviral activity were calculated by nonlinear regression using a four-parameter logistic model (inhibitor vs. response) in GraphPad Prism, with concentrations analyzed on a logarithmic scale.

Viral inhibition was quantified based on plaque assay titers. Percent inhibition of viral replication was calculated relative to infected untreated controls, which were defined as 0% inhibition. Antiviral activity was expressed as the reduction in viral production (PFU/mL) compared with this control condition.

#### Quantification of Viral Titers by Plaque Assay

Viral production in the supernatants of Calu-3 cultures used *in vitro* assays was quantified through plaque formation assays. Vero E6 cells were infected with different dilutions of supernatants for 1 h at 37 °C, and after this period, a culture medium containing 2.4% carboxymethylcellulose was added. After 72 h, cells were fixed with 3.7% formalin and stained with 0.04% crystal violet. Cell lysis plaques were counted, and viral titers were determined in plaque-forming units per mL (PFU/mL).

#### Cell Viability Assay

Monolayers of Calu-3 cells (1.5 × 10^4^ cells/well) in 96-well culture plates were incubated with different concentrations of the compounds (50–500 μM) in DMEM High medium with 10% FBS for 48 h. Then, the dye resazurin was added (20 μL/well) to the wells. After incubation for 4 h at 37 °C, the plates were read in a fluorimeter at 560 nm (excitation) and 590 nm (emission). The 50% cytotoxic concentration (CC_50_) was calculated by performing a regression analysis on the dose–response curves generated from the data.

#### Virucidal Assays

Virucidal assays were performed in Calu-3 cells. To this end, cells were seeded into 96-well culture plates (2 × 10^4^ cells per well), and the assay was performed after 96 h. Compounds **11**, **18**, **UMF**, and **RDV** in different concentrations (10, 1, 0.1, and 0.01 μM) diluted in a fresh culture medium were incubated with a viral suspension of 0.5 × 10^5^ PFU of SARS-CoV-2 B.1 lineage (GenBank #MT710714) for 1 h at 37 °C. Then, Calu-3 cells were infected with the mixture of molecules and virus for 1 h at 37 °C. The inoculum was removed, and fresh culture medium was added to the cells. The fresh medium added after removing the inoculum did not contain the compound, ensuring that the assay evaluated virucidal activity rather than continuous drug exposure. After 48 h, virus-containing supernatants were collected and titrated by plaque assay.

#### Inhibition of the Adsorption Assay

An adsorption assay was performed in Calu-3 cells. To this end, cells were seeded into 96-well culture plates (2 × 10^4^ cells per well), and the assay was performed after 96 h. The compounds **11** and **UMF** in different concentrations (10, 1, 0.1, and 0.01 μM) were added in two conditions: (i) pretreatment of the cells for 1 h at 37 °C (diluted in a fresh culture medium with 10% of FBS) followed by incubation with the virus at 4 °C for 1 h or (ii) treatment simultaneously with virus incubation for 1 h at 4 °C. The MOI used was 0.5 of SARS-CoV-2 B.1 lineage (GenBank #MT710714). After virus incubation, the inoculum was removed, cells were washed with PBS, and the monolayer was lysed with lysis buffer from the Maxwell RSC Viral Total Nucleic Acid Purification Kit. After nucleic acid extraction, an RT-qPCR was performed with GoTaq Probe qPCR and RT-qPCR Systems. At temperatures below 4 °C, the virus can only bind to cells but cannot enter them.

#### In Vitro ADME Assays

##### Solubility Determination

Thermodynamic solubility at pH 7.4 was measured using an adapted miniaturized shake-flask method in a 96-well plate format.^69,70^ Briefly, 4 μL of a 10 mM stock in DMSO was added to a 96-well plate and evaporated using a GeneVac system. Phosphate-buffered saline at pH 7.4 was then added to the wells, and the plate was incubated for 24 h at 25 °C with shaking. At the end of the incubation, the samples were centrifuged at 3500*g* for 15 min and then transferred to an analysis plate. A calibration curve in DMSO for each sample, ranging from 10 to 220 μM, was prepared and included on the analysis plate. Analysis was then performed by HPLC-UV (Agilent 1200 rapid resolution HPLC coupled to a Diode Array Detector), and the solubility of each sample was determined from the corresponding calibration curve.

##### In Vitro Microsomal Stability

Metabolic stability was assessed using a single-point assay design in human (mixed gender), rat (male IGS), and mouse (male CD1) liver microsomes.^71^ Briefly, the compounds were incubated at 1 μM in human (mixed gender, XenoTech), rat (male rat IGS, XenoTech), and mouse (male mouse CD1, XenoTech) liver microsomes (0.4 mg/mL) and 1 mM NADPH for 30 min at 37 °C. Reactions were quenched by adding ice-cold acetonitrile containing internal standard. The samples were then centrifuged. Afterward, the samples were analyzed via LC-MS/MS to assess the disappearance of the parent compound. Half-life, clearance, and hepatic excretion ratios were determined using standard equations.^71,72^ The following equations were used to calculate the half-life (*t*_1/2_) and intrinsic clearance (Cl_int_):

$$t_{1/2}=\frac{0.693}{k}$$ 1

$$Cl_{int}={\frac{k}{\left[\mathrm{Protein}\right]}}^{*}{\frac{V_{incubation}}{W_{liver}}}^{*}SF$$ 2

where *k* is the elimination rate constant determined from the slope of ln­[concentration] vs time.

##### Human Plasma Protein Binding (hPPB)

The binding of derivatives to human plasma proteins was determined using an ultracentrifugation-based method.^70^ Acidic drugs tend to bind to albumin while basic drugs bind to α1-acid glycoproteins and lipoproteins instead.^70^ Briefly, the compounds were incubated at 1 μM in thawed plasma for 1 h at 37 °C. After incubation, samples were ultracentrifuged at 42,000 rpm for 4 h at 37 °C to separate plasma proteins from plasma water. Reactions were quenched by adding ice-cold acetonitrile containing internal standard. Afterward, the samples were analyzed by LC-MS/MS, and the % bound was determined. The fraction unbound (*F*_u_) was calculated as the ratio of the compound concentration in the buffer compartment to that in the plasma compartment. As showed below:

$$f_{u}=\frac{C_{buffer}}{C_{plasma}}$$ 3

where *C*_buffer_ and *C*_plasma_ represent the compound concentration in the buffer (aqueous) and plasma compartments, respectively.

##### In Vivo Mouse Pharmacokinetics

All studies and procedures were conducted with prior approval of the animal ethics committee of the University of Cape Town (approval number 021_002) by the South African National Standard (SANS 10386:008) for the Care and Use of Animals for Scientific Purposes,^73^ and guidelines from the Department of Health.^74^

Male Balb/C mice were bred at the University of Cape Town Research Animal Facility, Cape Town, South Africa. The compound was administered intravenously (3 mg/kg) to male Balb/C mice (*n* = 3) as a bolus of 10% (v/v) dimethyl sulfoxide (DMSO), 60% (v/v) propylene glycol, and 30% polyethylene glycol (PEG) 400. The oral dose (10 mg/kg) was administered to mice (*n* = 3) as an aqueous suspension containing 0.5% (w/v) hydroxypropylmethylcellulose (HPMC) and 0.2% (v/v) Tween 80. Mice were not fasted overnight and were allowed to eat ad libitum. Animals were permitted access to water through ad libitum.

##### Sample Analysis

Blood samples were collected from mice’s tail veins into heparinized microcentrifugation tubes at predetermined time points and stored frozen (−80 °C) until analysis.

##### Bioanalytical Method

The concentration of the compound was determined by LC-MS/MS using an AB Sciex API5500 triple quadrupole instrument. Chromatographic separation was conducted using an Agilent 1200 series HPLC. Blood samples and calibration standards (prepared in drug-free whole blood of the relevant species) were prepared by protein precipitation with cold acetonitrile, followed by centrifugation and analysis of the supernatant. The analytical limit of quantitation (LOQ) was 2 ng/mL. Each study’s accuracy, precision, and recovery were within acceptable limits.

##### Data Processing

Pharmacokinetic parameters were calculated via noncompartmental analysis using PK Solutions 2.0 (Summit Research Services, Montrose, Colorado, USA) based on curve stripping methods.

### Computational

#### Generative Molecule Design

MegaSyn^50^ was used to design new molecules via generative design. Briefly, a long short-term memory (LSTM)-based model was pretrained on ∼2 million SMILES from ChEMBL, learning to assemble valid, drug-like molecules from SMILES alone. Once pretrained, this model was used in a MegaSyn loop to find new SARS-CoV-2 active molecules. For SARS-CoV-2 activity predictions, we used a previously described data set consisting of 506 molecules (72 actives, 434 inactives),^7^ from which we built eight classification models using Assay Central, including Adaboost, Naïve Bayes, *k*-Nearest Neighbors, Logistic Regression, Random Forest, XGBoost, and Support Vector Machines, using 5-fold nested cross-validation. We then selected the best classification model by AUC, Naïve Bayes, to drive the predictions of SARS-CoV-2 activities in MegaSyn.

The MegaSyn training loop is as follows: First, MegaSyn was trained on the UMF Tanimoto similarity to narrow the generative chemical space, a process called “priming” the model. Priming was performed 14 times, progressively narrowing the space toward UMF-like structures in each subsequent run. Every two epochs of priming the model, four ensemble models were cloned off the primed model, and each of the four ensemble models was trained using a maximum likelihood estimation (MLE) hill climbing algorithm. First, each ensemble model is queried to generate a set of novel compounds. Next, the generated molecules are scored with the SARS-CoV-2 model for activity and ranked by total score. The top 10% of scored compounds are used to train the model, and the process repeats. Eventually, each ensemble model finds its way into a narrow chemical space of SARS-CoV-2-predicted active molecules. After priming and ensemble model training, the top 200 compounds by score were kept and evaluated for synthesis.

#### Molecular Docking at the S2 Domain of SARS-CoV-2 Spike Protein

The three-dimensional structure of the SARS-CoV-2 spike protein (PDB ID 6VXX)^67^ was adopted in docking calculations. The protein structure was prepared using the Protein Preparation Wizard^75^ tool at pH 7.4, with hydrogen atoms added and the structure energy minimized, employed by the OPLS4 force field.^76^ Ligands were prepared using LigPrep^77^ at pH 7.4, which included rotamer correction based on Epik^78^ energy minimization, with 32 conformers generated. The receptor grid was prepared using the Receptor Grid Generation tool, with the grid box centered on residues F1062A, L1063A, H1064A, S1021, L1024A, and T1027A, identified as UMF’s allosteric binding site.^39^ The grid box dimensions were set to *x*, *y*, and *z* coordinates of *x* = 201.5 Å, *y* = 212.9 Å, *z* = 168.5 Å, and the size of 19 Å.

Molecular docking calculations were conducted on the Maestro platform using Glide software in extra-precision (XP) mode,^79,80^ treating the ligand as flexible while keeping the protein rigid. The PLIP server^81^ was employed to analyze protein–ligand interactions. Additionally, PyMOL software^82^ was used for visual inspection of docking poses and for rendering three-dimensional molecular images.

#### Structure–Activity Relationship Study

The structure–activity relationship (SAR) was focused into two distinct parts of the core scaffold, i.e., the molecule’s left-hand (R_1_, R_2_, and R_3_) and right-hand (R_4_ and R_5_) sides (see Figure 3). The left-hand-side modifications involved functionalizing the indole at R_1_ and R_3_. In contrast, the right-hand-side modifications focused on exploring alkyl substituents at R_4_ and aromatic substituents at R_5_. Building on previous studies,^52^ we aimed at conducting a SAR study on indole derivatives that did not bear a free hydroxyl group. To this end, the derivatization of 5-methoxyindole compounds was prioritized.

## Supplementary Material



## Data Availability

The data curation script utilized in this study is available at https://github.com/LabMolUFG/cheminformatics_pipeline.

## Notes

Detailed computational procedures, experimental details, materials and methods, chemical structures of the UMF analogues, and compound characterization; compounds antiviral activity against Delta and Omicron variant (XBB1.5 lineage) and cytotoxicity; 5-fold cross-validation statistics for SARS-CoV-2 models; compound’s SMILES (PDF)

## Abbreviations

6HB: six-helix bundle
AUC: area under the curve
ACE2: angiotensin-converting enzyme 2
CC_50_: 50% cytotoxic concentration
CI: confidence interval
CL_int,app_: microsomal intrinsic clearance, apparent
*C*_max_: maximum concentration
COVID-19: coronavirus disease
CT: computed tomography
DCM: dichloromethane
EC_50_: 50% effective concentration
*F*%: bioavailability
HRMS: high-resolution mass spectrometry
LiP-MS: limited proteolysis coupled with mass spectrometry
MCC: Matthews correlation coefficient
MedChem: Medicinal Chemistry
MLE: maximum likelihood estimation
ML: machine learning
MOI: multiplicity of infection
mouse CLs: mouse systemic clearance
mouse PK: mouse pharmacokinetics
M^pro^: main protease
MS CL_int,app_: microsomal intrinsic clearance, apparent
NR-PCR: negative rate of polymerase chain reaction
PCR: polymerase chain reaction
PFU/mL: plaque-forming units per milliliter
PL^pro^: papain-like protease
RdRp: RNA-dependent RNA polymerase
SAR: structure–activity relationship
SARS-CoV-2: severe acute respiratory syndrome coronavirus 2
SI: selectivity index
Sol. @ pH 7.4: solubility at pH 7.4
S protein: spike protein
TSA: thermal shift assay
UMF: umifenovir
*V*_ss_: volume of distribution at steady state
