# Supplementary material for: Enhanced Antiviral Activity of Novel Umifenovir Derivatives against SARS-CoV-2: Insights from an International Collaborative Study
Source: ACS Omega. 2026 Apr 6;11(15):23109–24. doi: 10.1021/acsomega.5c13080 (PMC13103833; doi:10.1021/acsomega.5c13080)
Supplement: Supplementary file 1 [file ao5c13080_si_001.pdf]

Supplementary Information

Enhanced Antiviral Activity of Novel Umifenovir Derivatives Against SARS-CoV-2: Insights from an International Collaborative Study

Melina Mottin[1,2§], Christopher D. Jurisch[3§], Sabrina Silva-Mendonça[1,2§], Donald Seanego[3], Paulo R. P. da Silva Ramos[1,2], Caroline S. Freitas[4,5], Mayara Mattos[4,5], Natalia Fintelman-Rodrigues[4,5], Carolina Q. Sacramento[4,5], Florence Guivel-Benhassine[6], Timothée Bruel[6], Liezl Krugmann[3], Ana C. Puhl[7], Fabio Urbina[7], Thomas R. Lane[7], Eric M. Merten[8], Kenneth H. Pearce[8], Alexander Lepioshkin[9], Artem Poromov[9], Natalia Monakhova[9], Olivier Schwartz[6], Kelly Chibale[3], Thiago M. L. Souza[4,5], Sean Ekins[7], Vadim Makarov[9], Richard K. Gessner[3], Carolina H. Andrade[1,2,10*]

1. Center for the Research and Advancement in Fragments and Molecular Targets (CRAFT), Faculdade de Ciências Farmaceuticas de Ribeirão Preto, Universidade de São Paulo, Ribeirão Preto, SP, Brazil.
2. Laboratory for Molecular Modeling and Drug Design (LabMol), Faculdade de Farmácia, Universidade Federal de Goiás, Goiânia, GO, Brazil.
3. Holistic Drug Discovery and Development Centre (H3D), University of Cape Town (UCT), South Africa.
4. Laboratory of Immunopharmacology, Oswaldo Cruz Institute (IOC), Oswaldo Cruz Foundation (Fiocruz), Rio de Janeiro, RJ, Brazil.
5. National Institute for Science and Technology on Innovation in Diseases of Neglected Populations (INCT/IDPN), Center for Technological Development in Health (CDTS), Fiocruz, Rio de Janeiro, RJ, Brazil.
6. Virus and Immunity Unit, Institut Pasteur, Université Paris Cité, 28 rue du Dr Roux, Paris Cedex 15 75724, France.
7. Collaborations Pharmaceuticals, Inc, 840 Main Campus Drive, Lab 3510, Raleigh, NC 27606, USA.
8. Center for Integrative Chemical Biology and Drug Discovery, Chemical Biology and Medicinal Chemistry, Eshelman School of Pharmacy, University of North Carolina, Chapel Hill, North Carolina 27599, United States
9. Research Center of Biotechnology RAS, 33-1 Leninsky prospect, 119071, Moscow, Russia.

10. Center for Excellence in Artificial Intelligence (CEIA), Instituto de Informática, Universidade Federal de Goiás, Goiânia, GO, Brazil.

§ M. Mottin, C.D. Jurisch, and S. Silva-Mendonça contributed equally to this work.

*Corresponding author: Prof. Carolina Horta Andrade. Laboratory for Molecular Modeling and Drug Design (LabMol), Faculdade de Farmacia, Universidade Federal de Goias, 74605-170, Goiânia, GO, Brazil. Email: carolina@ufg.br

Table of contents

1. Supplementary Figures
Spike - RBD assays
SARS-CoV-2 replication in Calu-3 cells with different concentrations
Plasma Concentration–Time Profiles of Compound 11 in Mice After Intravenous and Oral Dosing
Plasma Concentration–Time Profiles of UMF in Mice After Intravenous and Oral Dosing
Evaluation of Virucidal Activity of Compounds 11, 18, UMF, and RDV Against SARS-CoV-2
Chemical Synthesis - Mannich reaction (Methyl derivatives)
Chemical Synthesis - Mannich reaction for Cyclopropyl and Cyclopropyl Methane derivatives - Method A
Vilsmeier-Haack Formylation – Method B
Reductive Amination – Method B
2. Supplementary Tables
Antiviral Assays Against Delta and Omicron (XBB.1.5 Lineage) Variants
SARS-CoV-2 Machine Learning Model Cross-validation – used in MegaSyn
3. Supplementary Schemes
Chemical Synthesis - Materials and Methods for Chemical Synthesis Performed at the Research Center of Biotechnology (RAS).
Synthesis and characterization of compounds with R1, R2, and R3 modifications
Synthesis and characterization of compounds with R4 and R5 modifications
Synthesis of Enaminones
Nenitzescu Indolisation
Methylation Procedure
Radical Chlorination Reaction
Synthesis of Substituted ((Phenylthio)methyl)indole Derivatives
Suzuki-Miyaura Coupling Reaction
4. References

1. Supplementary Figures

Spike - RBD assays

To verify if **UMF** and **3** could disrupt the interaction between the RBD and ACE2, they were evaluated in a Lumit SARS-CoV-2 spike RBD: hACE2 Immunoassay[48] (Promega). Interestingly, **UMF** and **3** did not disrupt the RBD -ACE2 interaction (see Supplementary Material, **Figure S1**).

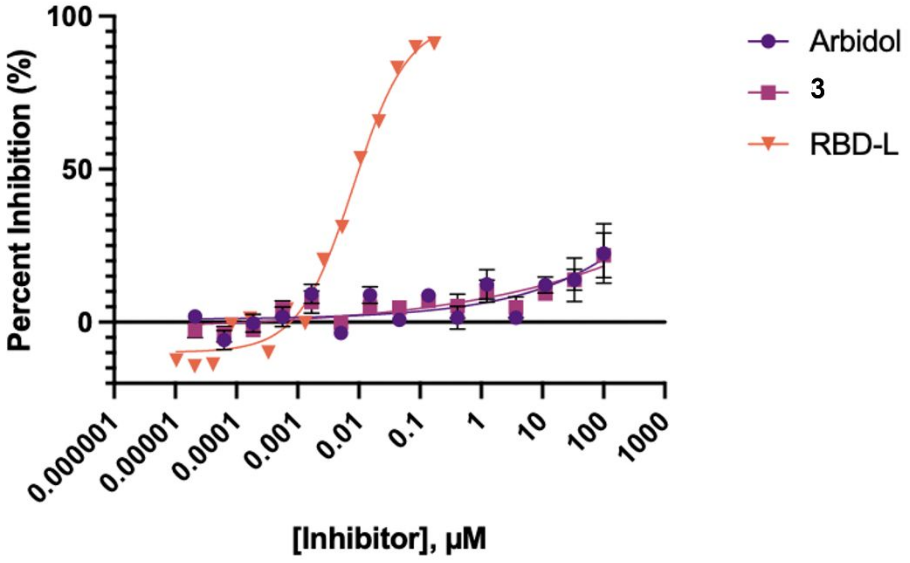

Figure S1. SARS-CoV 2 Spike RBD: hACE2 Immunoassay to evaluate a compounds potential to disrupt the interaction between spike RBD and ACE2[1].

SARS-CoV-2 replication in Calu-3 cells with different concentrations

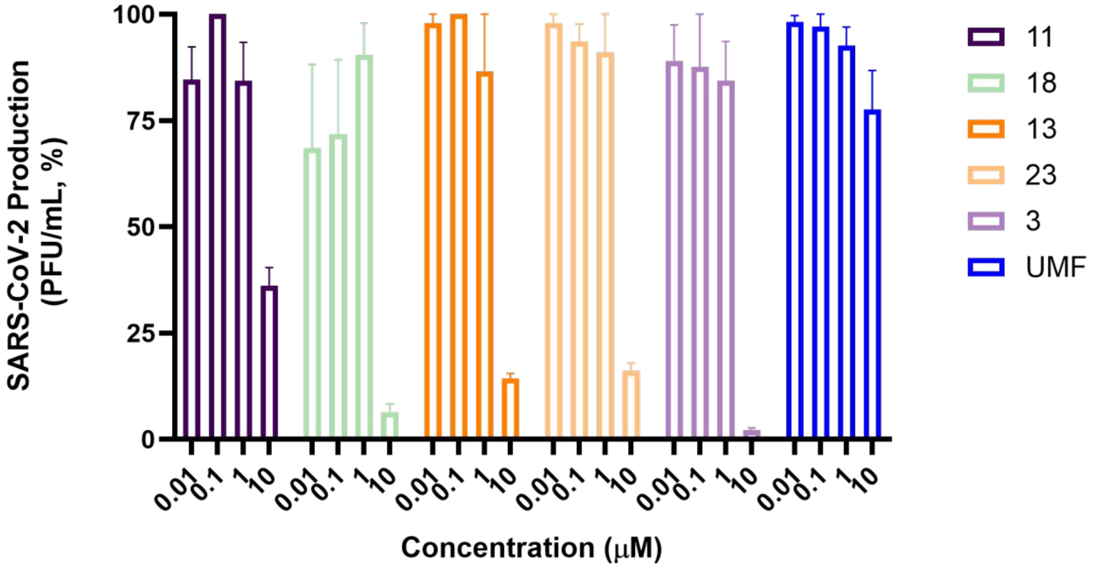

Figure S2. UMF analogs activity against SARS-CoV2 in Calu-3 cells (percentage of replication). Molecules that exhibited higher inhibition were tested in different concentrations (10, 1, 0.1 and 0.01 μM), enabling the calculation of EC_{50}. UMF was used as positive control.

Plasma Concentration–Time Profiles of Compound 11 in Mice After Intravenous and Oral Dosing

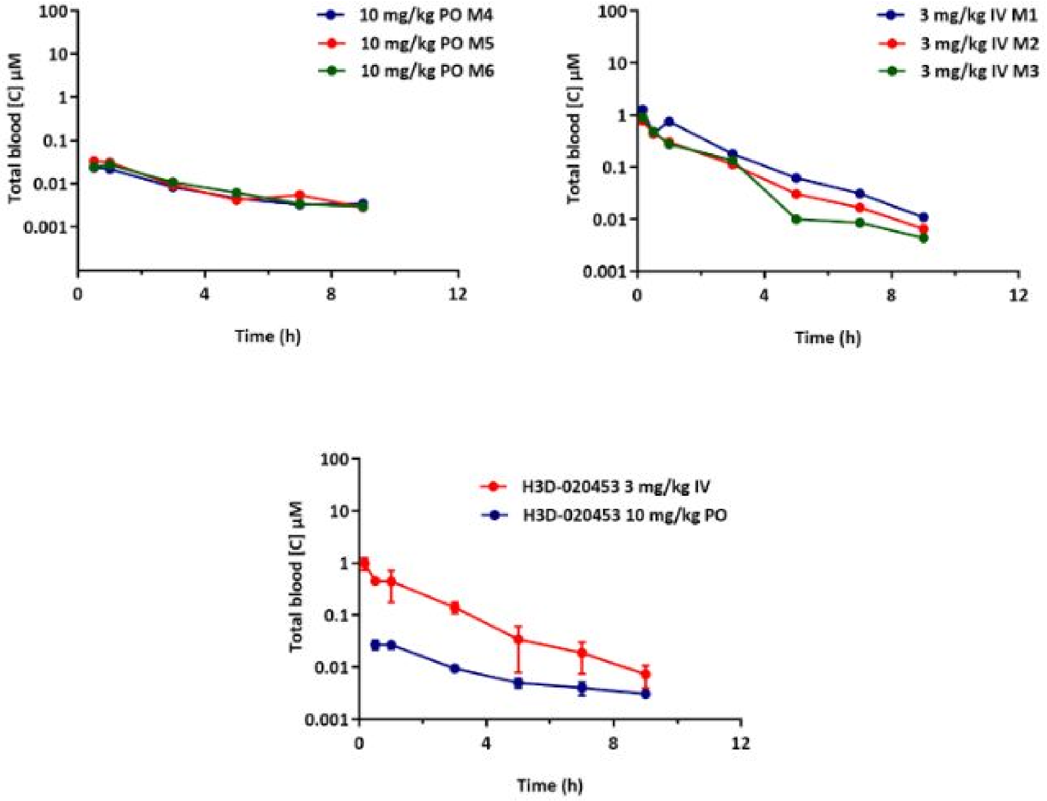

Figure S3. Plasma concentration–time profiles of compound 11 (H3D-020453) in mice following intravenous (3 mg/kg) and oral (10 mg/kg) administration. Individual data from three animals (M1–M3) are shown for each route, along with the mean ± SD in the bottom panel. Total blood

concentrations were measured at multiple time points up to 12 hours post-dose. Data are plotted on a logarithmic scale.

Plasma Concentration–Time Profiles of UMF in Mice After Intravenous and Oral Dosing

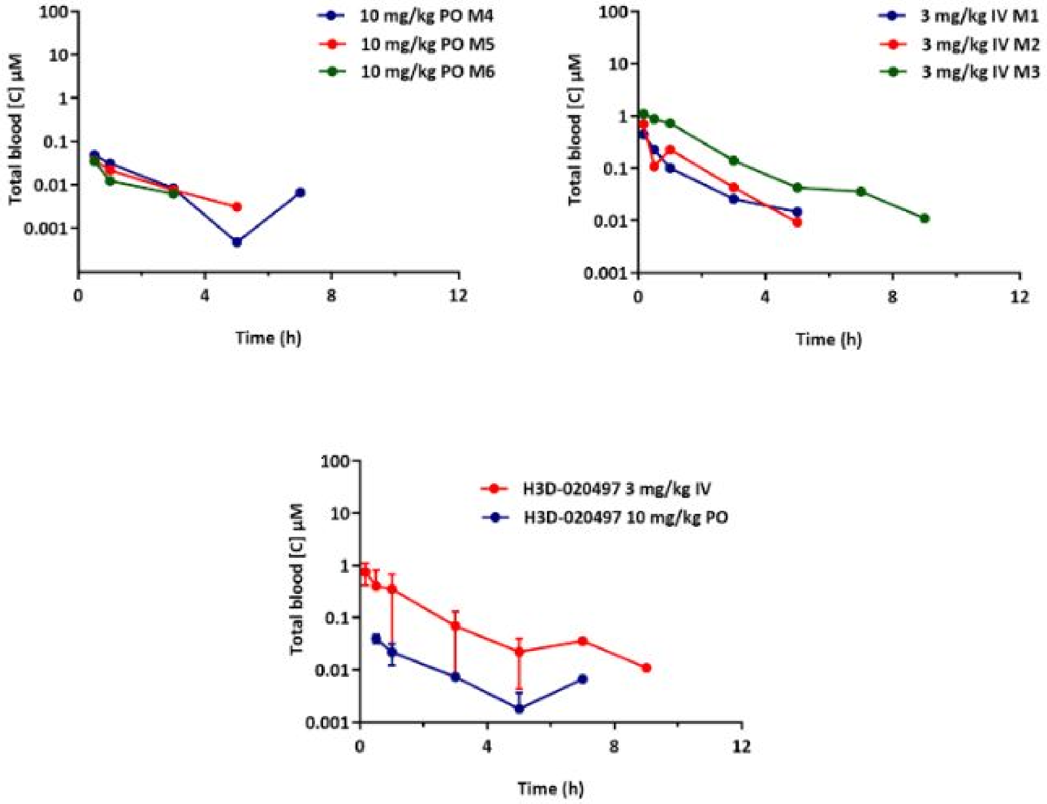

Figure S4. Plasma concentration–time profiles of UMF (H3D-020497) in mice following intravenous (3 mg/kg) and oral (10 mg/kg) administration. Individual data from three animals (M1–M3) are shown for each route, along with the mean ± SD in the bottom panel. Total blood

concentrations were measured at multiple time points up to 12 hours post-dose and plotted on a logarithmic scale.

Evaluation of Virucidal Activity of Compounds 11, 18, UMF, and RDV Against SARS-CoV-2

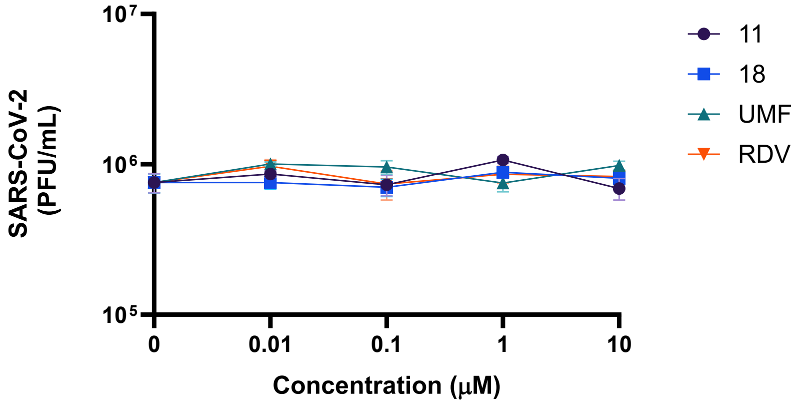

Figure S5. Virucidal assay. SARS-CoV-2 was incubated with compounds 11, 18, UMF, and RDV at concentrations of 0.01, 0.1, 1, and 10 μM for 1 hour at 37 °C. The virus–compound mixtures were then added to Calu-3 cells and incubated for an additional hour at 37 °C. After removal of the inoculum, fresh medium was added, and supernatants were collected 48 hours post-infection for viral titration by plaque assay.

Chemical Synthesis - Mannich reaction (Methyl derivatives)

N,N,N',N'-Tetramethyldiaminomethane (6.1 equiv) was added slowly to the appropriate indole (1.0 equiv, 500 mg scale) in a sealable vial under nitrogen at 0 °C. Glacial acetic acid (5.0 mL) was then added carefully to the vial. The vial was sealed and removed from the ice bath before being heated at 140 °C for 2 h. At this time, LC-MS analysis indicated the presence of primarily starting material and a trace amount of both Mannich regioisomers. After cooling to room temperature, excess acetic acid was removed under reduced pressure, and the crude product was purified via reverse-phase flash column chromatography using a Teledyne ISCO CombiFlash system eluting a gradient of MeCN + 0.1% formic acid in H_2O + 0.1% formic acid (typically 10 – 100%). Unavoidable co-elution with the unwanted regioisomer occurred during purification. Co-eluted fractions were lyophilized overnight and repurified using a Teledyne ISCO ACCQPrep HP 150 system eluting a gradient of MeCN + 0.1% formic acid in H_2O + 0.1% formic acid (typically 10 – 60%). Once dried via lyophilization, the product was

dissolved in 20% MeOH:DCM and loaded onto a 2 g SCX column (propylsulfonic acid). The column was washed with 20% MeOH:DCM, and the product was eluted with 3.5 M NH_3 in MeOH before being lyophilized again to afford the product as the freebase (2 – 8% yield).

Compound 11 - Ethyl 6-((dimethylamino)methyl)-5-methoxy-1-methyl-2-((phenylthio)methyl)-1*H*-indole-3-carboxylate – white solid, 3% yield.

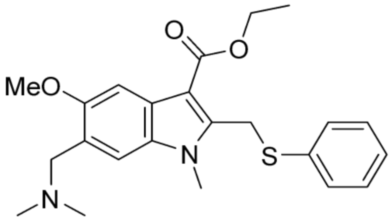

Figure S6. LC-MS ESI$^+$. t_R = 0.825 min (purity 100%); exact mass calcd for $C_{23}H_{28}N_2O_3S$: 412.18, found: *m/z* 413.2 $[M + H]^+$.

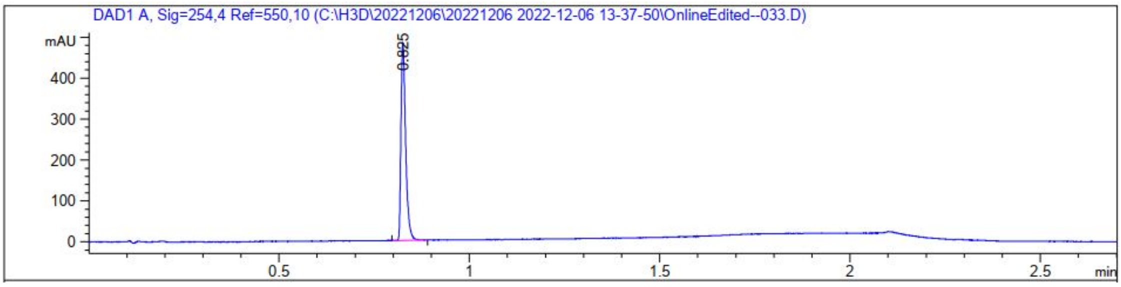


```
Signal 1: DAD1 A, Sig=254,4 Ref=550,10

Peak RetTime Type  Width     Area      Height     Area
  #   [min]        [min]   [mAU*s]     [mAU]       %
----|-------|----|-------|----------|----------|--------|
   1   0.825 BB    0.0131  417.59711  480.30399 100.0000

Totals :                   417.59711  480.30399
```

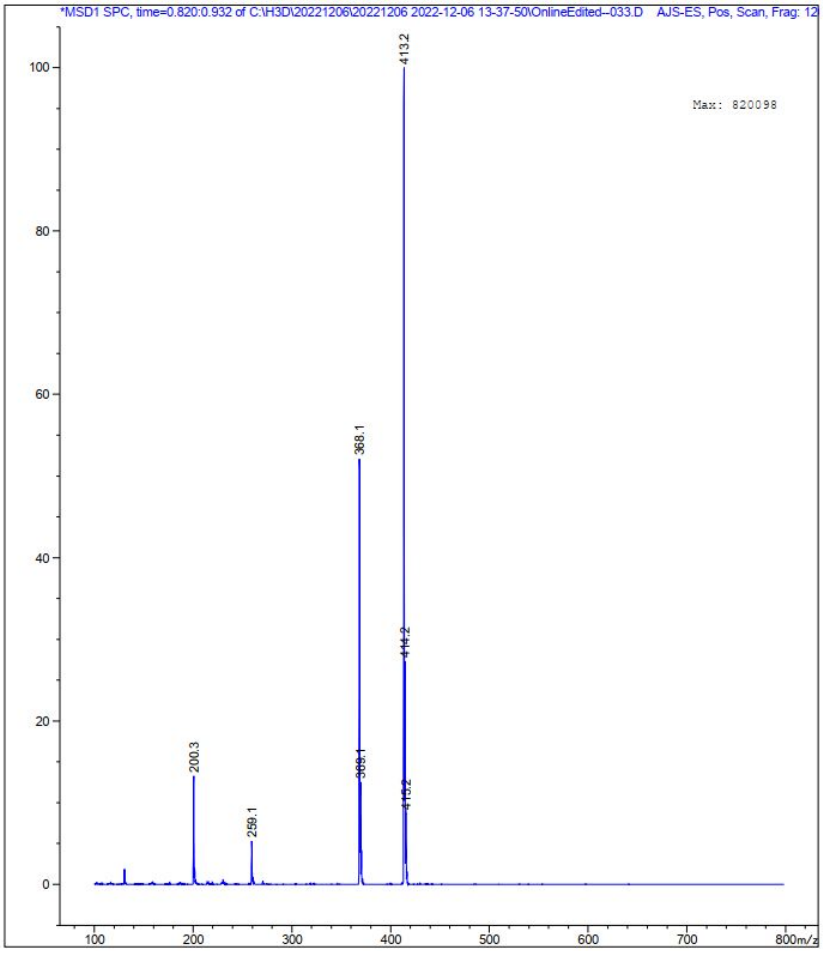

Figure S7. ^{1}H NMR (300 MHz, $CDCl_3$) δ 7.59 (s, 1H), 7.35 (s, 1H), 7.33 – 7.28 (m, 2H), 7.26 – 7.20 (m, 3H), 4.72 (s, 2H), 4.22 (q, 2H, *J* = 7.1 Hz), 3.88 (s, 3H), 3.74 (s, 2H), 3.68 (s, 3H), 2.37 (s, 6H), 1.36 (t, 3H, *J* = 7.1 Hz).

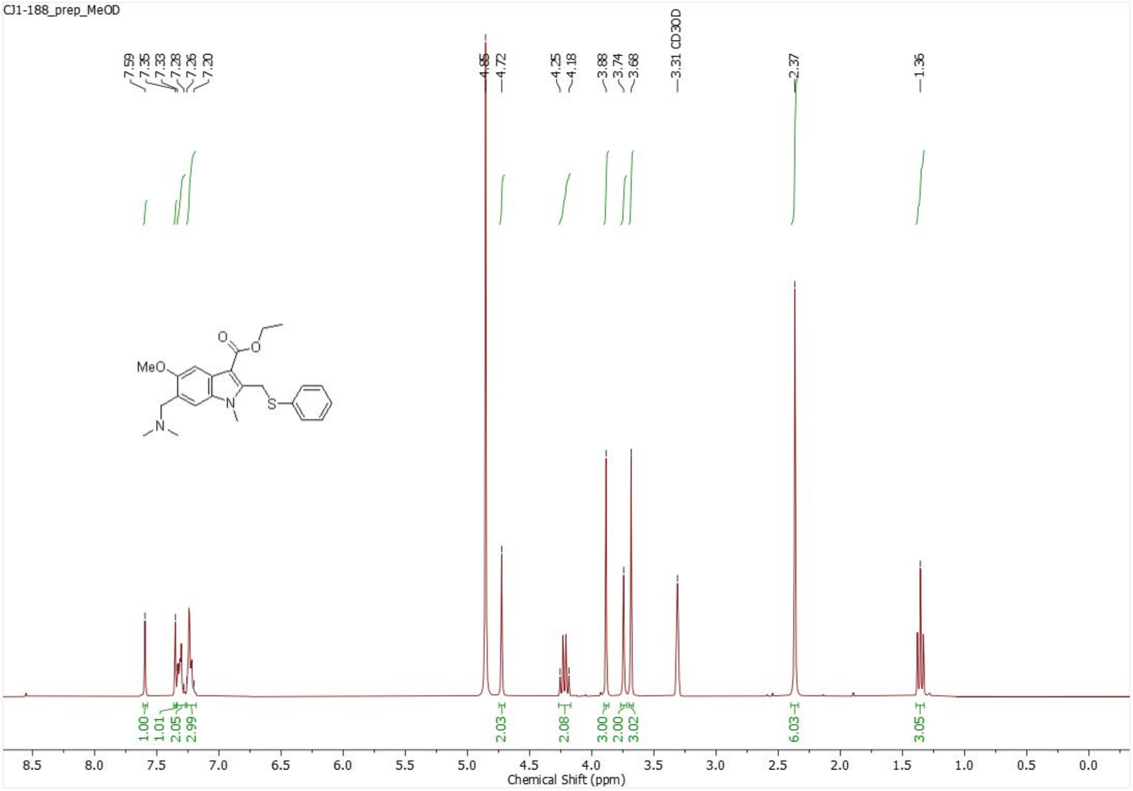

Compound 18 - Ethyl 2-(((4-bromophenyl)thio)methyl)-6-((dimethylamino)methyl)-5-methoxy-1-methyl-1*H*-indole-3-carboxylate – white solid, 3% yield.

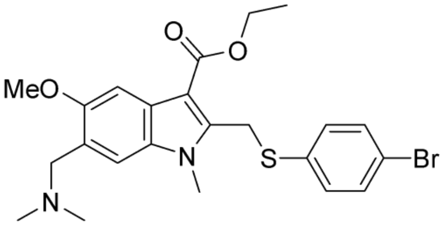

Figure S8. LC-MS ESI$^+$. t_R = 0.875 min (purity 100%); exact mass calcd for $C_{23}H_{27}BrN_2O_3S$: 490.09, found: *m/z* 491.1 $[M + H]^+$ (Br isotopic pattern observed).

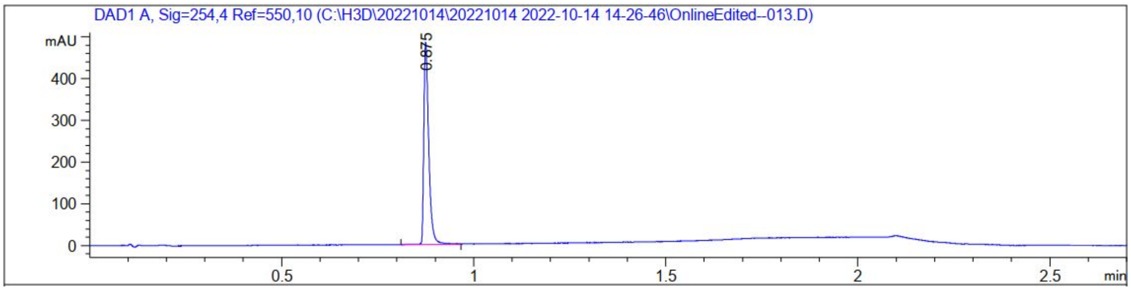

Signal 1: DAD1 A, Sig=254,4 Ref=550,10

Peak #	RetTime [min]	Type	Width [min]	Area [mAU*s]	Height [mAU]	Area %
1	0.875	BB	0.0135	430.58295	480.52533	100.0000

Totals :	430.58295	480.52533

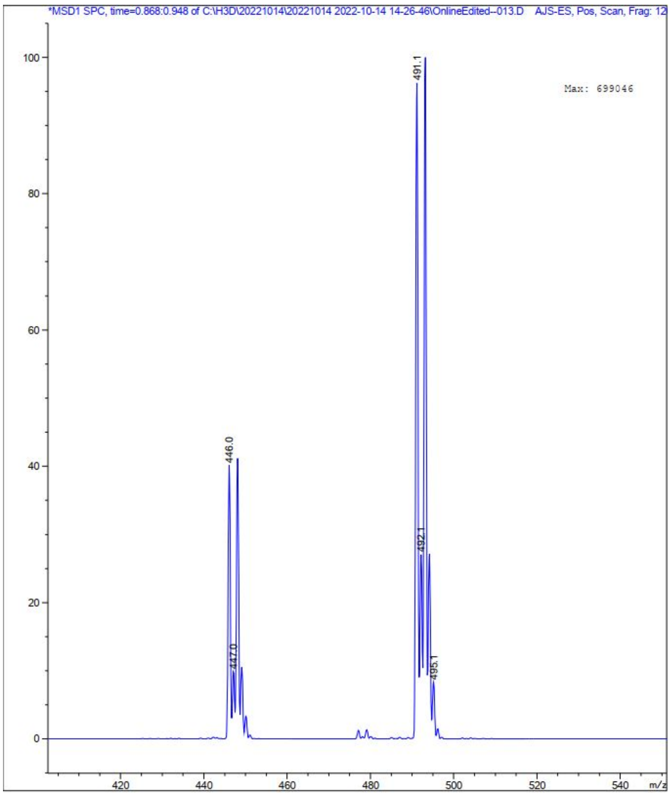

Figure S9. ^{1}H NMR (300 MHz, MeOD) δ 7.59 (s, 1H), 7.40 (s, 1H), 7.36 (d, 2H, *J* = 8.3 Hz), 7.19 (d, 2H, *J* = 8.3 Hz), 4.74 (s, 2H), 4.21 (q, 2H, *J* = 7.1 Hz), 3.90 (s, 3H), 3.84 (s, 2H), 3.74 (s, 3H), 2.43 (s, 6H), 1.36 (t, 3H, *J* = 7.1 Hz)

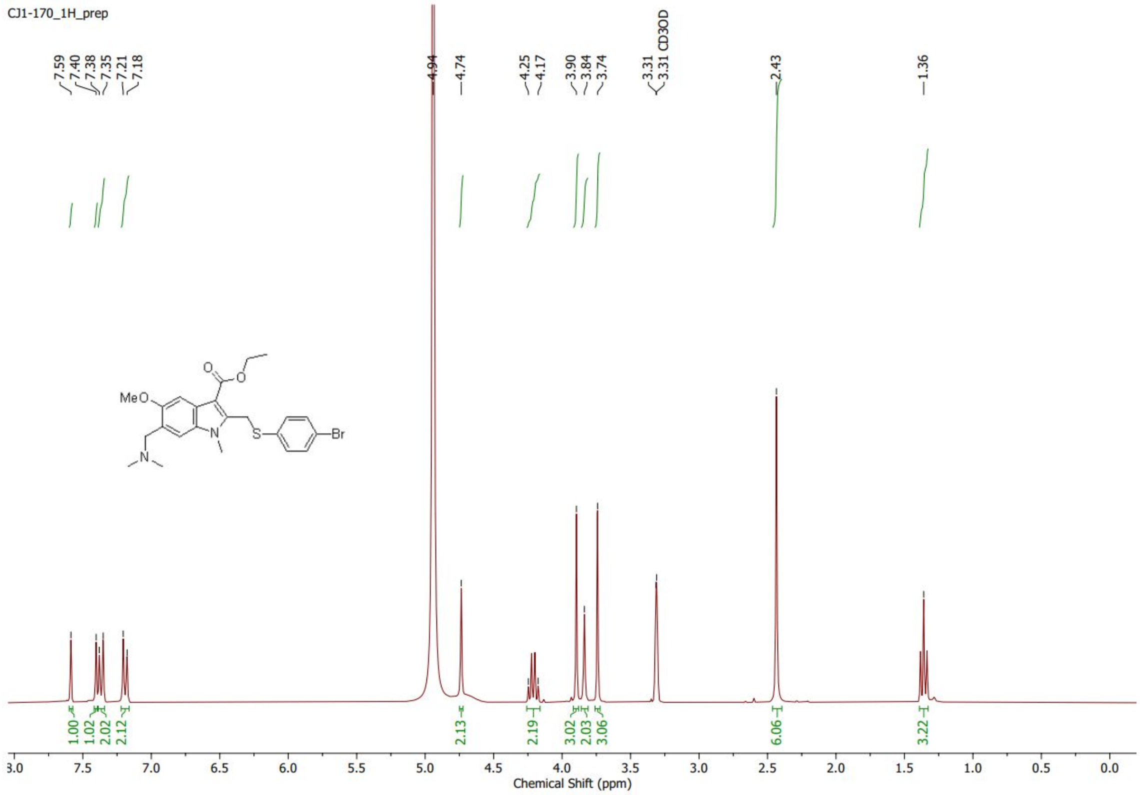

Compound 21 - Ethyl 6-((dimethylamino)methyl)-2-(((4-fluorophenyl)thio)methyl)-5-methoxy-1-methyl-1*H*-indole-3-carboxylate – off-white solid, 5% yield

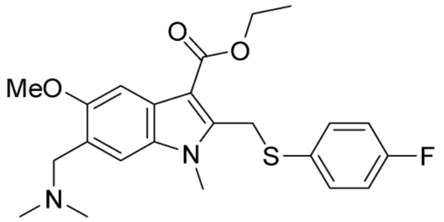

Figure S10. LC-MS ESI$^+$. t_R = 0.825 min (purity 100%); exact mass calcd for $C_{23}H_{27}FN_2O_3S$: 430.17, found: *m/z* 431.2 $[M + H]^+$.

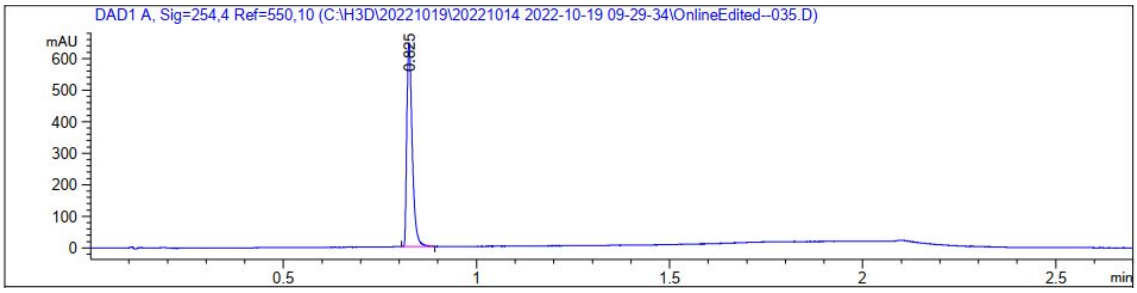

Signal 1: DAD1 A, Sig=254,4 Ref=550,10

Peak #	RetTime [min]	Type	Width [min]	Area [mAU*s]	Height [mAU]	Area %
1	0.825	BB	0.0153	627.64886	645.74609	100.0000

Totals : 627.64886 645.74609

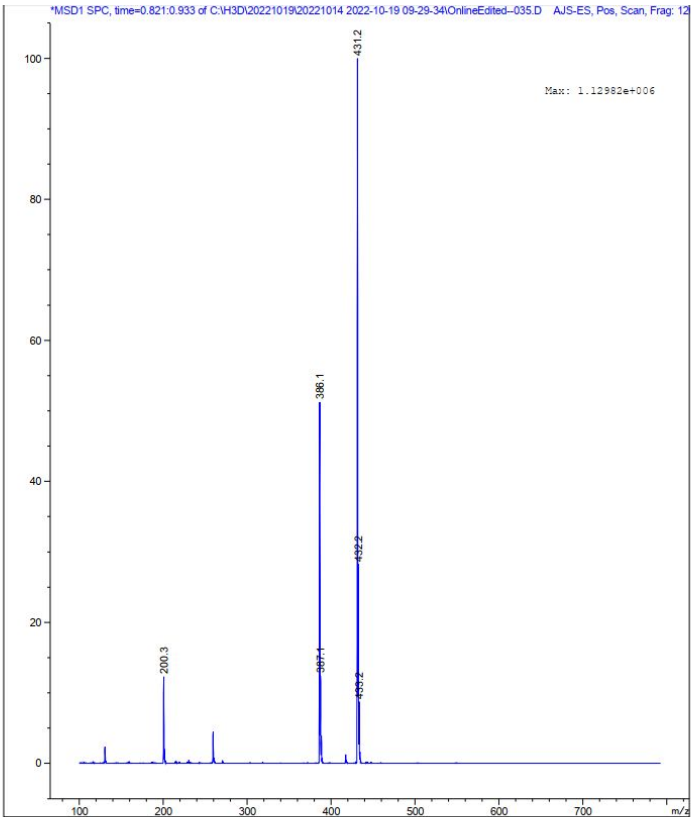

Figure S11. ^{1}H NMR (300 MHz, MeOD) δ 7.58 (s, 1H), 7.40 (s, 1H), 7.31 – 7.26 (m, 2H)*, 6.97 – 6.91 (m, 2H)*, 4.68 (s, 2H), 4.19 (q, 2H, *J* = 7.1 Hz), 3.89 (s, 3H), 3.87 (s, 2H), 3.71 (s, 3H), 2.46 (s, 6H), 1.35 (t, 3H, *J* = 7.1 Hz).

* These two signals have been reported as multiplets as the resolution of the ^{1}H NMR spectroscopic data was not high enough to accurately obtain *J*-coupling values. The more upfield signal appears to be a triplet but is actually two overlapping doublets that are poorly defined.

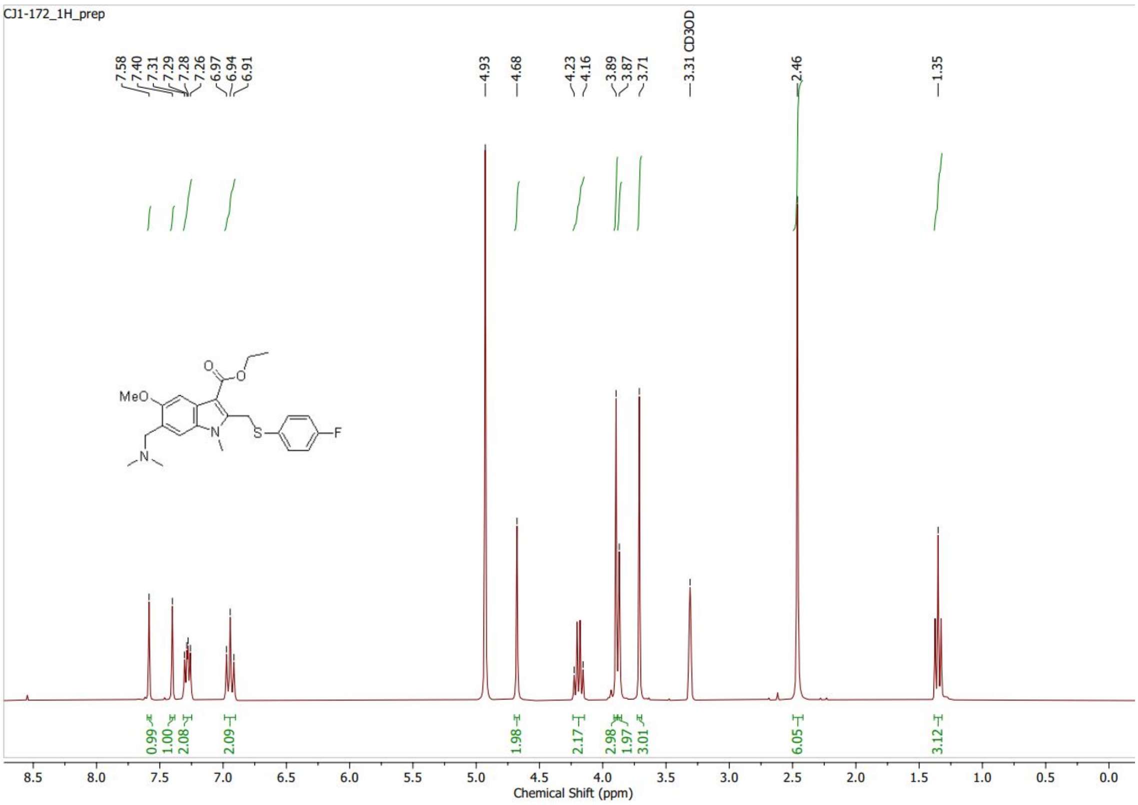

Compound 16 - Ethyl 2-(((4-chlorophenyl)thio)methyl)-6-((dimethylamino)methyl)-5-methoxy-1-methyl-1*H*-indole-3-carboxylate – white solid, 4% yield

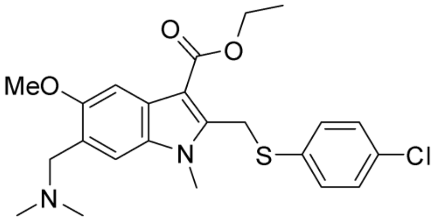

Figure S12. LC-MS ESI⁺. t_R = 0.871 min (purity 100%); exact mass calcd for $C_{23}H_{27}ClN_2O_3S$: 446.1, found: *m/z* 447.1 $[M + H]^+$ (Cl isotopic pattern observed).

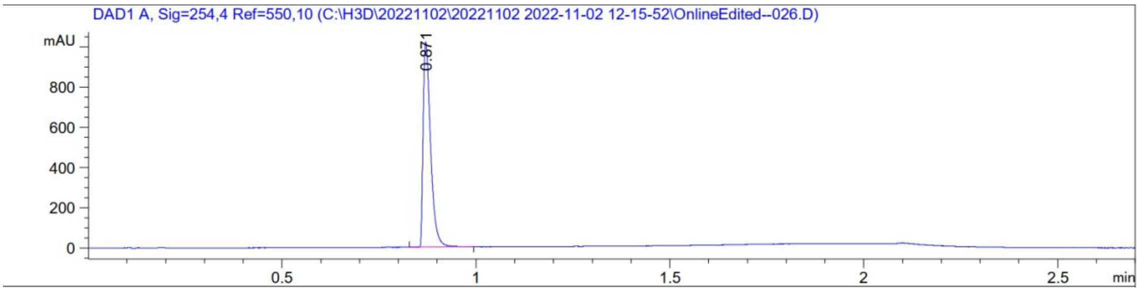

```
Signal 1: DAD1 A, Sig=254,4 Ref=550,10
```

Peak #	RetTime [min]	Type	Width [min]	Area [mAU*s]	Height [mAU]	Area %
1	0.871	BB	0.0205	1357.67029	1018.71045	100.0000
Totals :				1357.67029	1018.71045	

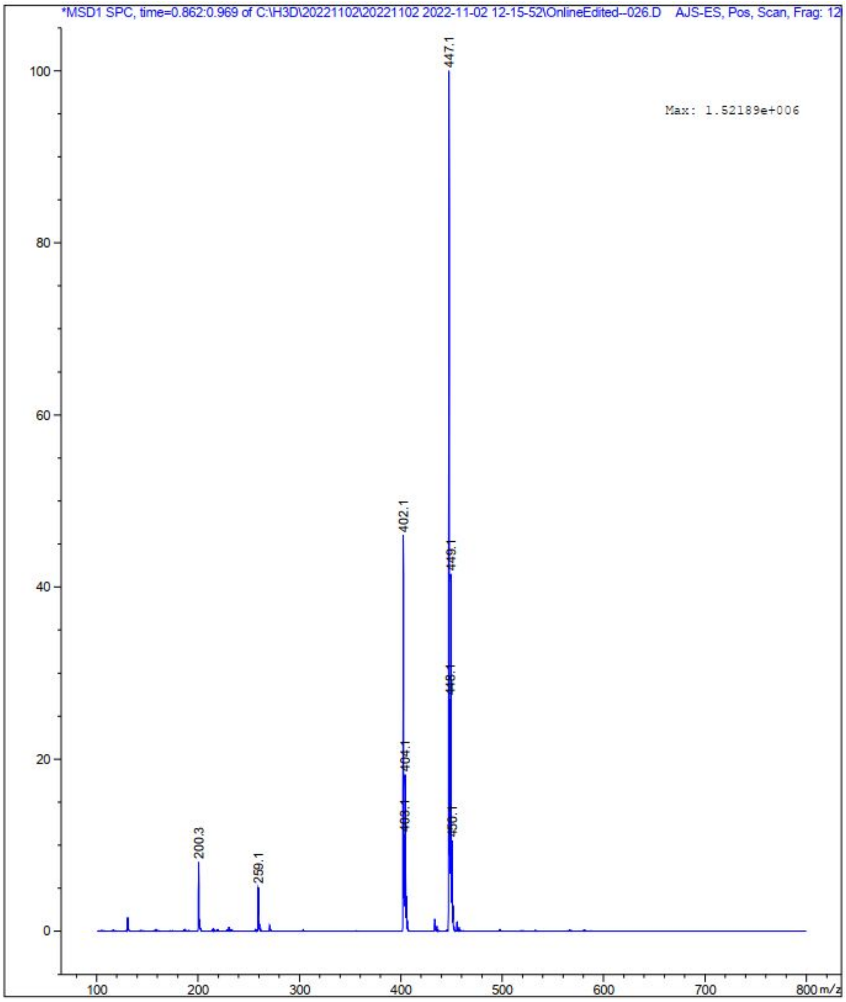

Figure S13. ^{1}H NMR (300 MHz, MeOD) δ 7.57 (s, 1H), 7.36 (s, 1H), 7.25 (d, 2H, J = 8.6 Hz),* 7.20 (d, 2H, J = 8.6 Hz),* 4.72 (s, 2H), 4.20 (q, 2H, J = 7.1 Hz), 3.88 (s, 3H), 3.72 (s, 5H),** 2.35 (s, 6H), 1.35 (t, 3H, J = 7.1 Hz).

* These two doublets are nearly overlapping and appear as an apparent quartet.

** The Mannich methylene moiety and methoxy CH_3 are overlapping to give rise to a singlet that integrates for 5 protons.

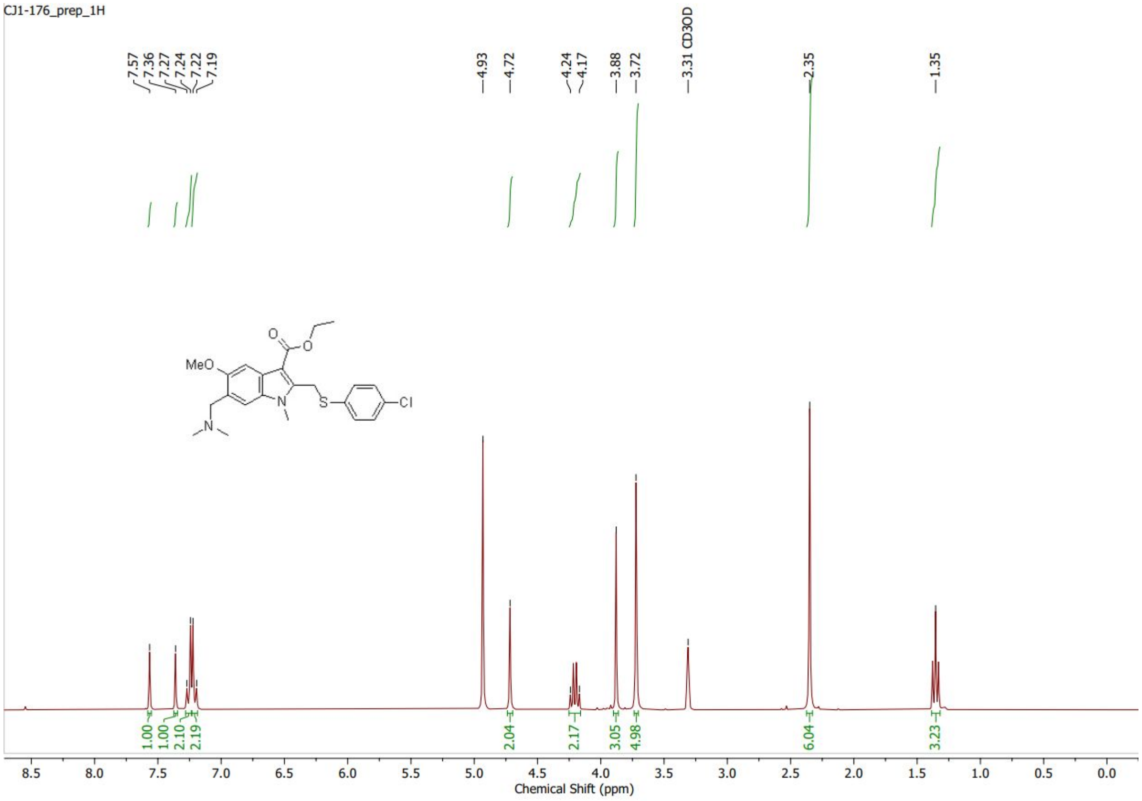

Compound 25 - Ethyl 6-((dimethylamino)methyl)-5-methoxy-1-methyl-2-((pyridin-2-ylthio)methyl)-1*H*-indole-3-carboxylate – light-brown gum, 4% yield

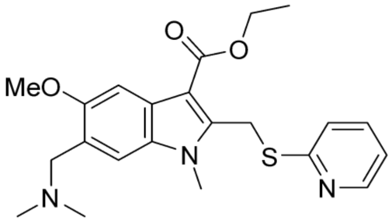

Figure S14. LC-MS ESI$^+$. t_R = 0.763 min (purity 100%); exact mass calcd for $C_{22}H_{27}N_3O_3S$: 413.18, found: *m/z* 414.2 $[M + H]^+$.

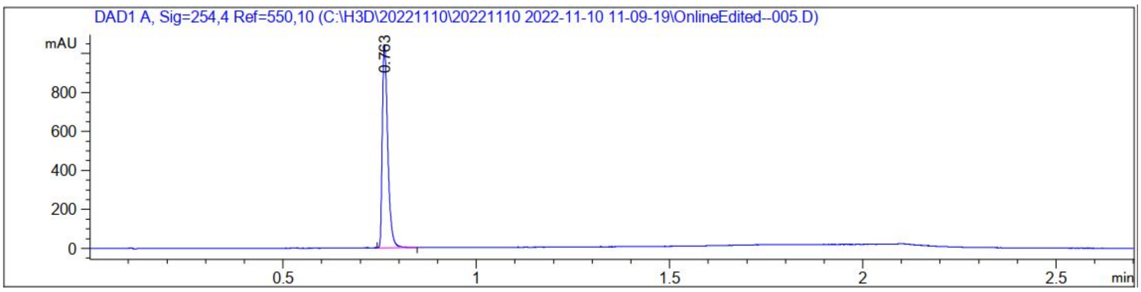


```
Signal 1: DAD1 A, Sig=254,4 Ref=550,10

Peak RetTime Type  Width     Area       Height     Area
  #   [min]        [min]   [mAU*s]     [mAU]       %
----|-------|----|-------|----------|----------|--------|
   1   0.763 BB    0.0151 1033.88586 1038.01929 100.0000

Totals :                  1033.88586 1038.01929
```

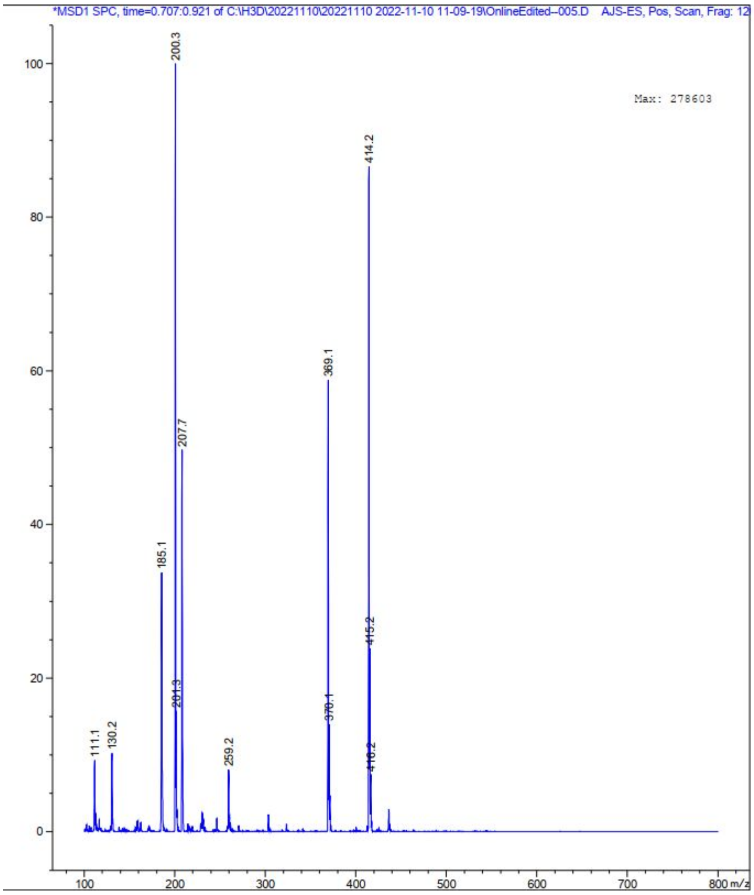

Figure S15. ^{1}H NMR (300 MHz, MeOD) δ 8.46 (d, 1H, *J* = 4.8 Hz), 7.62 - 7.56 (m, 2H), 7.34 (s, 1H), 7.26 (d, 1H, *J* = 8.1 Hz), 7.14 - 7.09 (m, 1H), 5.03 (s, 2H), 4.33 (q, 2H, *J* = 7.1 Hz), 3.89 (s, 3H), 3.77 (s, 3H), 3.75 (s, 2H), 2.37 (s, 6H), 1.39 (t, 3H, *J* = 7.1 Hz)

* Trace formate signal observed at δ 8.55 ppm despite freebasing the product via SCX. The product has been reported as the freebase.

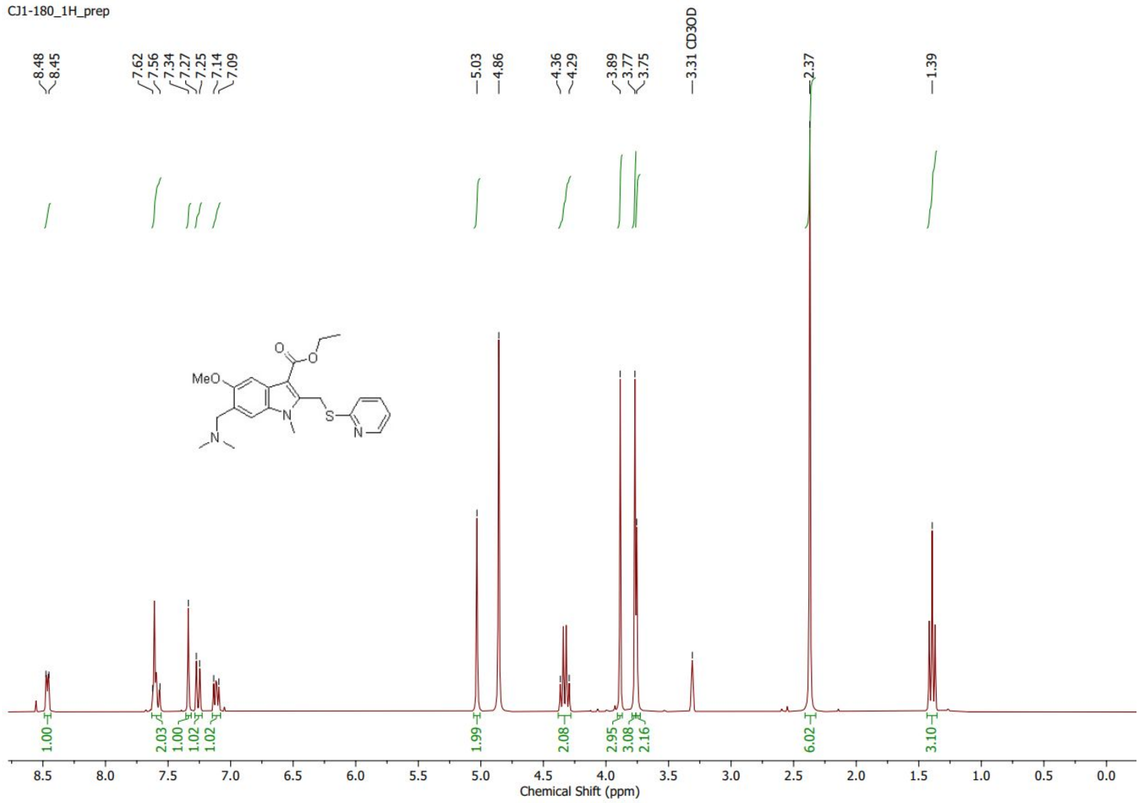

Compound 27 - Ethyl 6-((dimethylamino)methyl)-5-methoxy-1-methyl-2-((pyrimidin-2-ylthio)methyl)-1*H*-indole-3-carboxylate – off-white solid, 3% yield

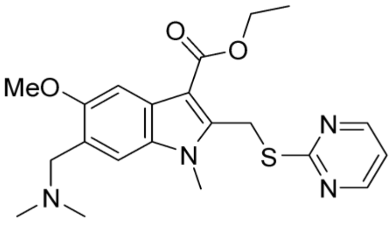

Figure S16. LC-MS ESI$^+$. t_R = 0.728 min (purity 100%); exact mass calcd for $C_{21}H_{26}N_4O_3S$: 414.17, found: *m/z* 415.2 $[M + H]^+$.

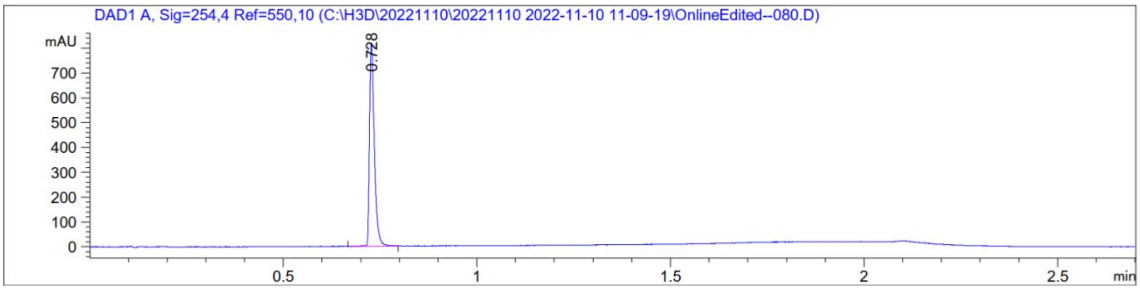


```
Signal 1: DAD1 A, Sig=254,4 Ref=550,10

Peak RetTime Type  Width     Area      Height     Area
  #   [min]        [min]   [mAU*s]     [mAU]        %
----|-------|----|-------|----------|----------|--------|
   1   0.728 BB    0.0136  708.56305  816.81952 100.0000

Totals :                   708.56305  816.81952
```

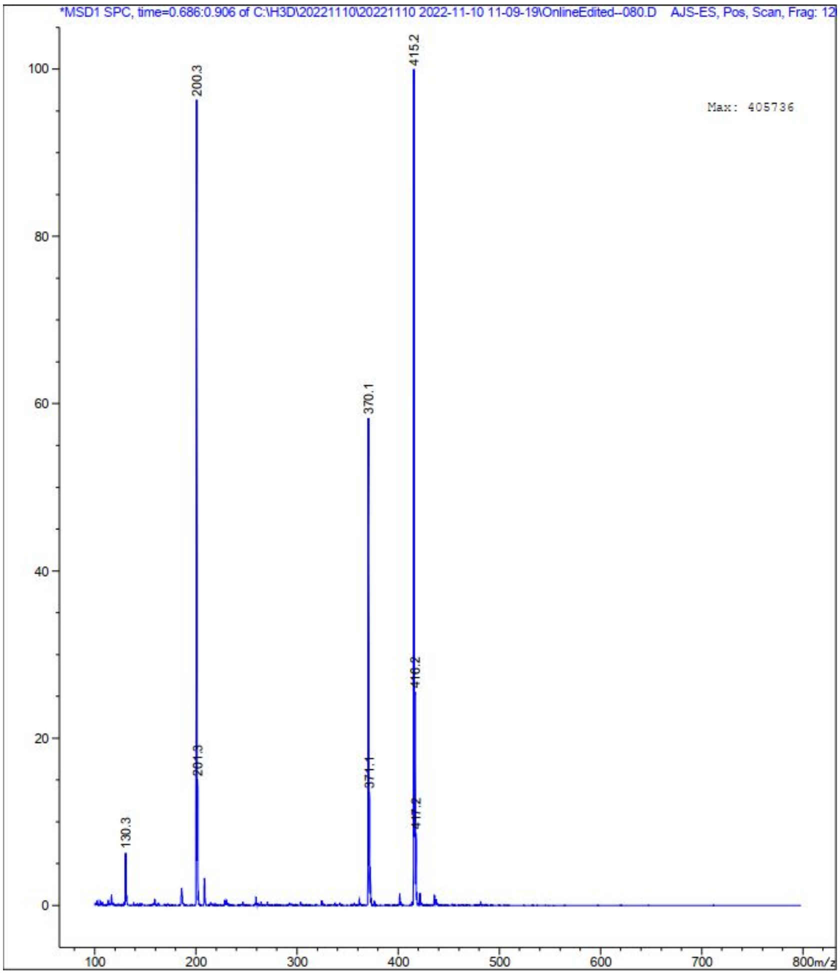

Figure S17. ^{1}H NMR (300 MHz, MeOD) δ 8.63 (d, 2H, *J* = 4.8 Hz), 7.66 (s, 1H), 7.42 (s, 1H), 7.18 (t, 1H, *J* = 4.9 Hz), 5.08 (s, 2H), 4.37 (q, 2H, *J* = 7.1 Hz), 3.92 (s, 5H),* 3.87 (s, 3H), 2.48 (s, 6H), 1.42 (t, 3H, *J* = 7.1 Hz).

* This singlet arises from the overlap of the signals produced by the Mannich methylene protons (2H) and the indole N-methyl protons (3H).

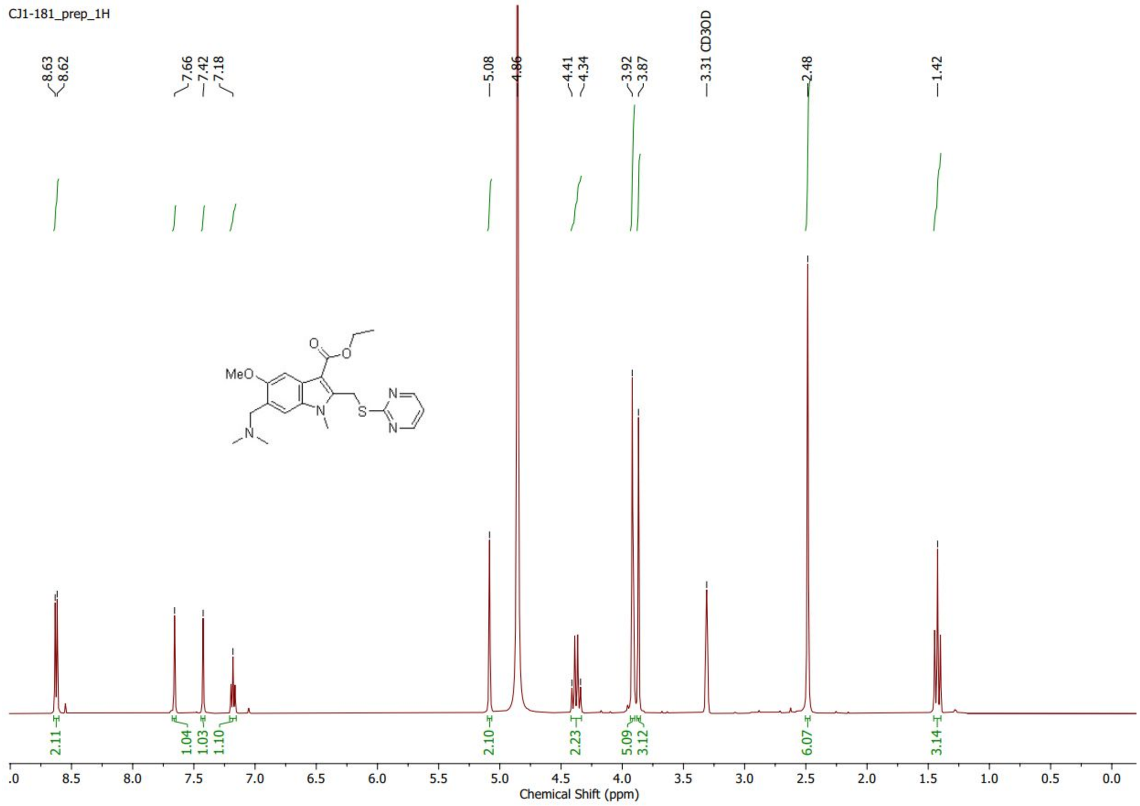

Compound 14 - Ethyl 6-((dimethylamino)methyl)-5-methoxy-1-methyl-2-((*p*-tolylthio)methyl)-1*H*-indole-3-carboxylate – white solid, 4% yield.

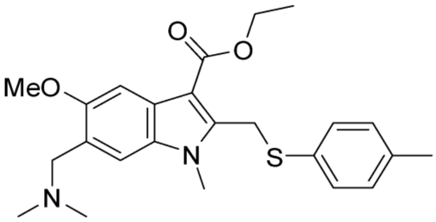

Figure S18. LC-MS ESI$^+$. t_R = 0.857 min (purity 100%); exact mass calcd for $C_{24}H_{30}N_2O_3S$: 426.20, found: *m/z* 427.2 [M + H]$^+$.

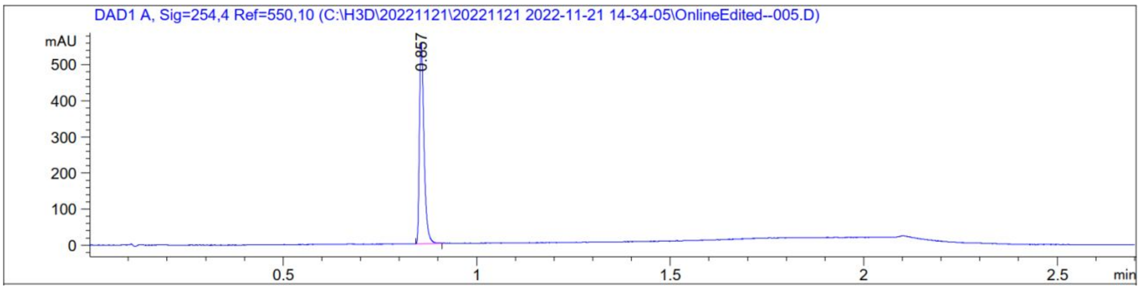

Signal 1: DAD1 A, Sig=254,4 Ref=550,10

Peak #	RetTime [min]	Type	Width [min]	Area [mAU*s]	Height [mAU]	Area %
1	0.857	BB	0.0128	465.48962	556.34753	100.0000
Totals :				465.48962	556.34753	

Figure S19. ^{1}H NMR (300 MHz, MeOD) δ 7.58 (s, 1H), 7.33 (s, 1H), 7.17 (d, 2H, J = 8.0 Hz), 7.03 (d, 2H, J = 8.0 Hz), 4.63 (s, 2H), 4.20 (q, 2H, J = 7.1 Hz), 3.88 (s, 3H), 3.73 (s, 2H), 3.64 (s, 3H), 2.36 (s, 6H), 2.28 (s, 3H), 1.35 (t, 3H, J = 7.1 Hz)

* Trace formate signal observed at δ 8.55 ppm despite freebasing the product via SCX. The product has been reported as the freebase.

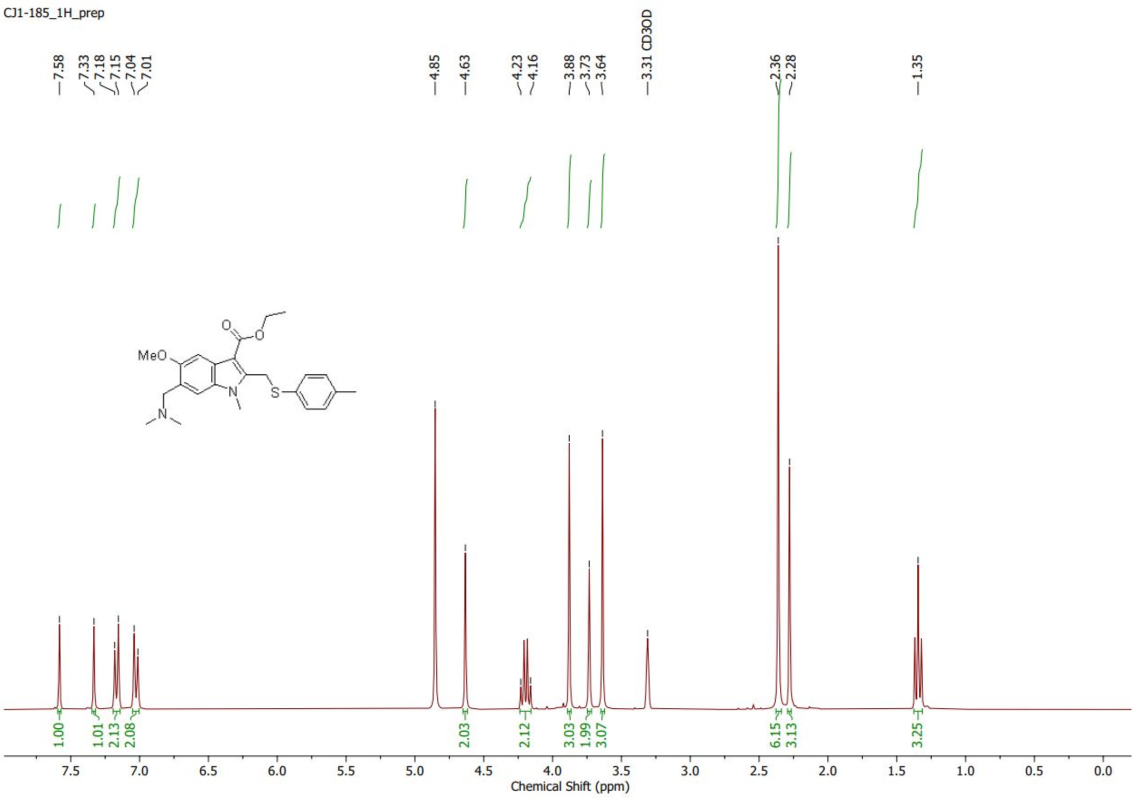

Compound 8 - Ethyl 2-((cyclohexylthio)methyl)-6-((dimethylamino)methyl)-5-methoxy-1-methyl-1*H*-indole-3-carboxylate – white solid, 3% yield.

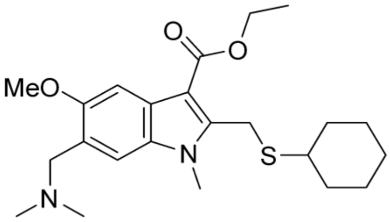

Figure S20. LC-MS ESI$^+$. t_R = 0.903 min (purity 100%); exact mass calcd for $C_{23}H_{34}N_2O_3S$: 418.23, found: m/z 419.2 $[M + H]^+$.

*Note that injection peak is visible on LC-MS data.

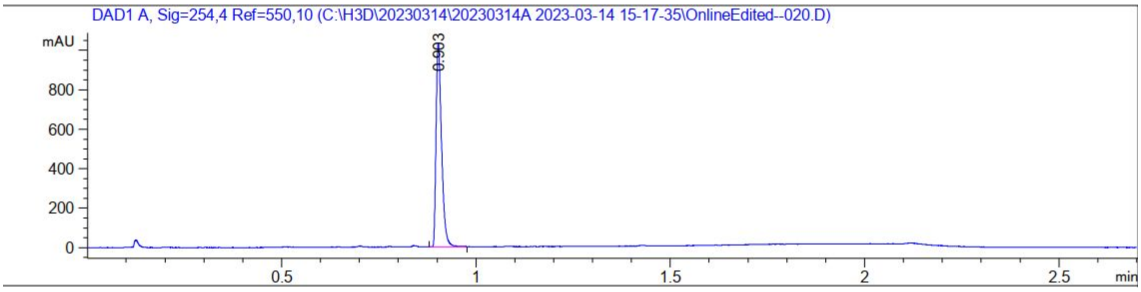

Signal 1: DAD1 A, Sig=254,4 Ref=550,10

Peak #	RetTime [min]	Type	Width [min]	Area [mAU*s]	Height [mAU]	Area %
1	0.903	BB	0.0150	1017.18298	1032.00806	100.0000
Totals :				1017.18298	1032.00806	

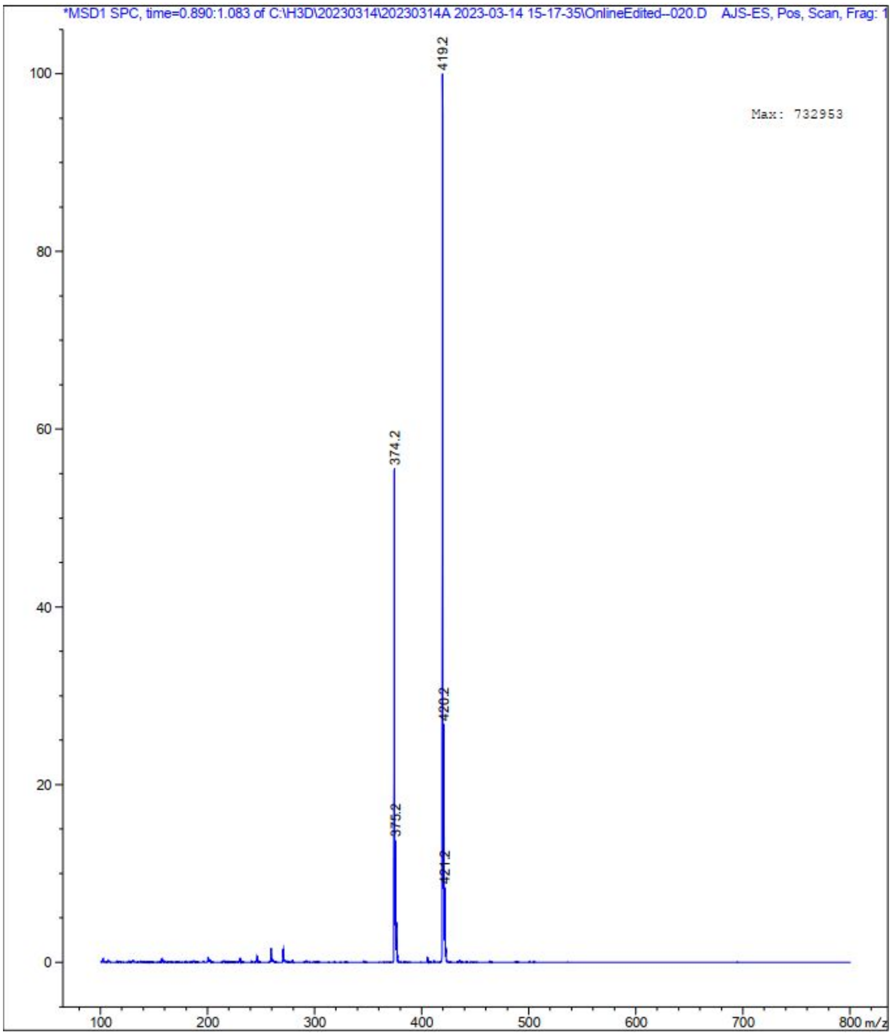

Figure S21. ^{1}H NMR (300 MHz, MeOD) δ 7.63 (s, 1H), 7.37 (s, 1H), 4.42 (s, 2H),* 4.37 (q, 2H, *J* = 7.1 Hz)**,*** 3.90 (s, 3H), 3.79 (s, 3H), 3.76 (s, 2H), 2.76 – 2.63 (m, 1H), 2.38 (s,

6H), 2.01 – 1.87 (m, 2H), 1.78 – 1.65 (m, 2H), 1.62 – 1.53 (m, 1H), 1.46 (t, 3H, J = 7.1 Hz), 1.36 – 1.18 (m, 5H).

* These signals overlap to produce a signal with a total integration of 4 protons (see expansion).

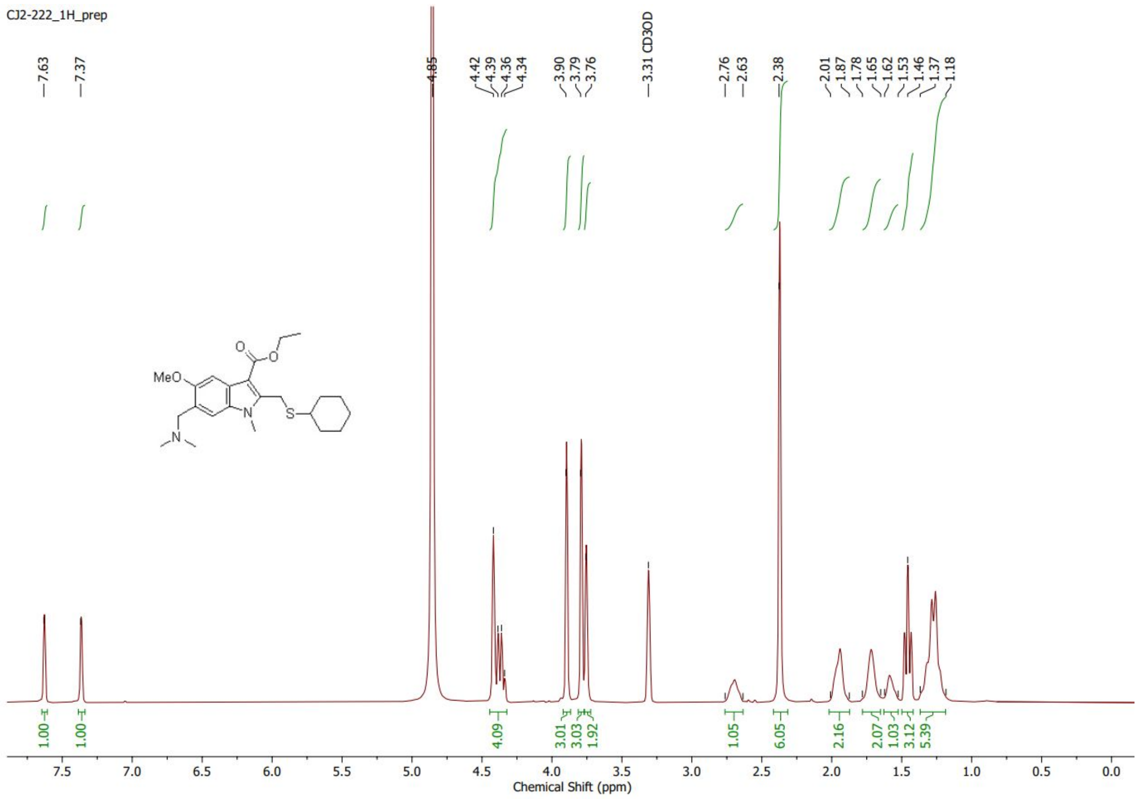

Compound 28 - Ethyl 6-((dimethylamino)methyl)-2-(((4'-fluoro-[1,1'-biphenyl]-4-yl)thio)methyl)-5-methoxy-1-methyl-1*H*-indole-3-carboxylate – white solid, 3% yield.

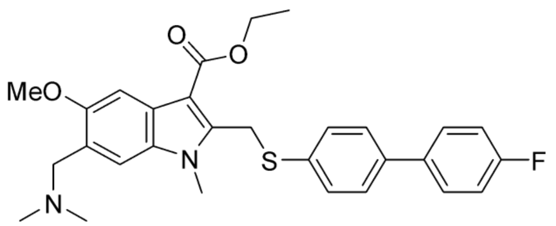

Figure S22. LC-MS ESI$^+$. t_R = 0.955 min (purity 100%); exact mass calcd for $C_{29}H_{31}FN_2O_3S$: 506.20, found: m/z 507.2 $[M + H]^+$.

*Note that injection peak is visible on LC-MS data.

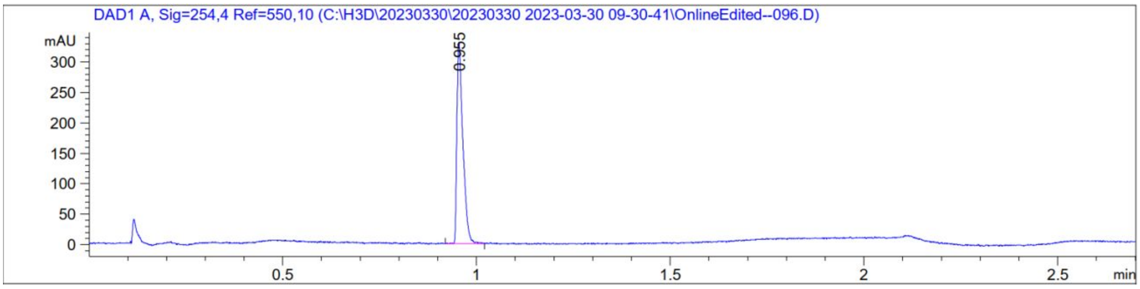

Signal 1: DAD1 A, Sig=254,4 Ref=550,10

Peak #	RetTime [min]	Type	Width [min]	Area [mAU*s]	Height [mAU]	Area %
1	0.955	BB	0.0160	354.84399	330.65125	100.0000
Totals :				354.84399	330.65125	

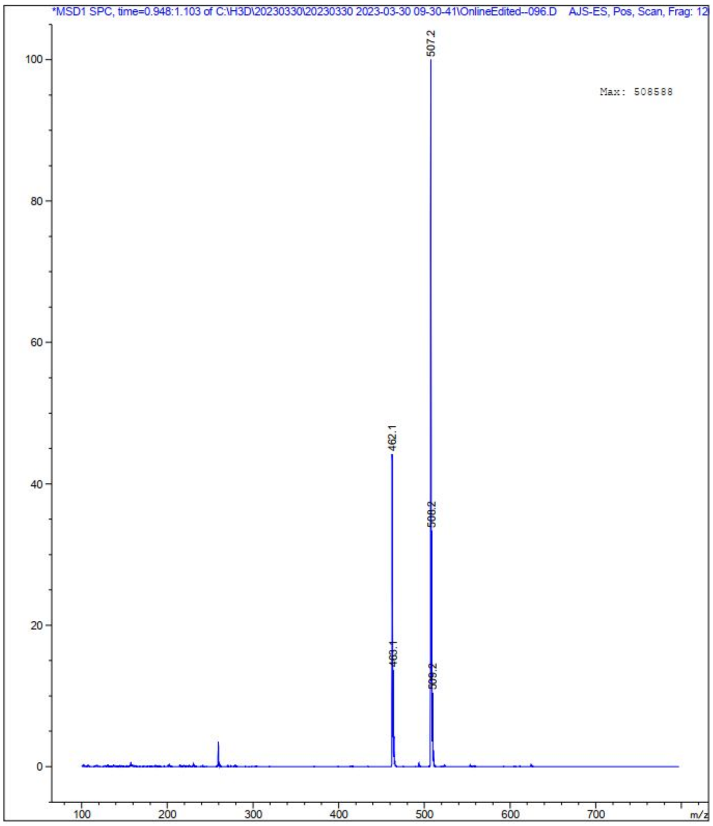

Figure S23. ^{1}H NMR (300 MHz, MeOD) δ 7.63 (s, 1H), 7.53 – 7.45 (m, 3H), 7.44 – 7.39 (m, 4H), 7.12 (t, 2H, J = 8.3 Hz), 4.74 (s, 2H), 4.27 (q, 2H, J = 7.1 Hz), 3.90 (s, 3H), 3.79 (s, 2H), 3.72 (s, 3H), 2.42 (s, 6H), 1.36 (t, 3H, J = 7.1 Hz)

**Trace impurities were observed at δ 4.81, 3.15 & 3.05 ppm.

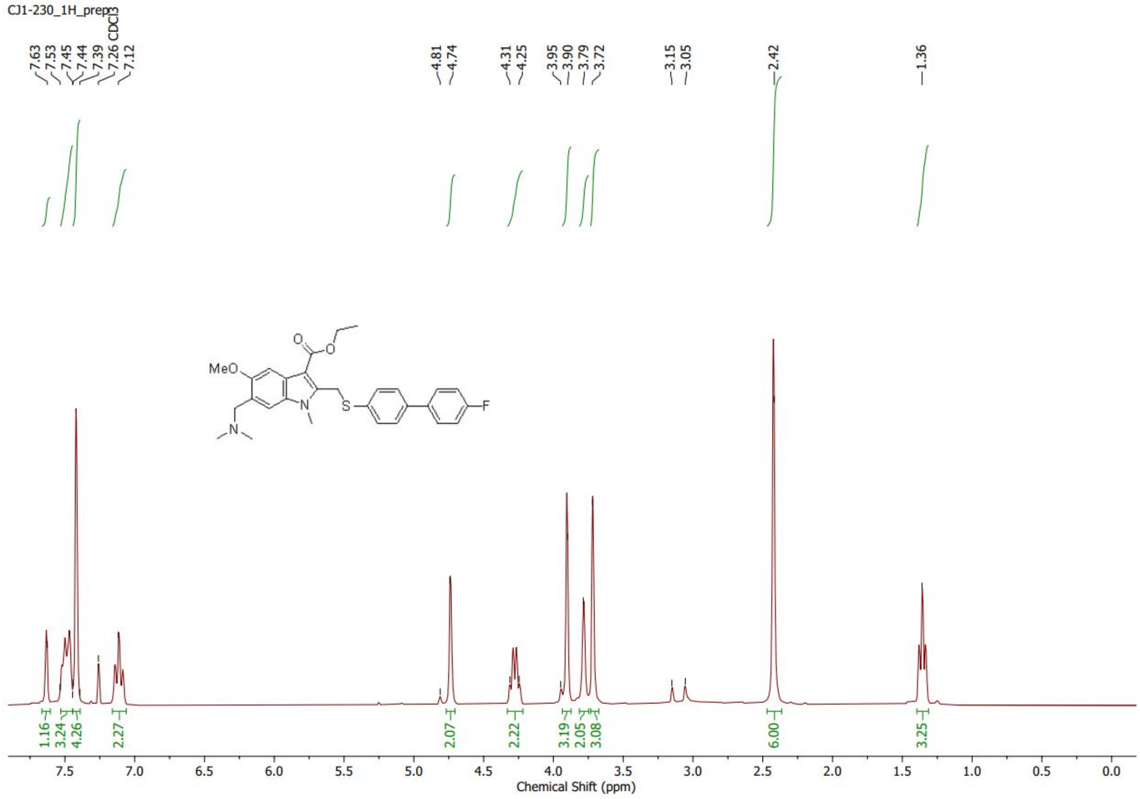

Compound 30 - Ethyl 6-((dimethylamino)methyl)-5-methoxy-1-methyl-2-(((4-(6-(trifluoromethyl)pyridin-3-yl)phenyl)thio)methyl)-1*H*-indole-3-carboxylate – white solid, 2% yield.

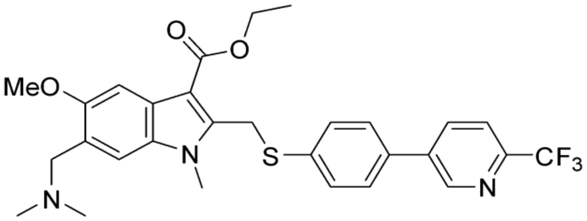

Figure S24. LC-MS ESI$^+$. t_R = 0.933 min (purity 100%); exact mass calcd for $C_{29}H_{30}F_3N_3O_3S$: 557.19, found: *m/z* 558.2 $[M + H]^+$.

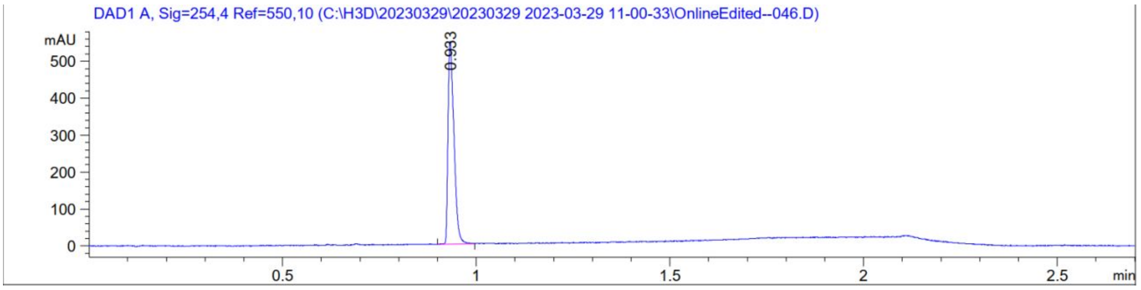

Signal 1: DAD1 A, Sig=254,4 Ref=550,10

Peak #	RetTime [min]	Type	Width [min]	Area [mAU*s]	Height [mAU]	Area %
1	0.933	BB	0.0169	582.59320	547.48035	100.0000
Totals :				582.59320	547.48035	

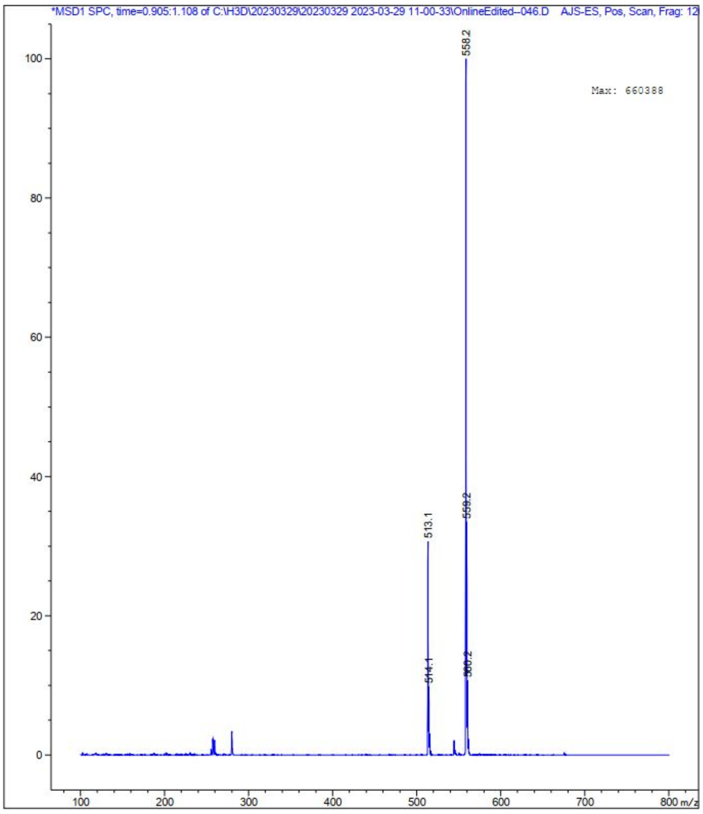

Figure S25. ^{1}H NMR (300 MHz, MeOD) δ 8.91 (s, 1H), 8.23 (d, 1H, J = 8.40 Hz), 7.86 (d, 1H, J = 8.40 Hz), 7.62 – 7.59 (m, 3H), 7.49 – 7.46 (m, 3H), 4.68 – 4.53 (m, 2H),* 4.23 (q, 2H, J = 7.1 Hz), 3.99 (s, 2H), 3.90 (s, 3H), 3.80 (s, 3H), 2.55 (s, 6H), 1.34 (t, 3H, J = 7.1 Hz).

* A shoulder was observed upfield of the MeOD H_2O peak. This is as a result of the overlap between the water peak and the signal of the methylene protons alpha to the sulfur atom.

**Trace impurities were observed at δ 7.06, 2.31 & 1.28 ppm.

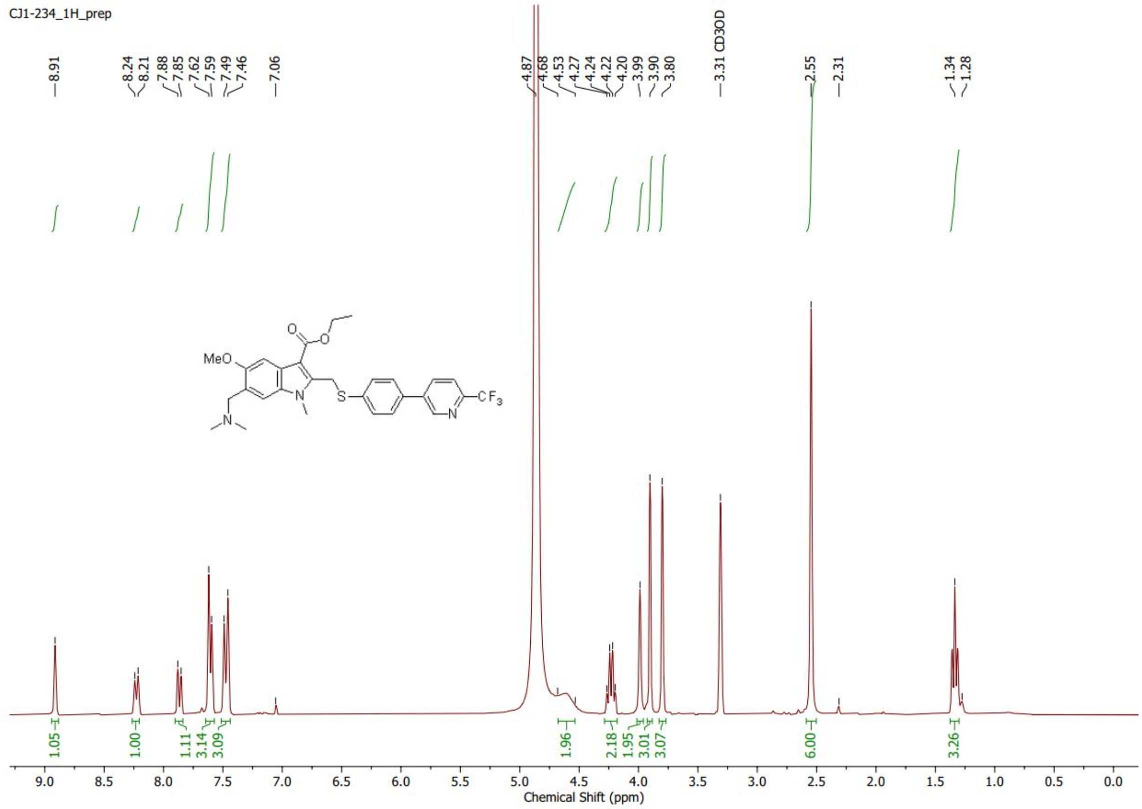

Chemical Synthesis - Mannich reaction for Cyclopropyl and Cyclopropyl Methane derivatives - Method A

N,N,N',N'-Tetramethyldiaminomethane (6.1 equiv) was added slowly to the appropriate indole (1.0 equiv, 500 mg scale) in a sealable vial under nitrogen at 0 °C. Glacial acetic acid (5.0 mL) was then added carefully to the vial. The vial was sealed and removed from the ice bath before being heated at 140 °C for 16 h. At this time, LC-MS analysis indicated the presence of primarily starting material and a trace amount of both Mannich regioisomers. After cooling to room temperature, excess acetic acid was removed under reduced pressure, and the crude product was purified via reverse-phase flash column chromatography using a Teledyne ISCO CombiFlash system eluting a gradient of MeCN + 0.1% formic acid in H_2O + 0.1% formic acid (typically 10 – 100%). Once dried via lyophilization, the product was dissolved in 20% MeOH:DCM and loaded onto a 2 g SCX column (propylsulfonic acid). The column was washed with 20% MeOH:DCM, and the product was eluted with 3.5 M NH_3 in MeOH before being lyophilized again to afford the product as the freebase (1 – 7% yield).

Compound 12 - Ethyl 1-cyclopropyl-6-((dimethylamino)methyl)-5-methoxy-2-((phenylthio)methyl)-1*H*-indole-3-carboxylate – brown solid, 5% yield

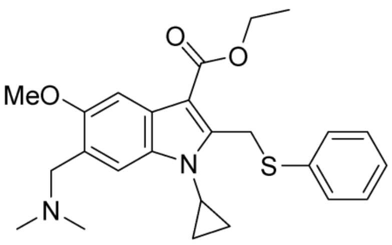

Figure S26. LC-MS ESI$^+$. t_R = 0.864 min (purity 100%); exact mass calcd for $C_{25}H_{30}N_2O_3S$: 438.19, found: *m/z* 439.2 $[M + H]^+$.

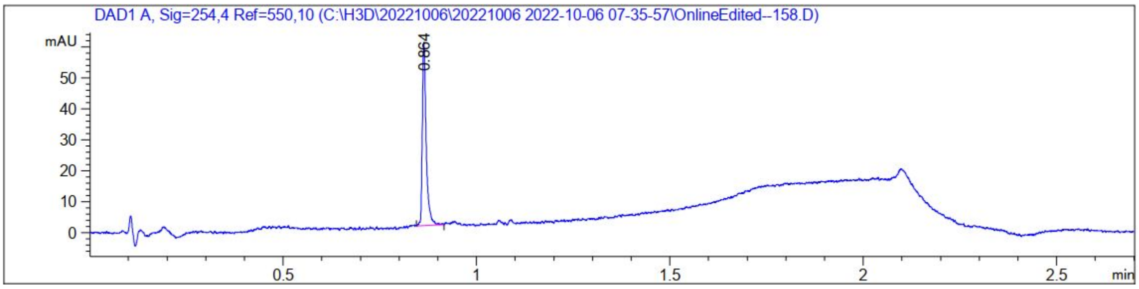

Signal 1: DAD1 A, Sig=254,4 Ref=550,10

Peak #	RetTime [min]	Type	Width [min]	Area [mAU*s]	Height [mAU]	Area %
1	0.864	BB	0.0107	41.07944	58.34928	100.0000
Totals :				41.07944	58.34928	

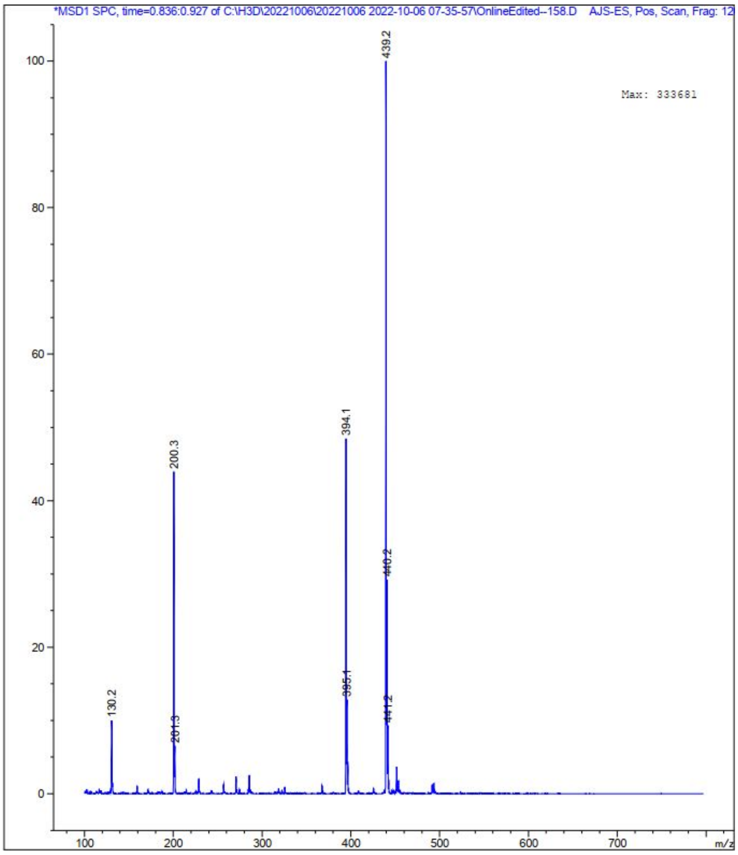

Figure S27. ^{1}H NMR (300 MHz, $CDCl_3$) δ 7.97 (s, 1H), 7.68 (s, 1H), 7.41 – 7.31 (m, 2H), 7.30 – 7.20 (m, 3H), 4.83 (s, 2H), 4.35 (s, 2H), 4.32 – 4.19 (m, 2H), 3.91 (s, 3H), 3.09 – 2.99 (m, 1H), 2.74 (s, 6H), 1.36 (t, 3H, J = 7.2 Hz), 1.27 – 1.11 (m, 4H).

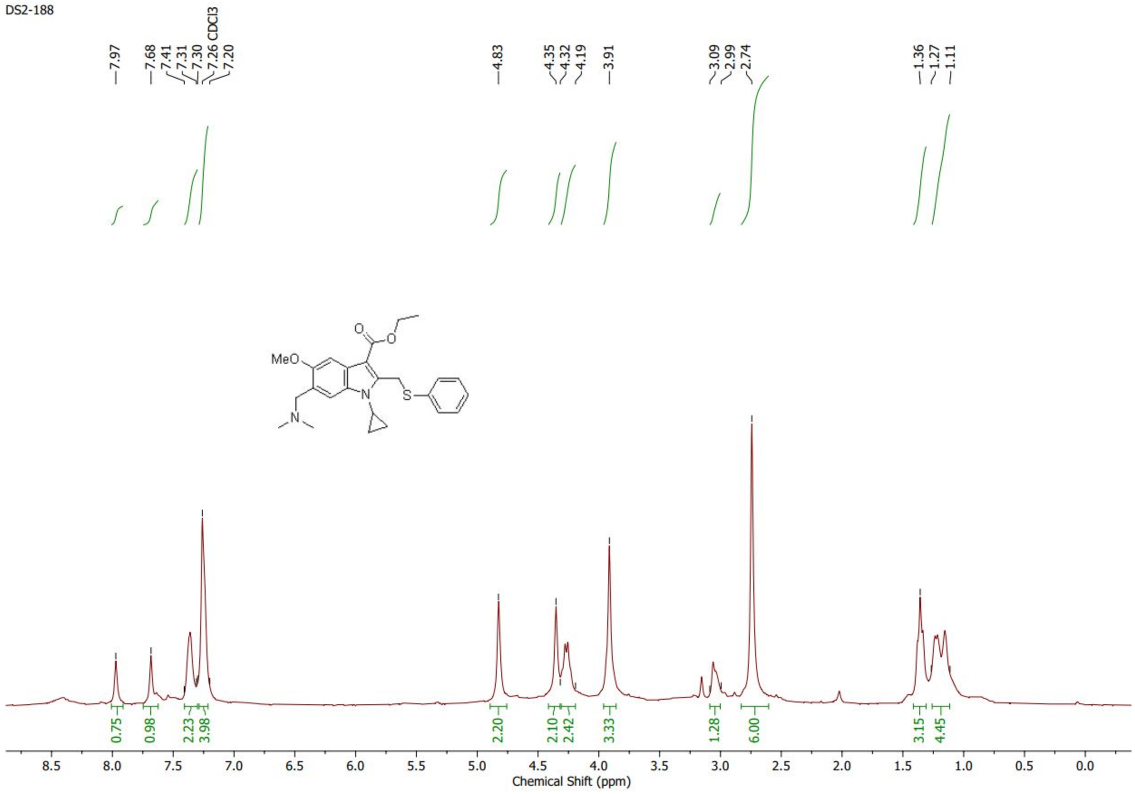

Compound 19 - Ethyl 2-[(4-bromophenyl)sulfanylmethyl]-1-cyclopropyl-6-[(dimethylamino)methyl]-5-methoxyindole-3-carboxylate – off-white solid, 2% yield.

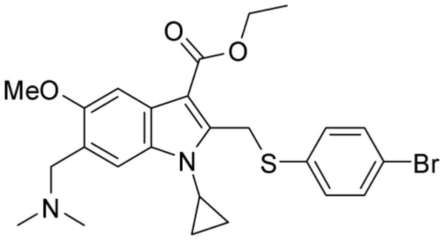

Figure S28. LC-MS ESI$^+$. t_R = 0.925 min (purity 100%); exact mass calcd for $C_{25}H_{29}BrN_2O_3S$: 516.10, found: m/z 517.10 $[M + H]^+$.

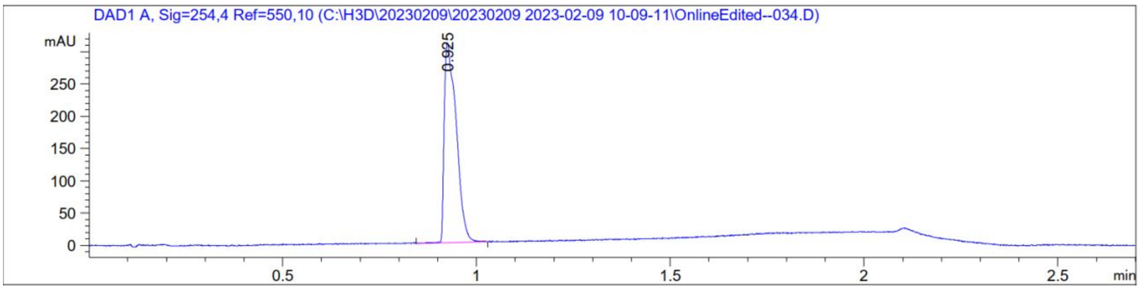

Signal 1: DAD1 A, Sig=254,4 Ref=550,10

Peak #	RetTime [min]	Type	Width [min]	Area [mAU*s]	Height [mAU]	Area %
1	0.925	BB	0.0297	668.66632	309.06693	100.0000
Totals :				668.66632	309.06693	

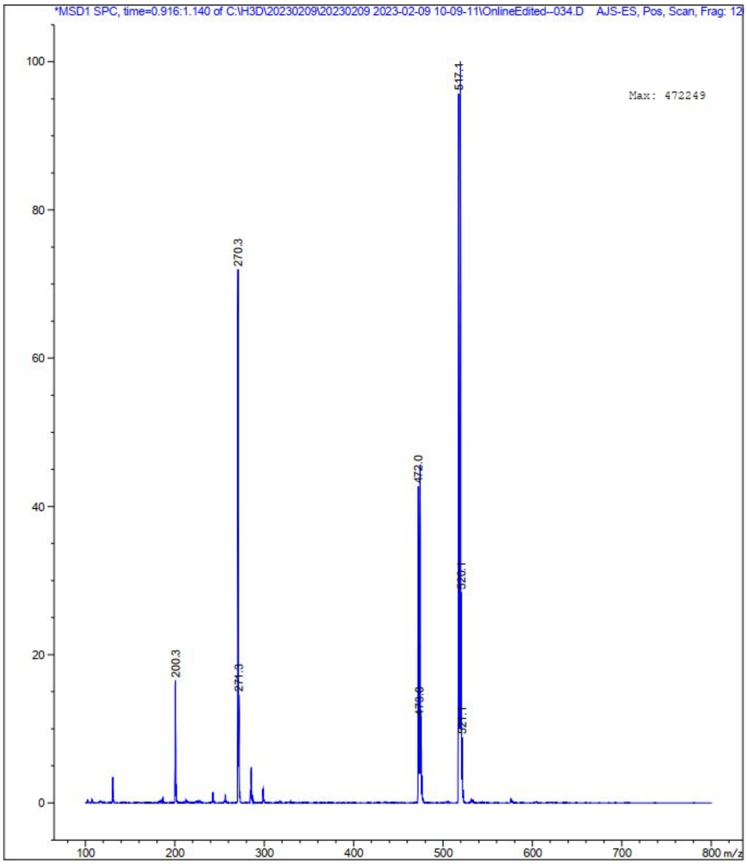

Figure S29. ^{1}H NMR (300 MHz, MeOD) δ 7.68 (s, 1H), 7.65 (s, 1H), 7.37 (d, 2H, *J* = 8.50 Hz), 7.21 (d, 2H, *J* = 8.50 Hz), 4.22 (q, 2H, *J* = 7.1 Hz)**,*** 4.19 (s, 2H)**,*** 3.93 (s, 3H), 3.23 – 3.16 (m, 1H), 2.69 (s, 6H), 1.36 (t, 3H, *J* = 7.1 Hz), 1.30 – 1.24 (m, 2H), 1.16 – 1.10 (m, 2H).

* As shown on the expansion in the ^{1}H NMR spectroscopic data, the quartet of the ester group overlaps with the signal of the Mannich methylene protons.

** The MeOD water signal masks the signal of the thioether methylene protons at δ 4.86 ppm.

*** Despite freebasing the product via SCX, a residual formate signal was observed at δ 8.54 ppm. The product has been reported as the freebase.

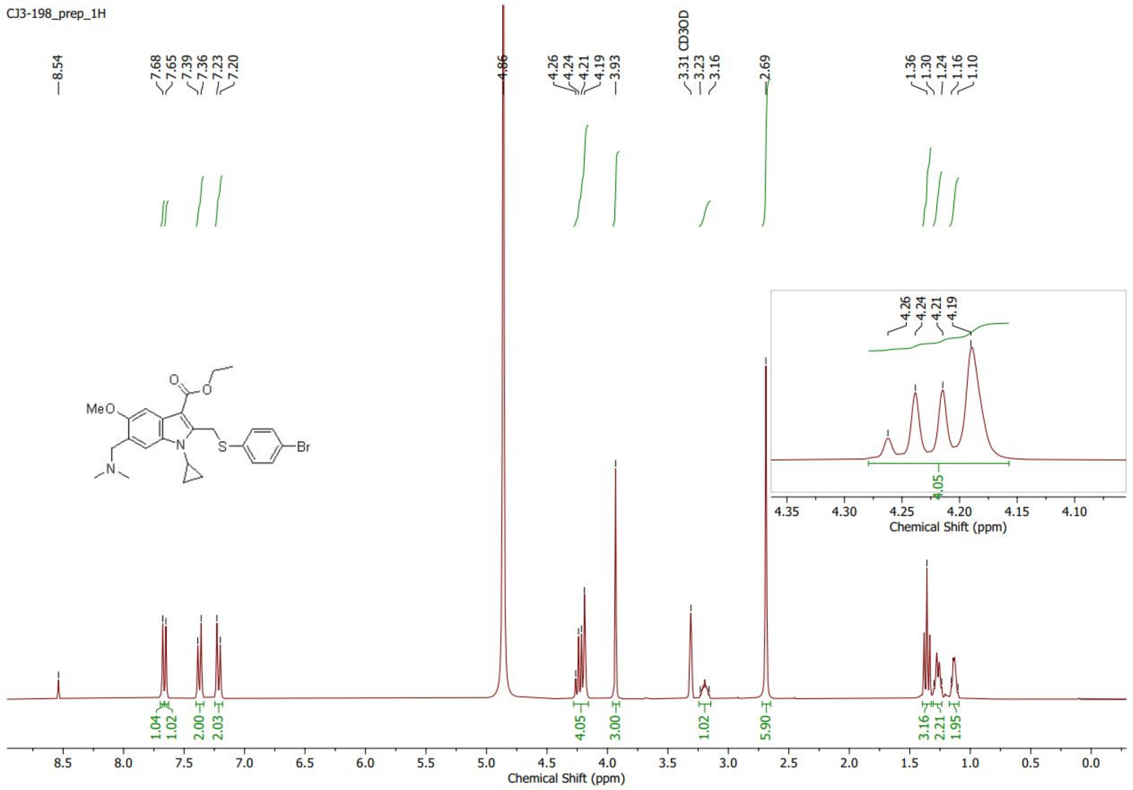

Compound 22 - Ethyl 1-cyclopropyl-6-((dimethylamino)methyl)-2-(((4-fluorophenyl)thio)methyl)-5-methoxy-1*H*-indole-3-carboxylate – brown solid, 4% yield.

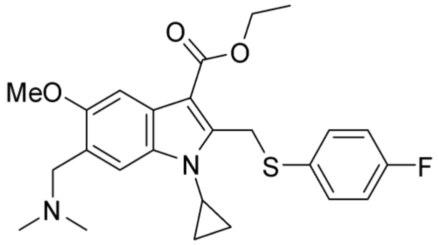

Figure S30. LC-MS ESI$^+$. t_R = 0.877 min (purity 100%); exact mass calcd for $C_{25}H_{29}FN_2O_3S$: 456.18, found: *m/z* 457.2 $[M + H]^+$.

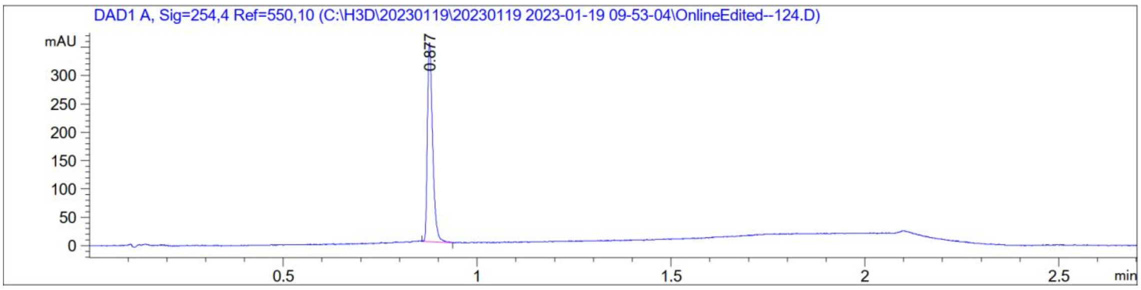

Signal 1: DAD1 A, Sig=254,4 Ref=550,10

Peak #	RetTime [min]	Type	Width [min]	Area [mAU*s]	Height [mAU]	Area %
1	0.877	BB	0.0146	331.36057	349.15717	100.0000
Totals :				331.36057	349.15717	

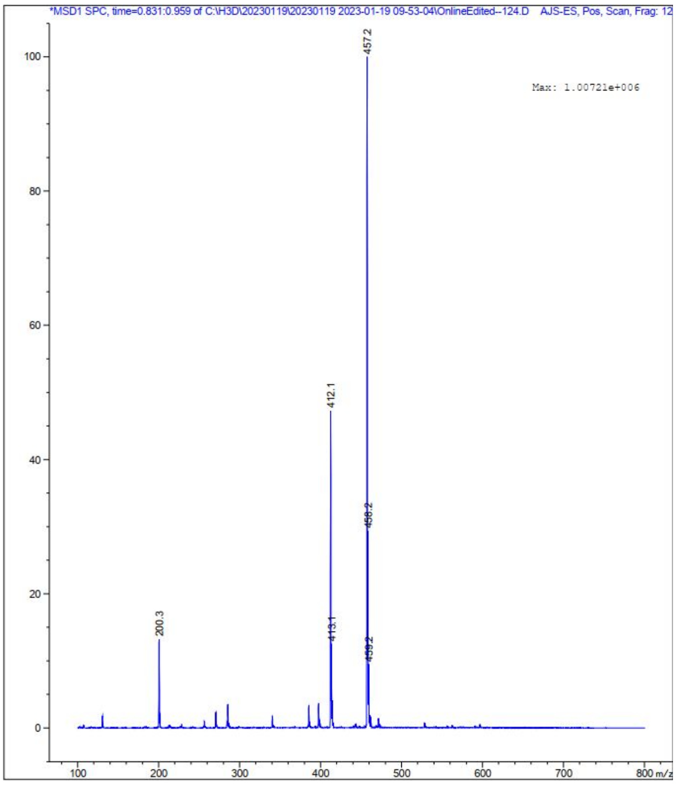

Figure S31. ^{1}**H NMR (300 MHz, MeOD)** δ 7.76 (s, 1H), 7.67 (s, 1H), 7.33 – 7.27 (m, 2H), 6.99 – 6.92 (m, 2H), 4.81 (s, 2H), 4.45 (s, 2H), 4.20 (q, *J* = 7.1 Hz, 2H), 3.96 (s, 3H), 3.18 – 3.11 (m, 1H), 2.88 (s, 6H), 1.35 (t, *J* = 7.1 Hz, 3H), 1.30 – 1.24 (m, 2H), 1.15 – 1.09 (m, 2H).

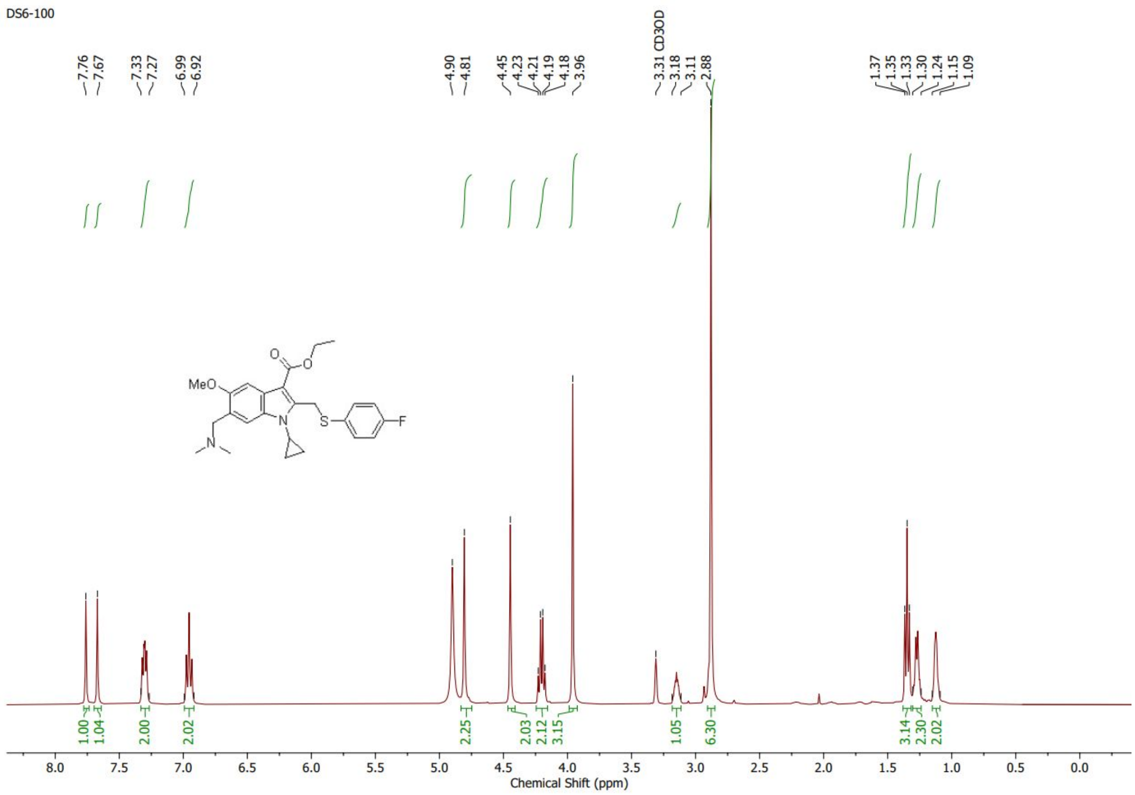

Compound 26 - Ethyl 1-cyclopropyl-6-((dimethylamino)methyl)-5-methoxy-2-((pyridin-2-ylthio)methyl)-1H-indole-3-carboxylate – brown syrup, 5% yield.

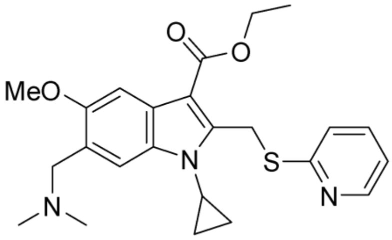

Figure S32. LC-MS ESI$^+$. t_R = 0.957 min (purity 98%); exact mass calcd for $C_{24}H_{29}N_3O_3S$: 439.19, found: *m/z* 440.2 [M + H]$^+$.

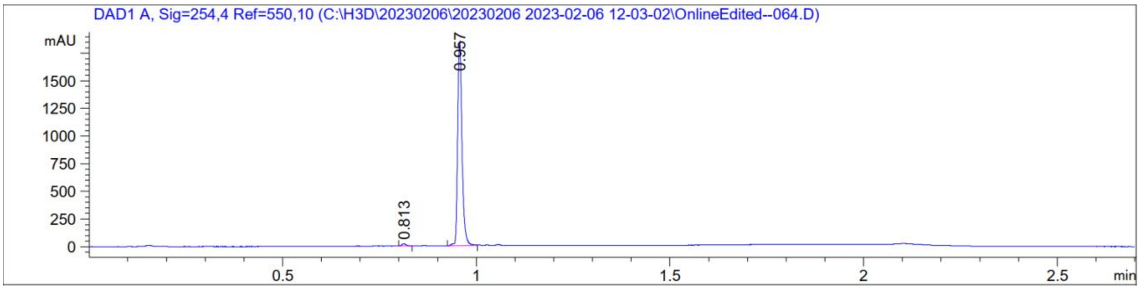

Signal 1: DAD1 A, Sig=254,4 Ref=550,10

Peak #	RetTime [min]	Type	Width [min]	Area [mAU*s]	Height [mAU]	Area %
1	0.813	BB	0.0113	15.45086	20.42572	1.0608
2	0.957	BB	0.0122	1441.10913	1836.29541	98.9392
Totals :				1456.56000	1856.72113	

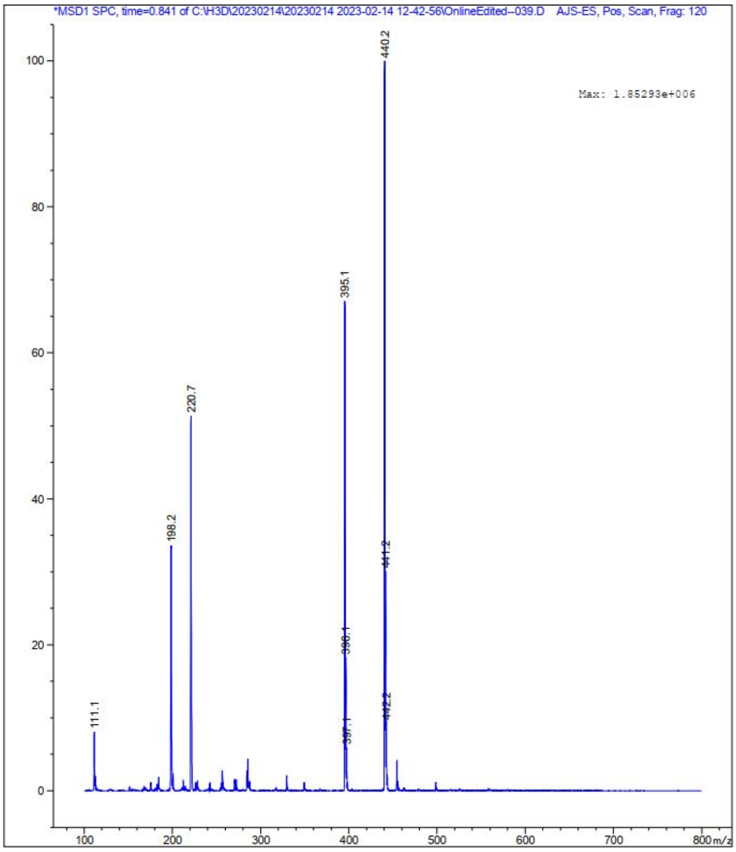

Figure S33. ^{1}H NMR (300 MHz, MeOD) δ 8.47 (d, *J* = 5.0 Hz, 1H), 7.70 (s, 1H), 7.66 (s, 1H), 7.66 – 7.61 (m, 1H),* 7.32 (d, *J* = 8.1 Hz, 1H), 7.17 – 7.12 (m, 1H), 5.21 (s, 2H), 4.35 (q, *J* = 7.1 Hz, 2H), 4.14 (br. s, 2H), 3.94 (s, 3H), 3.30 – 3.27 (m, 1H),** 2.65 (s, 6H), 1.39 (t, *J* = 7.1 Hz, 3H), 1.30 – 1.23 (m, 2H), 1.18 – 1.13 (m, 2H).

* This signal is overlapping with one of the indole singlets.

** The cyclopropyl methine signal is being masked by the MeOD solvent signal (see expansion)

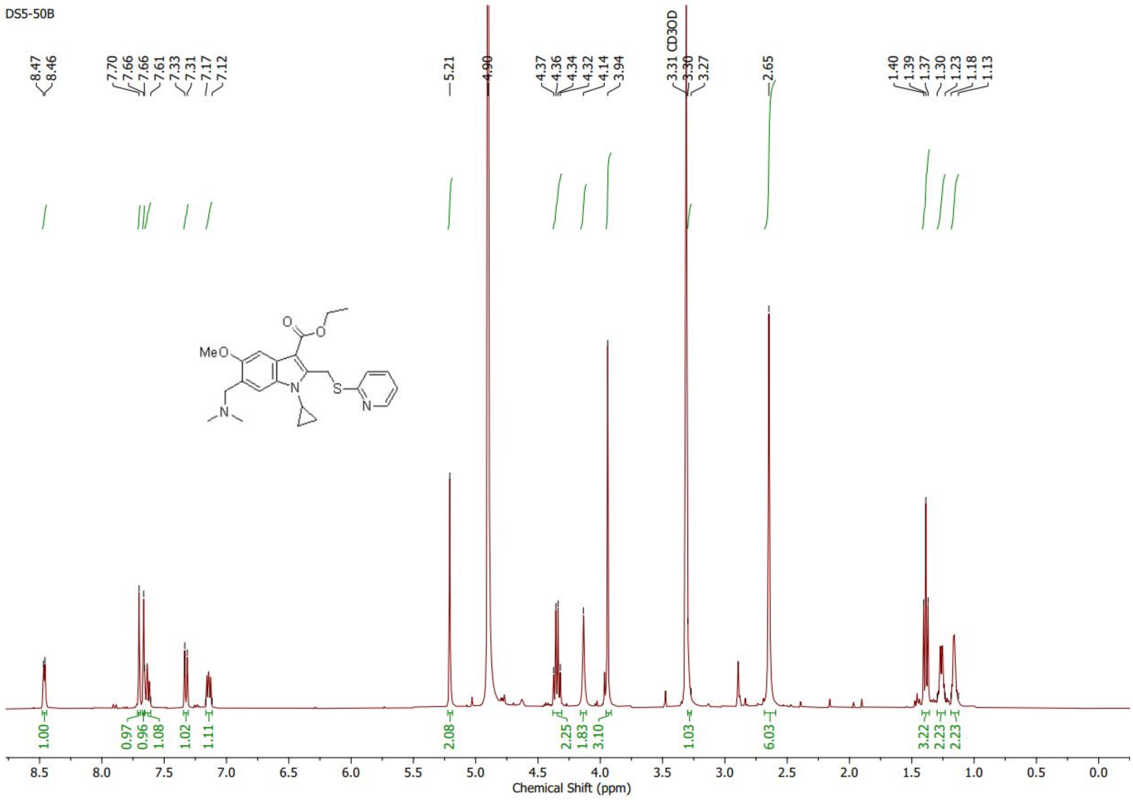

Compound 13 - Ethyl 1-(cyclopropylmethyl)-6-((dimethylamino)methyl)-5-methoxy-2-((phenylthio)methyl)-1H-indole-3-carboxylate – brown solid, 3% yield.

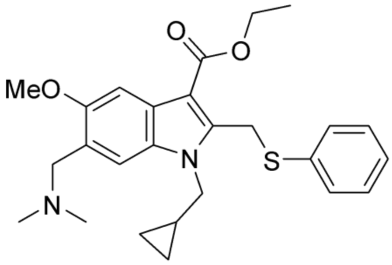

Figure S34. LC-MS ESI$^+$. t_R = 0.888 min (purity 100%); exact mass calcd for $C_{26}H_{32}N_2O_3S$: 452.21, found: m/z 453.2 $[M + H]^+$.

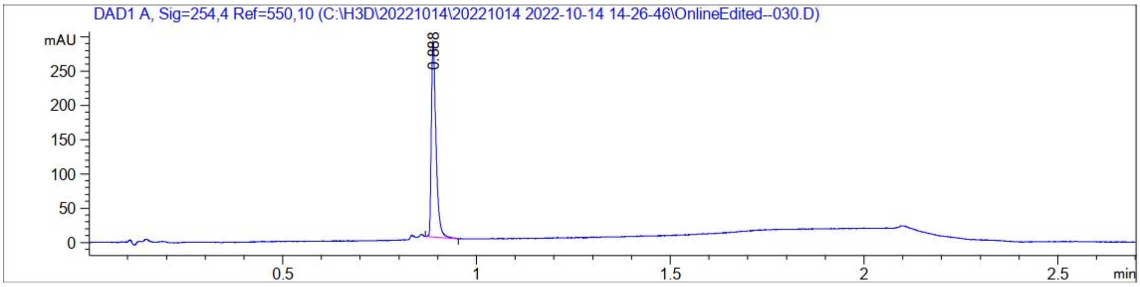

Signal 1: DAD1 A, Sig=254,4 Ref=550,10

Peak #	RetTime [min]	Type	Width [min]	Area [mAU*s]	Height [mAU]	Area %
1	0.888	BB	0.0128	237.86665	284.46750	100.0000

Totals :	237.86665	284.46750

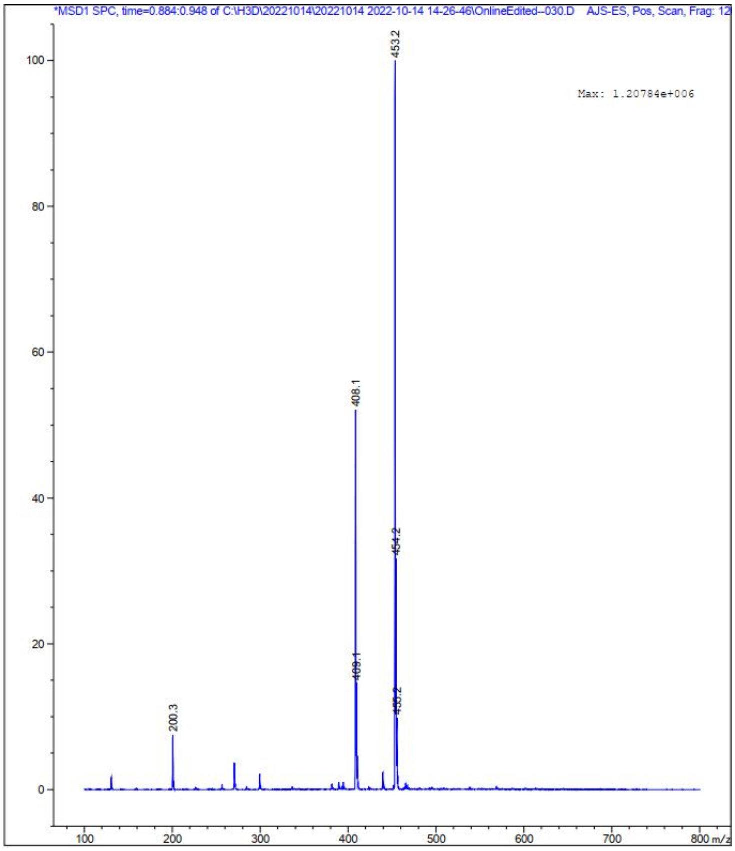

Figure S35. ^{1}H NMR (300 MHz, CDCl$_3$) δ 7.65 (s, 1H), 7.55 (s, 1H), 7.39 – 7.33 (m, 2H), 7.28 – 7.23 (m, 4H),* 4.72 (s, 2H), 4.28 (q, *J* = 7.1 Hz, 2H), 4.04 (d, 2H, *J* = 6.5 Hz), 3.93 – 3.87 (m, 5H), 2.47 (s, 6H), 1.36 (t, 3H, *J* = 7.1 Hz), 1.24 – 1.16 (m, 1H), 0.57 – 0.49 (m, 2H), 0.39 – 0.33 (m, 2H).

**One extra proton due to overlap with CDCl$_3$ signal*

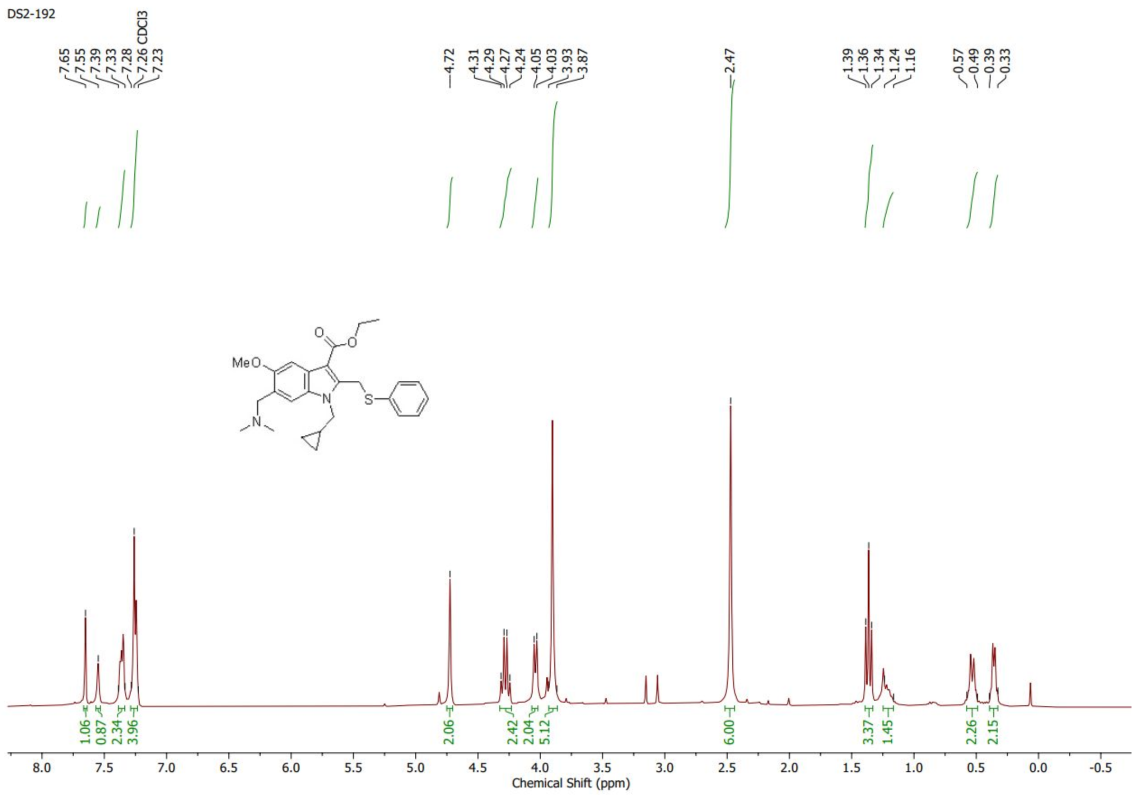

Compound 20 - Ethyl 2-(((4-bromophenyl)thio)methyl)-1-(cyclopropylmethyl)-6-((dimethylamino)methyl)-5-methoxy-1H-indole-3-carboxylate – pale-yellow solid, 4% yield.

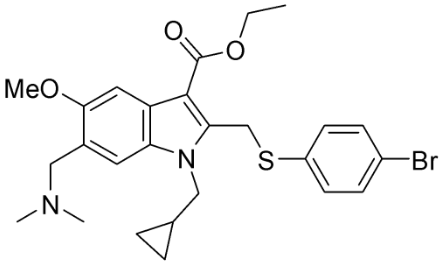

Figure S36. LC-MS ESI$^+$. t_R = 1.129 min (purity 98%); exact mass calcd for $C_{26}H_{31}BrN_2O_3S$: 530.12, found: *m/z* 531.1 $[M + H]^+$.

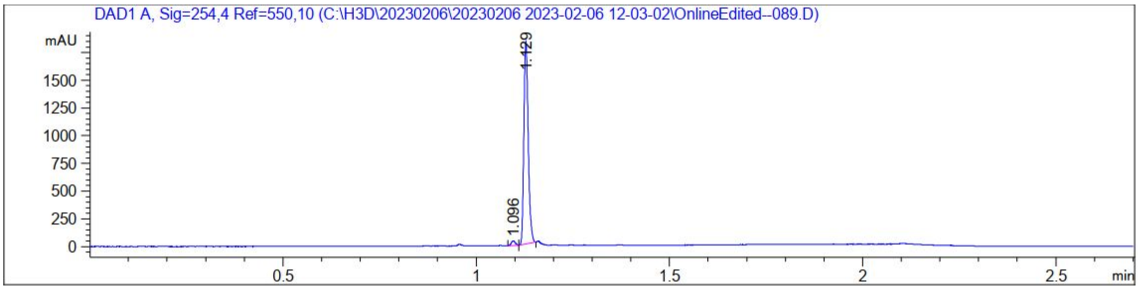

Signal 1: DAD1 A, Sig=254,4 Ref=550,10

Peak #	RetTime [min]	Type	Width [min]	Area [mAU*s]	Height [mAU]	Area %
1	1.096	BB	0.0112	28.86421	41.24428	1.9645
2	1.129	BB	0.0123	1440.40747	1807.84277	98.0355

Totals :				1469.27168	1849.08705	

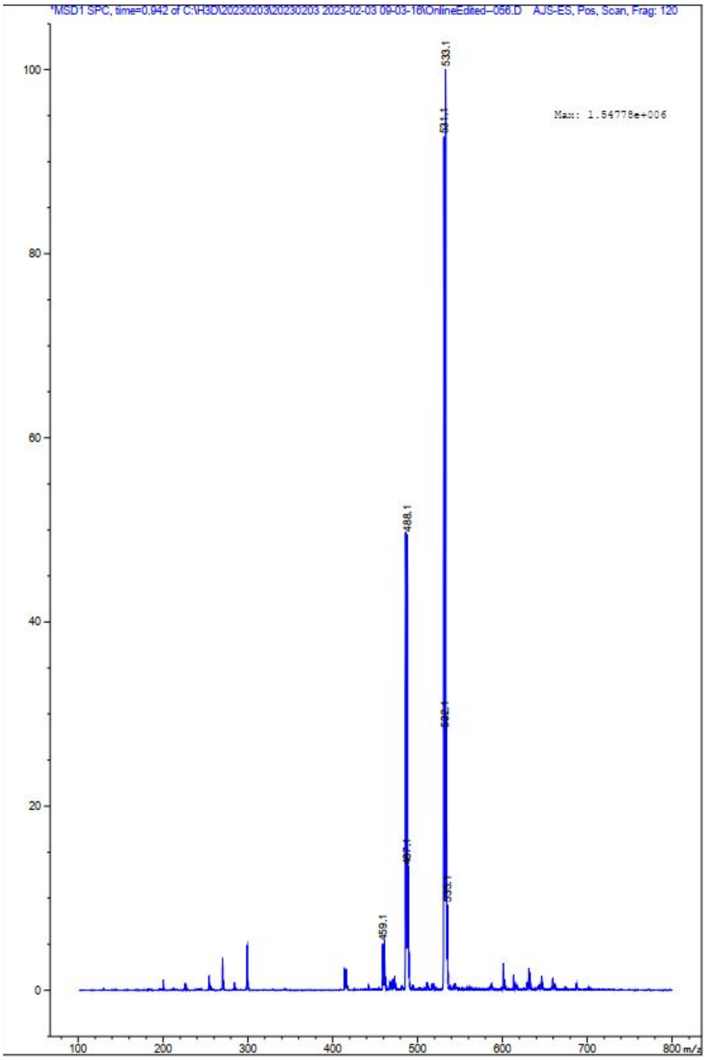

Figure S37. ^{1}H NMR (300 MHz, DMSO) δ 7.78 (s, 1H), 7.58 (s, 1H), 7.50 (d, *J* = 8.4 Hz, 2H), 7.31 (d, *J* = 8.3 Hz, 2H), 4.84 (s, 2H), 4.33 (s, 2H), 4.25 – 4.12 (m, 4H), 3.88 (s, 3H), 2.73 (s, 6H)Z, 1.33 – 1.25 (m, 4H), 0.52 – 0.43 (m, 4H).

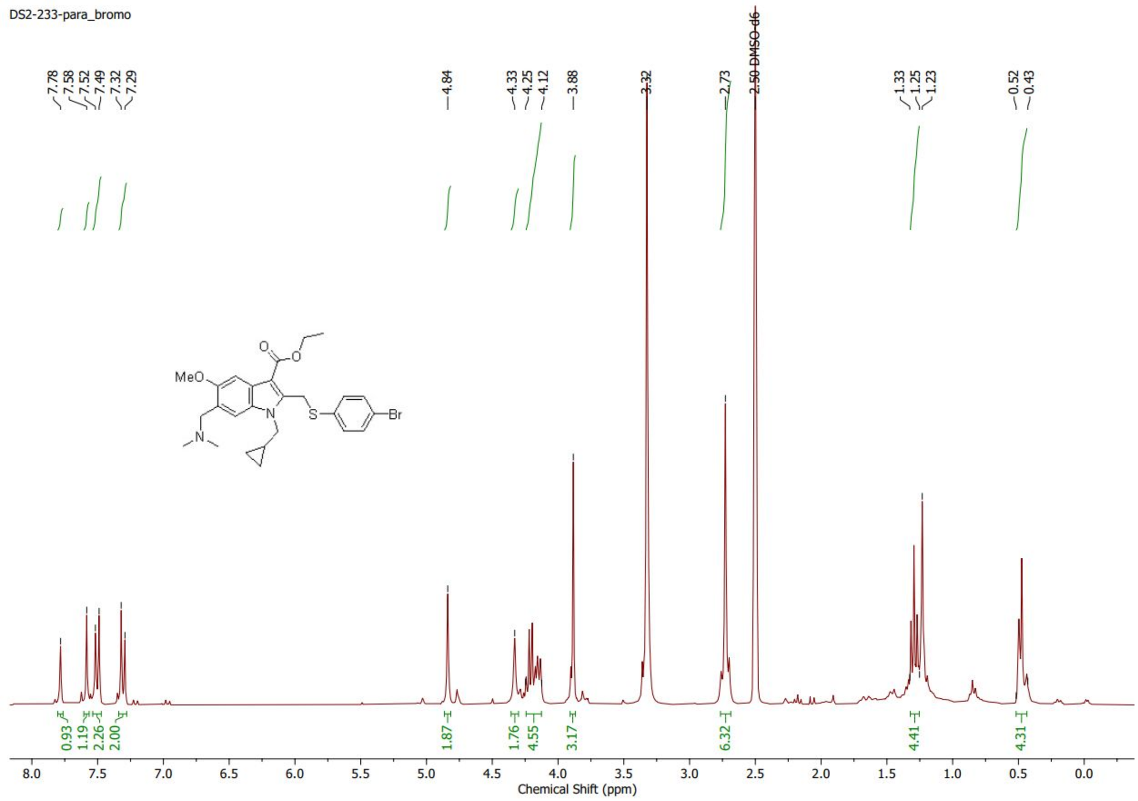

Compound 24 - Ethyl 1-(cyclopropylmethyl)-6-((dimethylamino)methyl)-5-methoxy-2-(((4-methoxyphenyl)thio)methyl)-1H-indole-3-carboxylate – pale-brown solid, 1% yield.

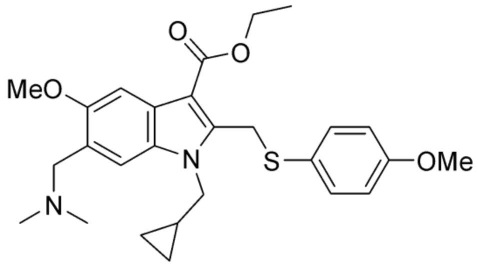

Figure S38. LC-MS ESI$^+$. t_R = 1.001 min (purity 99%); exact mass calcd for $C_{27}H_{34}N_2O_4S$: 482.22, found: *m/z* 483.2 $[M + H]^+$.

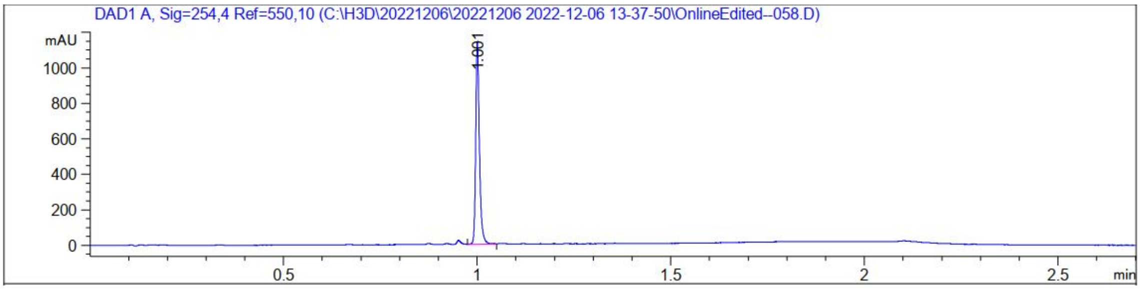

Signal 1: DAD1 A, Sig=254,4 Ref=550,10

Peak #	RetTime [min]	Type	Width [min]	Area [mAU*s]	Height [mAU]	Area %
1	1.001	BB	0.0103	752.11865	1130.70703	100.0000

Totals : 752.11865 1130.70703

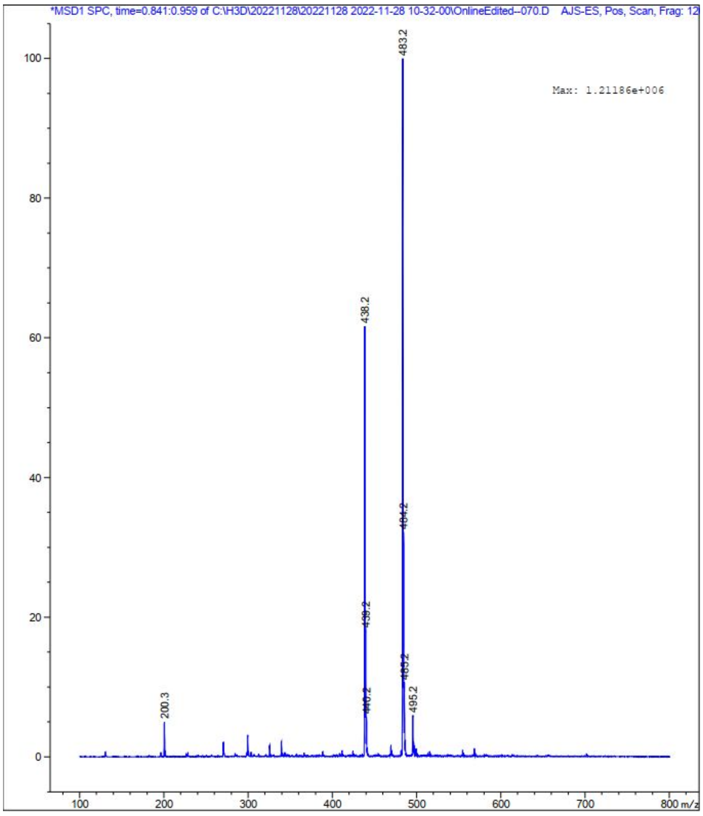

Figure S39. ^{1}H NMR (300 MHz, $CDCl_3$) δ 7.59 – 7.57 (m, 2H), 7.21 – 7.18 (m, 2H), 6.69 (d, *J* = 8.6 Hz, 2H), 4.53 (s, 2H), 4.18 (q, *J* = 7.1 Hz, 2H), 4.01 (s, 2H), 3.94 (d, *J* = 6.6 Hz, 2H), 3.84 (s, 3H), 3.70 (s, 3H), 2.49 (s, 6H), 1.29 (t, *J* = 7.1 Hz, 3H), 1.20 – 1.13 (m, 1H), 0.47 – 0.42 (m, 2H), 0.32 – 0.26 (m, 2H).

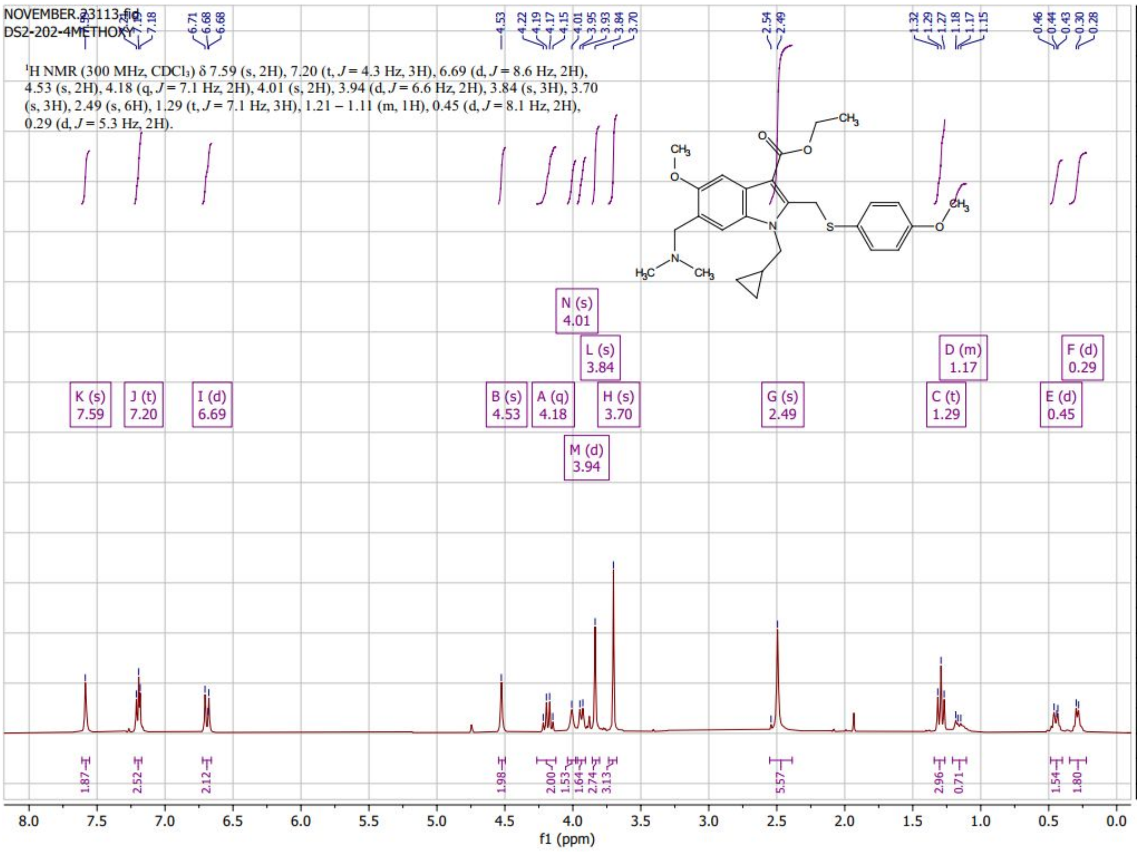

Compound 23 - Ethyl 1-(cyclopropylmethyl)-6-((dimethylamino)methyl)-2-(((4-fluorophenyl)thio)methyl)-5-methoxy-1H-indole-3-carboxylate – brown solid, 2% yield.

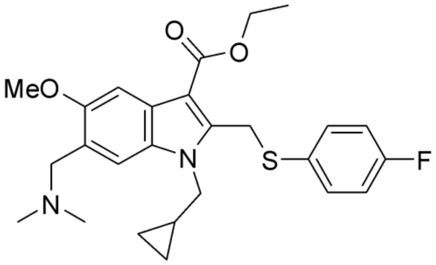

Figure S40. LC-MS ESI$^+$. t_R = 0.897 min (purity 99%); exact mass calcd for $C_{26}H_{31}FN_2O_3S$: 470.20, found: m/z 471.2 $[M + H]^+$.

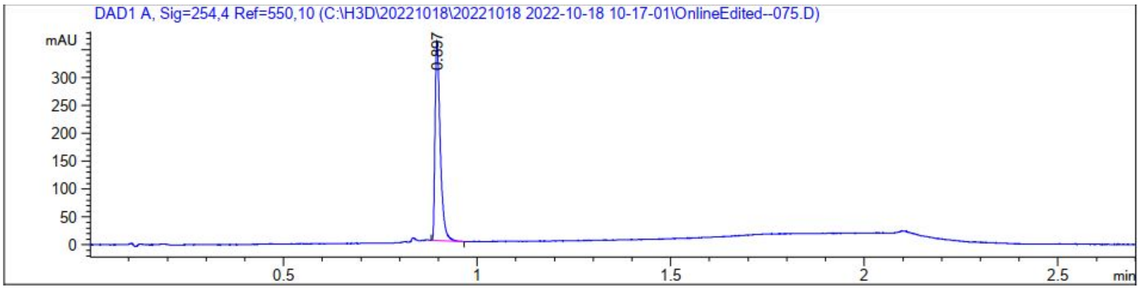

Signal 1: DAD1 A, Sig=254,4 Ref=550,10

Peak #	RetTime [min]	Type	Width [min]	Area [mAU*s]	Height [mAU]	Area %
1	0.897	BB	0.0146	339.60016	356.66461	100.0000

Totals : 339.60016 356.66461

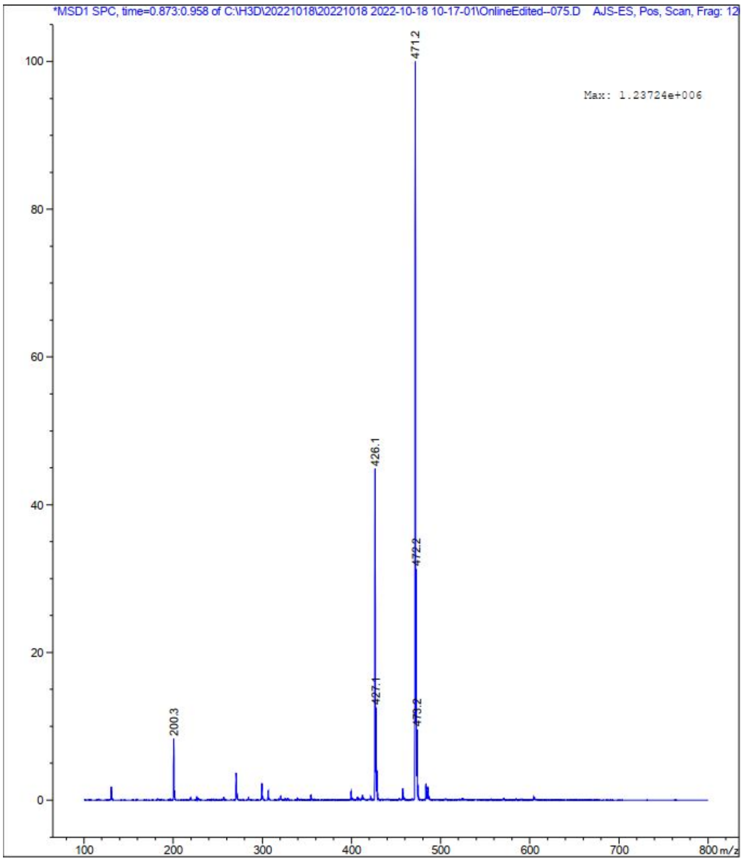

Figure S41. ^{1}H NMR (300 MHz, $CDCl_3$) δ 7.39 (s, 1H), 7.28 (s, 1H), 7.10 – 7.06 (m, 2H), 6.75 – 6.64 (m, 2H), 4.44 (s, 2H), 4.02 (q, *J* = 7.1 Hz, 2H), 3.83 (d, *J* = 6.4 Hz, 2H), 3.68 (s, 3H), 3.61 (s, 2H), 2.22 (s, 6H), 1.14 (t, *J* = 7.1 Hz, 3H), 1.04 – 0.97 (m, 1H), 0.36 – 0.27 (m, 2H), 0.17 – 0.10 (m, 2H).

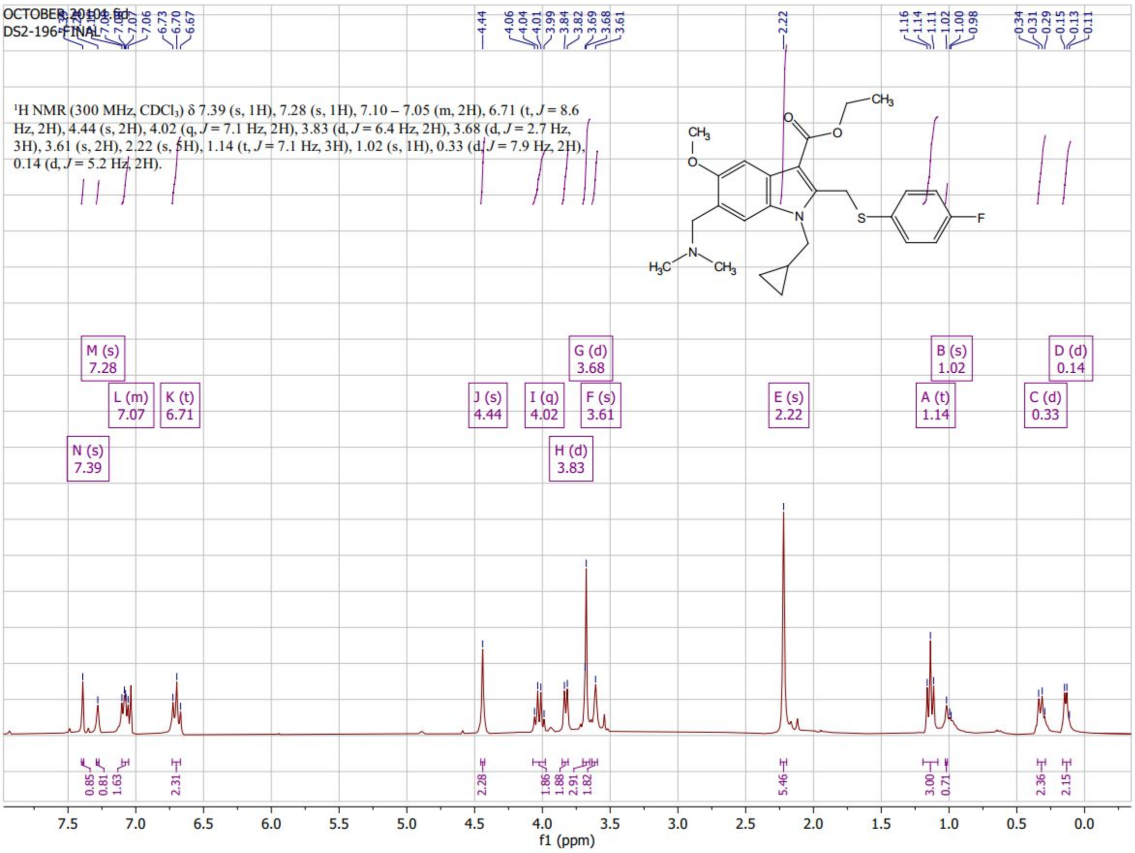

Vilsmeier-Haack Formylation – Method B

Phosphorus oxychloride (2.0 equiv) was carefully added to DMF (C = 0.40 M) in an ice-cooled vial under nitrogen with rapid stirring. The reaction mixture was stirred at room temperature for 40 min, after which the dark-red solution containing the Vilsmeier-Haack intermediate was cooled to 0 °C. The appropriate indole (1.0 equiv) was added portion wise, and the reaction mixture was allowed to warm to room temperature before being heated at 100 °C for 1 h and 15 min. At this time, LC-MS analysis indicated the formation of two formylated regioisomers. Upon cooling to room temperature, the reaction mixture was poured into ice water and quenched with 10% aqueous KOH. The aqueous phase was extracted with EtOAc (2X), and the organic layer was washed with H_2O before being dried over $MgSO_4$ and concentrated under reduced pressure. The crude residue was purified via silica gel flash column chromatography using a Teledyne ISCO Combiflash system eluting a gradient of EtOAc in hexane (typically 0 – 60%). The two regioisomers of the product were successfully separated and obtained in a ratio of approximately 1:1 (combined yield = 30 – 55%).

Major isomer: - ethyl 6-formyl-5-methoxy-1-methyl-2-((phenylthio)methyl)-1*H*-indole-3-carboxylate – yellow solid, 30% yield

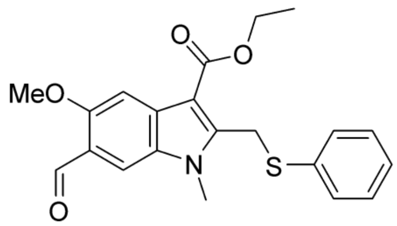

LC-MS ESI$^+$: t_R = 1.250 min (purity 96%); exact mass calcd for $C_{21}H_{21}NO_4S$: 383.12, found: *m/z* 384.1 [M + H]$^+$.

^{1}H NMR (300 MHz, CDCl$_3$) δ 10.53 (s, 1H), 7.81 (s, 1H), 7.68 (s, 1H), 7.39 – 7.31 (m, 2H), 7.29 – 7.21 (m, 3H), 4.66 (s, 2H), 4.29 (q, 2H, *J* = 7.1 Hz), 3.98 (s, 3H), 3.61 (s, 3H), 1.38 (t, 3H, *J* = 7.1 Hz).

Minor isomer: - ethyl 4-formyl-5-methoxy-1-methyl-2-((phenylthio)methyl)-1*H*-indole-3-carboxylate – yellow solid, 24% yield.

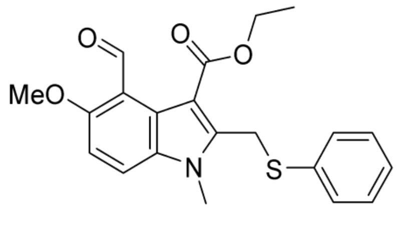

LC-MS ESI$^+$: t_R = 1.158 min (purity 92%); exact mass calcd for $C_{21}H_{21}NO_4S$: 383.12, found: *m/z* 384.1 [M + H]$^+$.

^{1}H NMR (300 MHz, CDCl$_3$) δ 10.60 (s, 1H), 7.42 (d, 1H, *J* = 9.1 Hz), 7.39 – 7.31 (m, 2H), 7.30 – 7.23 (m, 3H), 6.97 (d, 1H, *J* = 9.1 Hz), 4.52 (s, 2H), 4.21 (q, 2H, *J* = 7.1 Hz), 3.95 (s, 3H), 3.69 (s, 3H), 1.27 (t, 3H, *J* = 7.1 Hz).

* Impurities were noted in both ^{1}H NMR spectra.

Ethyl 2-((cyclohexylthio)methyl)-1-cyclopropyl-6-formyl-5-methoxy-1H-indole-3-carboxylate – yellow solid, 48% yield.

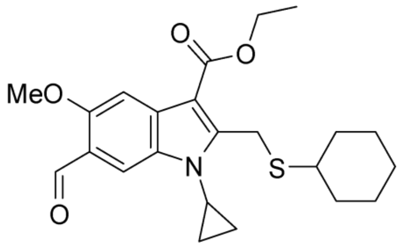

LC-MS ESI$^+$: t_R = 1.409 min (purity 97%); exact mass calcd for $C_{23}H_{29}NO_4S$: 415.18, found: *m/z* 416.2 [M + H]$^+$.

Ethyl 1-(cyclopropylmethyl)-6-formyl-5-methoxy-2-((*p*-tolylthio)methyl)-1H-indole-3-carboxylate – pale-yellow solid, 19% yield.

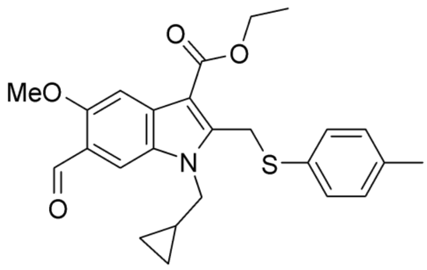

LC-MS ESI$^+$: t_R = 1.383 min (purity 85%); exact mass calcd for $C_{25}H_{27}NO_4S$: 437.16, found: *m/z* 438.2 [M + H]$^+$.

^{1}H NMR (300 MHz, CDCl$_3$) δ 10.54 (s, 1H), 7.87 (s, 1H), 7.72 (s, 1H), 7.24 (d, *J* = 7.7 Hz, 2H), 7.07 (d, *J* = 7.7 Hz, 2H), 4.66 (s, 2H), 4.31 (q, *J* = 7.1 Hz, 2H), 4.03 – 3.94 (m, 5H), 2.34 (s, 3H), 1.41 (t, *J* = 7.1 Hz, 3H), 1.22 – 1.17 (m, 1H), 0.59 – 0.54 (m, 2H), 0.36 – 0.33 (m, 2H).

Ethyl 1-(cyclopropylmethyl)-4-formyl-5-methoxy-2-((*p*-tolylthio)methyl)-1H-indole-3-carboxylate – brown oil, 16% yield.

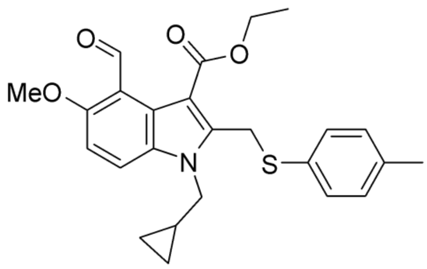

LC-MS ESI$^+$: t_R = 1.631 min (purity 69%); exact mass calcd for $C_{25}H_{27}NO_4S$: 437.16, found: *m/z* 438.2 [M + H]$^+$.

Ethyl 2-((cyclohexylthio)methyl)-1-(cyclopropylmethyl)-6-formyl-5-methoxy-1H-indole-3-carboxylate – brown oil, 57% yield.

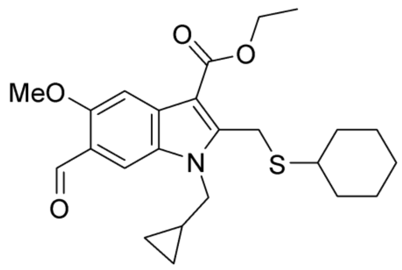

LC-MS ESI$^+$: t_R = 1.424 min (purity 73%); exact mass calcd for $C_{24}H_{31}NO_4S$: 429.20, found: *m/z* 430.2 [M + H]$^+$.

Ethyl 2-(((4-chlorophenyl)thio)methyl)-1-(cyclopropylmethyl)-6-formyl-5-methoxy-1H-indole-3-carboxylate – pale-orange solid, 19% yield.

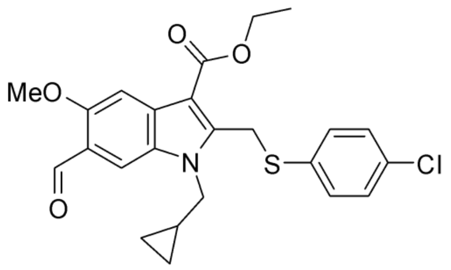

LC-MS ESI⁺: t_R = 1.306 min (purity 93%); exact mass calcd for $C_{24}H_{24}ClNO_4S$: 457.11, found: *m/z* 458.1 $[M + H]^+$.

Ethyl 1-(cyclopropylmethyl)-2-(((4'-fluoro-[1,1'-biphenyl]-4-yl)thio)methyl)-6-formyl-5-methoxy-1H-indole-3-carboxylate – pale-yellow oil, 59% yield.

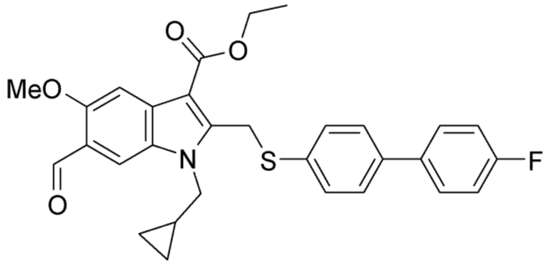

LC-MS ESI⁺: t_R = 1.660 min (purity 80%); exact mass calcd for $C_{30}H_{28}FNO_4S$: 517.17, found: *m/z* 518.1 $[M + H]^+$.

Ethyl 1-(cyclopropylmethyl)-6-formyl-5-methoxy-2-(((4-(6-(trifluoromethyl)pyridin-3-yl)phenyl)thio)methyl)-1H-indole-3-carboxylate – pale-yellow solid, 21% yield.

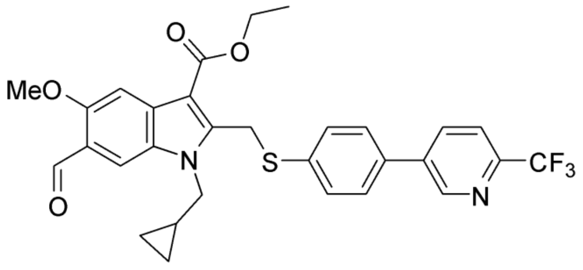

LC-MS ESI⁺: t_R = 1.416 min (purity 98%); exact mass calcd for $C_{30}H_{27}F_3N_2O_4S$: 568.16, found: *m/z* 569.2 $[M + H]^+$.

Reductive Amination – Method B

To a stirred solution of the appropriate formylated indole (1.0 equiv) in DCM (*C* = 0.15 M) in a sealable vial was added dimethylamine hydrochloride (5.0 equiv), followed by the portion-wise addition of sodium triacetoxyborohydride (2.0 equiv). The vial was sealed, and the reaction mixture was stirred at room temperature for 3 h (monitored via LC-MS analysis). The solvent was removed under reduced pressure, and the crude

product was purified via reverse-phase flash column chromatography using a 25 g C18 column with a Teledyne ISCO CombiFlash system eluting a gradient of MeCN + 0.1% formic acid in H_2O + 0.1% formic acid (typically 10 – 100%). Once dried via lyophilization, the product was dissolved in 20% MeOH in DCM and loaded onto a 2 g SCX column (propylsulfonic acid). The column was washed with 20% MeOH in DCM, and the product was eluted with 3.5 M NH_3 in MeOH before being lyophilized again to afford the product as the freebase (1 – 7% yield).

Compound 9 - Ethyl 2-((cyclohexylthio)methyl)-1-cyclopropyl-6-((dimethylamino)methyl)-5-methoxy-1H-indole-3-carboxylate – off-white solid, 40% yield.

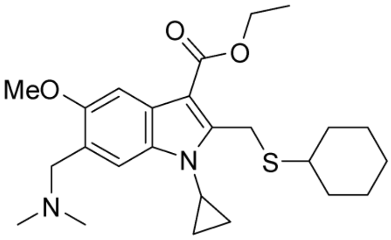

Figure S42. LC-MS ESI$^+$. t_R = 0.924 min (purity 100%); exact mass calcd for $C_{25}H_{36}N_2O_3S$: 444.24, found m/z 445.2 $[M+H]^+$.

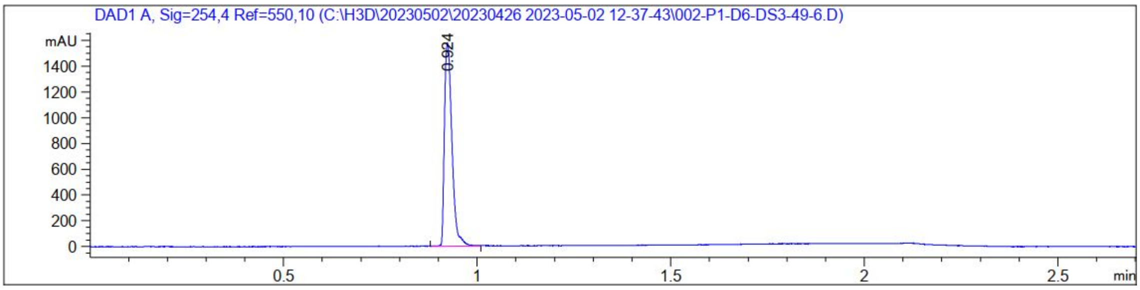


```
Signal 1: DAD1 A, Sig=254,4 Ref=550,10

Peak RetTime Type  Width     Area      Height     Area
  #   [min]        [min]   [mAU*s]     [mAU]        %
----|-------|----|-------|----------|----------|--------|
   1   0.924 BB    0.0207 2106.47339 1570.22559 100.0000

Totals :                  2106.47339 1570.22559
```

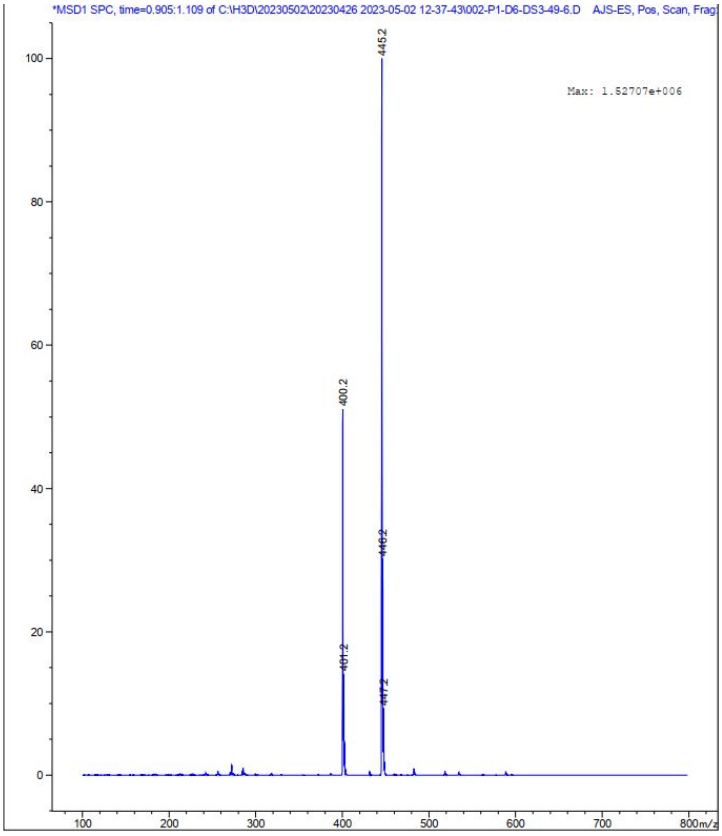

Figure S43. ^{1}H NMR (300 MHz, $CDCl_3$) δ 7.61 (s, 1H), 7.52 (s, 1H), 4.51 (s, 2H), 4.38 (q, 2H, *J* = 7.1 Hz), 3.88 (s, 3H), 3.61 (s, 2H), 3.37 – 3.28 (m, 1H), 2.75 – 2.64 (m, 1H), 2.32 (s, 6H), 2.03 – 1.93 (m, 2H), 1.77 – 1.67 (m, 2H), 1.61 – 1.52 (m, 1H), 1.44 (t, 3H, *J* = 7.1 Hz), 1.35 – 1.18 (m, 7H), 1.14 – 1.07 (m, 2H).

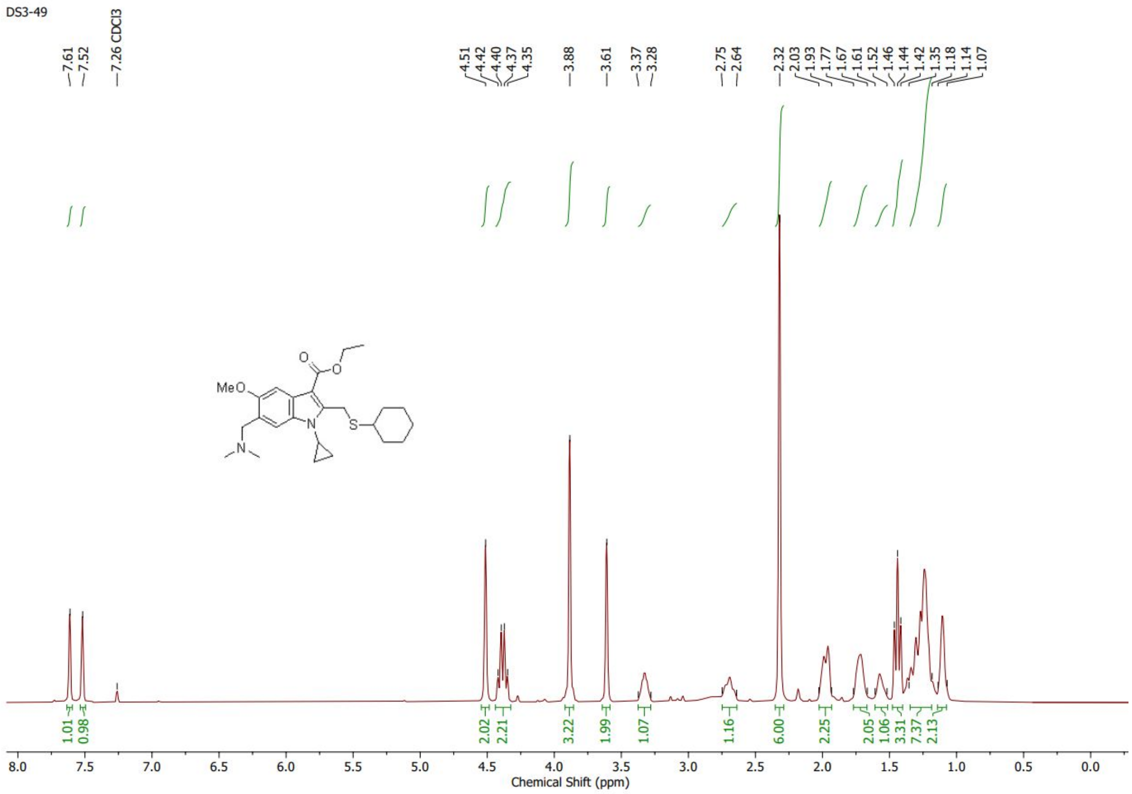

Compound 15 - Ethyl 1-(cyclopropylmethyl)-6-((dimethylamino)methyl)-5-methoxy-2-((*p*-tolylthio)methyl)-1H-indole-3-carboxylate – pale-orange solid, 40% yield.

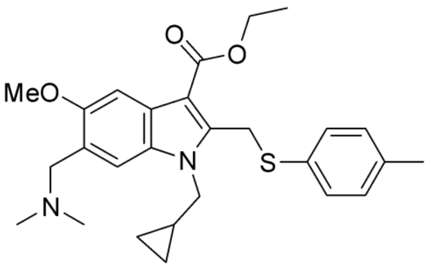

Figure S44. LC-MS ESI$^+$. t_R = 0.926 min (purity 99%); exact mass calcd for $C_{27}H_{34}N_2O_3S$: 466.22, found: *m/z* 467.2 $[M + H]^+$.

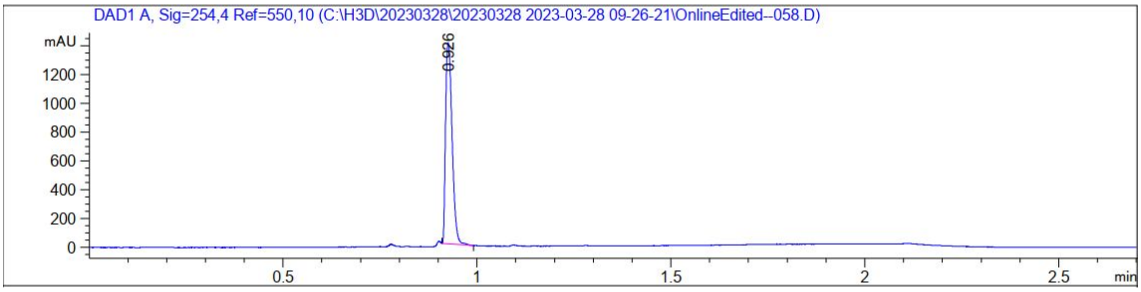

Signal 1: DAD1 A, Sig=254,4 Ref=550,10

Peak #	RetTime [min]	Type	Width [min]	Area [mAU*s]	Height [mAU]	Area %
1	0.926	BB	0.0181	1627.37085	1397.26282	100.0000

Totals :				1627.37085	1397.26282	

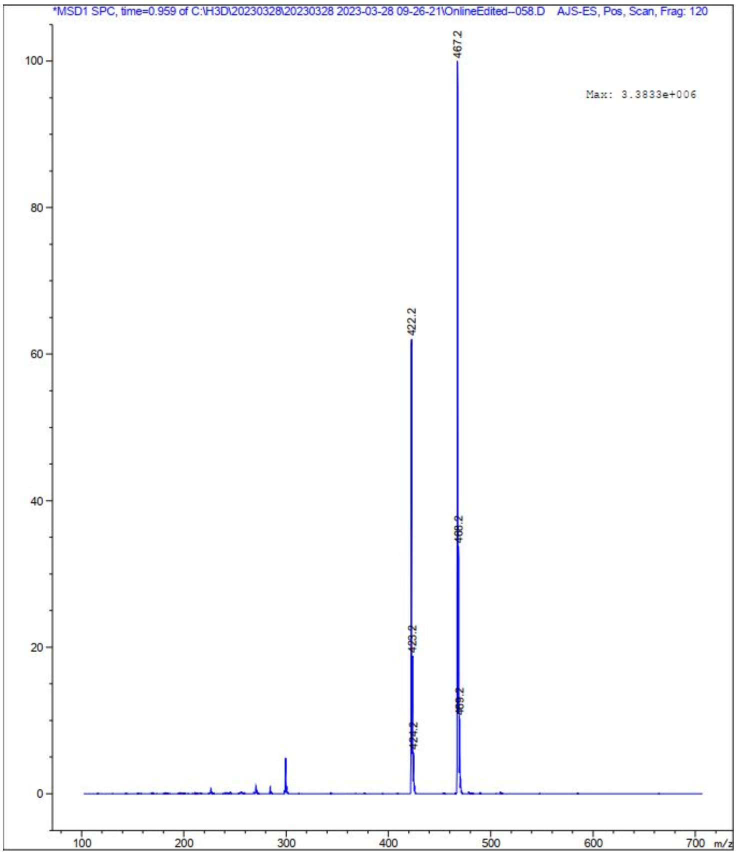

Figure S45. ^{1}H NMR (300 MHz, $CDCl_3$) δ 8.01 (s, 1H), 7.74 (s, 1H), 7.31 – 7.25 (m, 3H),* 7.09 (d, J = 7.2 Hz, 2H), 4.68 (s, 2H), 4.42 – 4.37 (m, 2H), 4.30 (q, 2H, J = 7.2 Hz), 4.07 (d, 2H, J = 7.2 Hz), 3.95 (s, 3H), 2.79 (s, 6H), 2.34 (s, 3H), 1.39 (t, 3H, J = 7.2 Hz), 1.35 – 1.29 (m, 1H), 0.61 – 0.53 (m, 2H), 0.46 – 0.39 (m, 2H).

**One extra proton due to overlap with $CDCl_3$ signal*

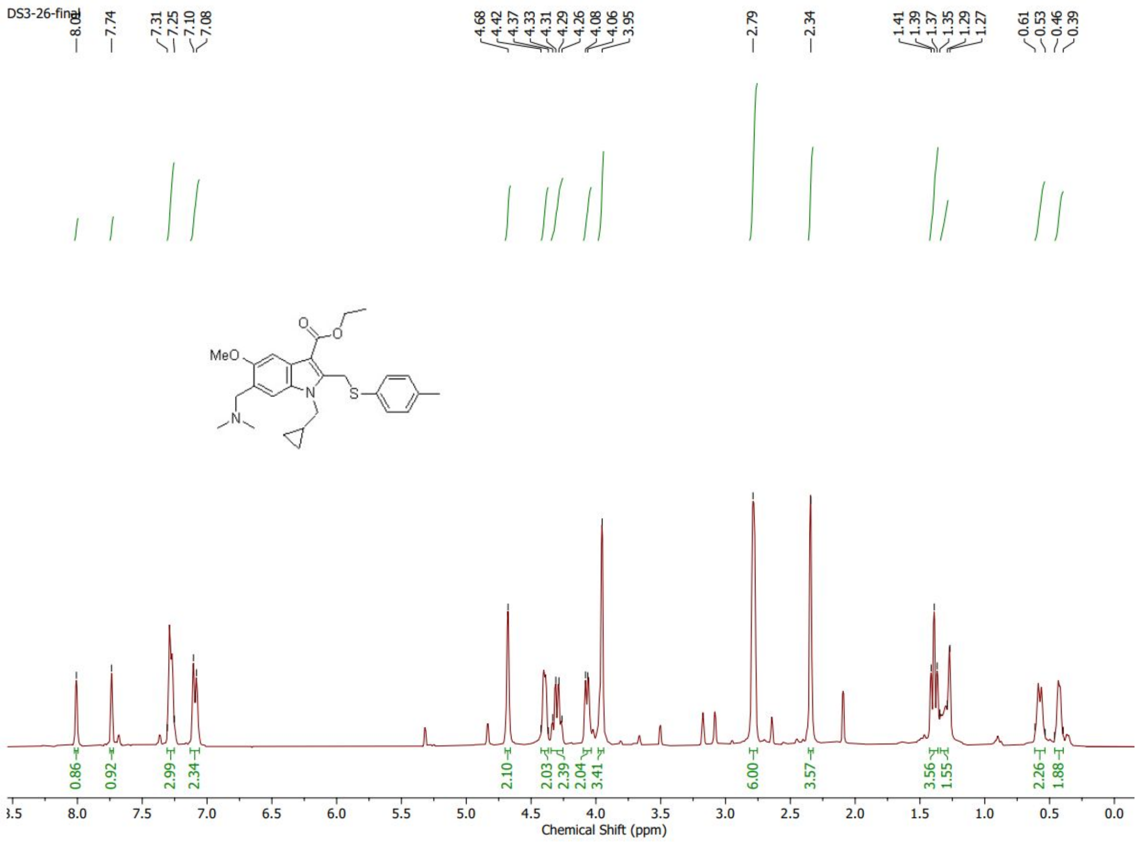

Compound 10 - Ethyl 2-((cyclohexylthio)methyl)-1-(cyclopropylmethyl)-6-((dimethylamino)methyl)-5-methoxy-1H-indole-3-carboxylate – off-white solid, 50% yield.

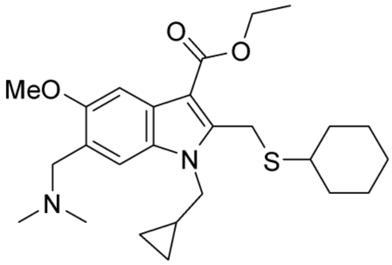

Figure S46. LC-MS ESI^{+}. t_R = 0.952 min (purity 100%); exact mass calcd for $C_{26}H_{38}N_2O_3S$: 458.26, found: *m/z* 459.3 $[M + H]^{+}$.

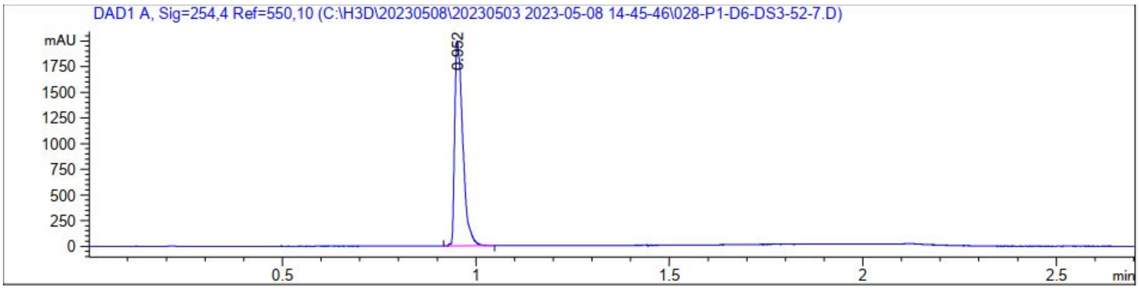

Signal 1: DAD1 A, Sig=254,4 Ref=550,10

Peak #	RetTime [min]	Type	Width [min]	Area [mAU*s]	Height [mAU]	Area %
1	0.952	BB	0.0227	2910.84937	1977.29260	100.0000
Totals :				2910.84937	1977.29260	

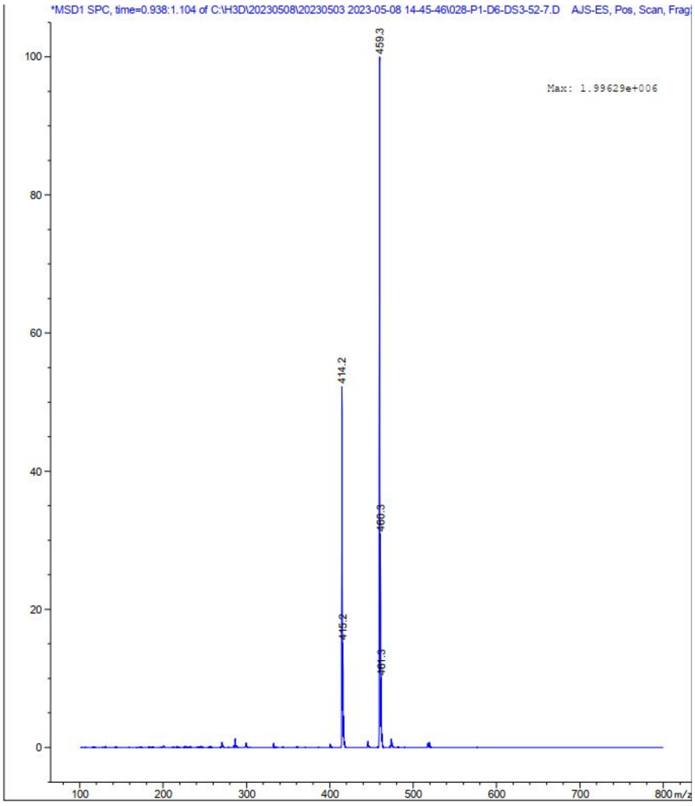

Figure S47. ^{1}H NMR (300 MHz, $CDCl_3$) δ 7.65 (s, 1H), 7.56 (s, 1H), 4.43 – 4.34 (m, 4H), 4.18 (d, *J* = 6.5 Hz, 2H), 3.93 – 3.86 (m, 5H), 2.77 – 2.68 (m, 1H), 2.48 (s, 6H), 2.01 – 1.94 (m, 2H), 1.76 – 1.66 (m, 2H), 1.44 (t, 3H, *J* = 7.1 Hz), 1.35 – 1.19 (m, 7H), 0.59 – 0.52 (m, 2H), 0.43 – 0.36 (m, 2H).

*Impurity noted at δ 1.58 ppm

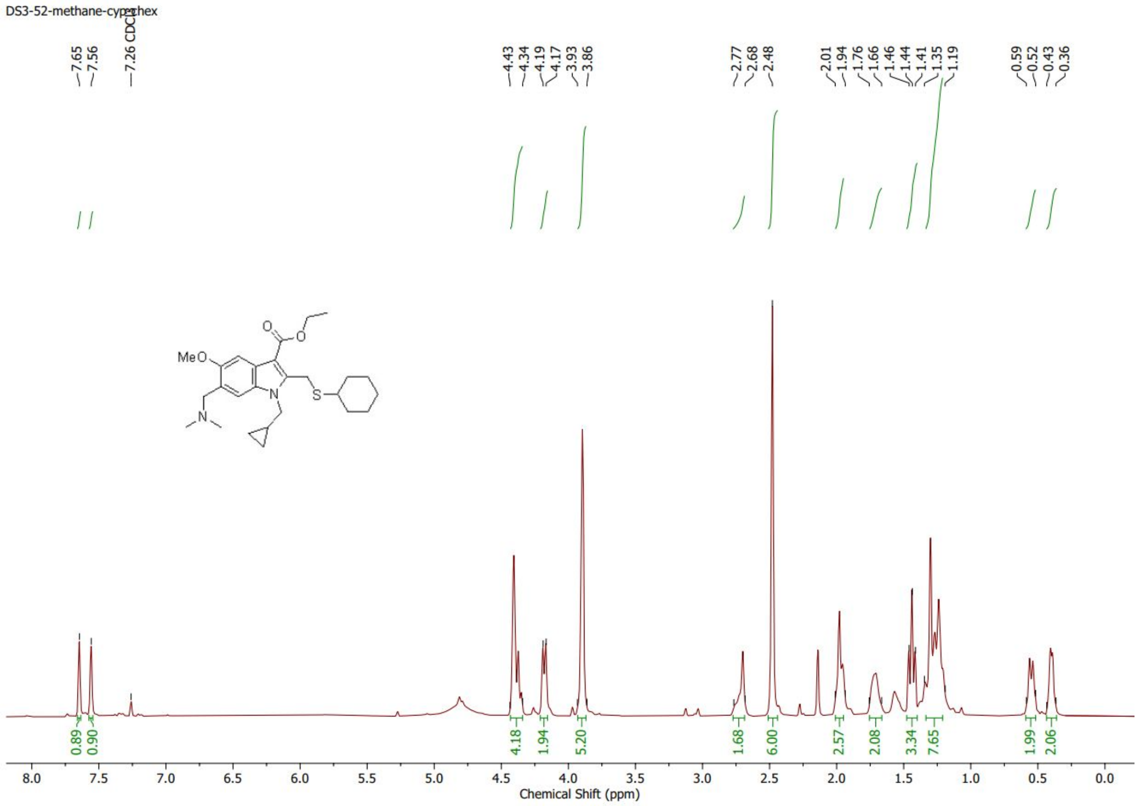

Compound 17 - Ethyl 2-(((4-chlorophenyl)thio)methyl)-1-(cyclopropylmethyl)-6-((dimethylamino)methyl)-5-methoxy-1H-indole-3-carboxylate – off-white solid, 31% yield.

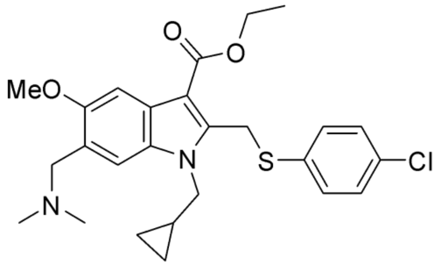

Figure S48. LC-MS ESI$^+$. t_R = 0.981 min (purity 100%); exact mass calcd for $C_{26}H_{31}ClN_2O_3S$: 486.17, found: *m/z* 487.2 $[M + H]^+$.

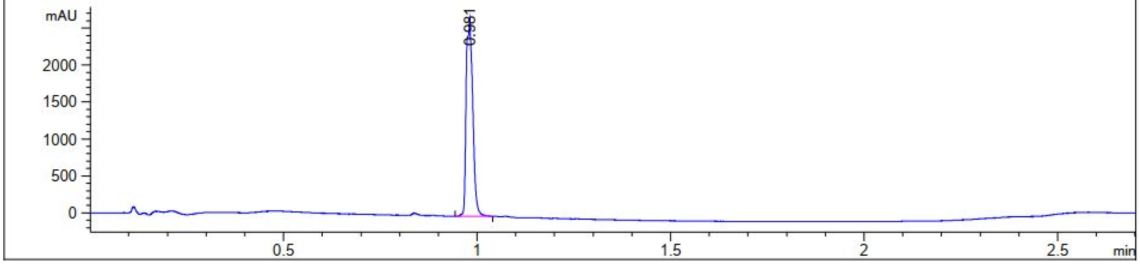

Signal 1: DAD1 A, Sig=254,4 Ref=550,10

Peak #	RetTime [min]	Type	Width [min]	Area [mAU*s]	Height [mAU]	Area %
1	0.981	BB	0.0167	3149.40161	2681.14160	100.0000

Totals :				3149.40161	2681.14160	

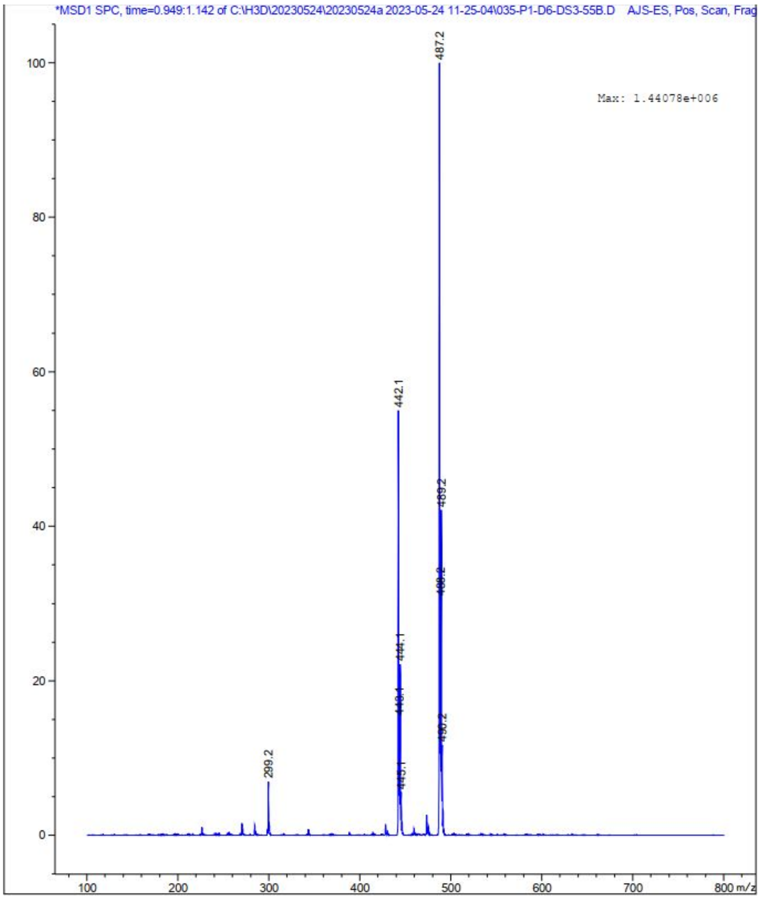

Figure S49. **^{1}H NMR (300 MHz, $CDCl_3$)** δ 7.51 (s, 1H), 7.29 (s, 1H), 7.17 (d, 2H, *J* = 8.0 Hz), 7.10 (d, 2H, *J* = 8.0 Hz), 4.62 (s, 2H), 4.17 (q, 2H, *J* = 7.1 Hz), 3.98 (d, 2H, *J* = 6.5 Hz), 3.80 (s, 3H), 3.56 (s, 2H), 2.25 (s, 6H), 1.27 (t, 3H, *J* = 7.1 Hz), 1.17 – 1.05 (m, 1H), 0.51 – 0.41 (m, 2H), 0.30 – 0.22 (m, 2H).

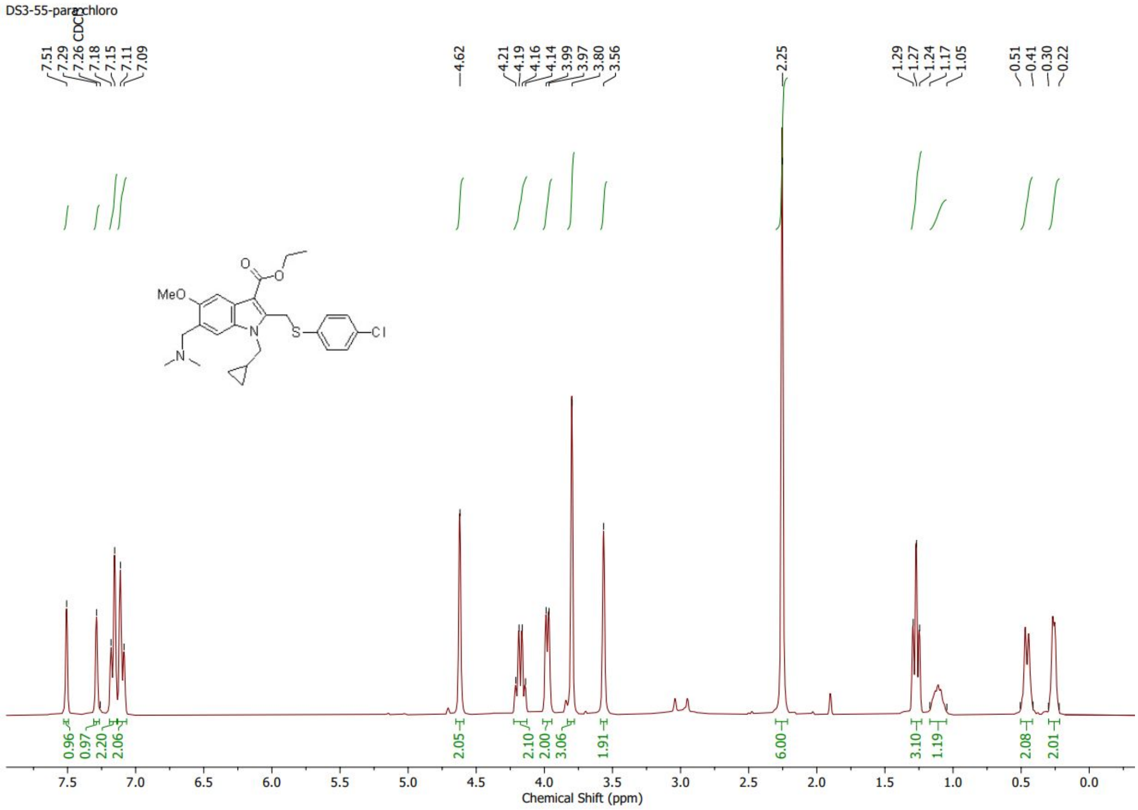

Compound 32 - Ethyl 4-((dimethylamino)methyl)-5-methoxy-1-methyl-2-((phenylthio)methyl)-1*H*-indole-3-carboxylate – brown solid, 50% yield.

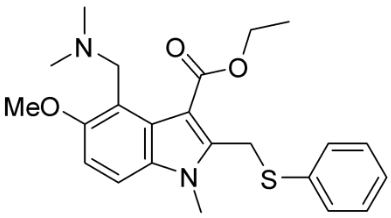

Figure S50. LC-MS ESI$^+$. t_R = 0.849 min (purity 100%); exact mass calcd for $C_{23}H_{28}N_2O_3S$: 412.18, found: *m/z* 413.2 [M +H]$^+$.

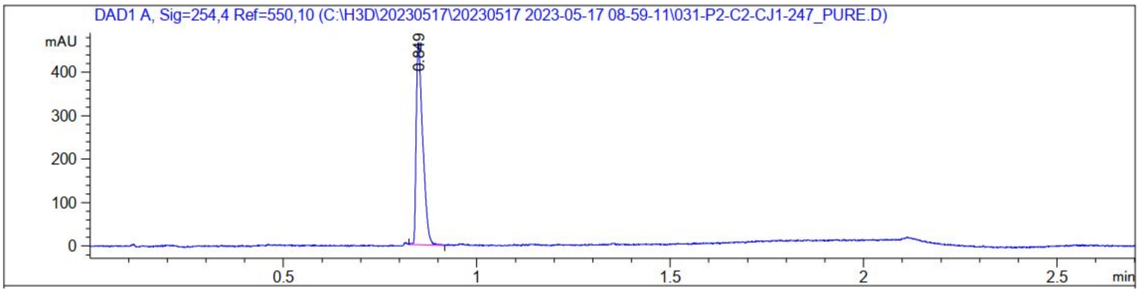

```
Signal 1: DAD1 A, Sig=254,4 Ref=550,10

Peak RetTime Type  Width     Area      Height     Area
  #   [min]        [min]   [mAU*s]     [mAU]        %
----|-------|----|-------|----------|----------|--------|
   1   0.849 BB    0.0173  547.02832  464.07932 100.0000

Totals :                   547.02832  464.07932
```

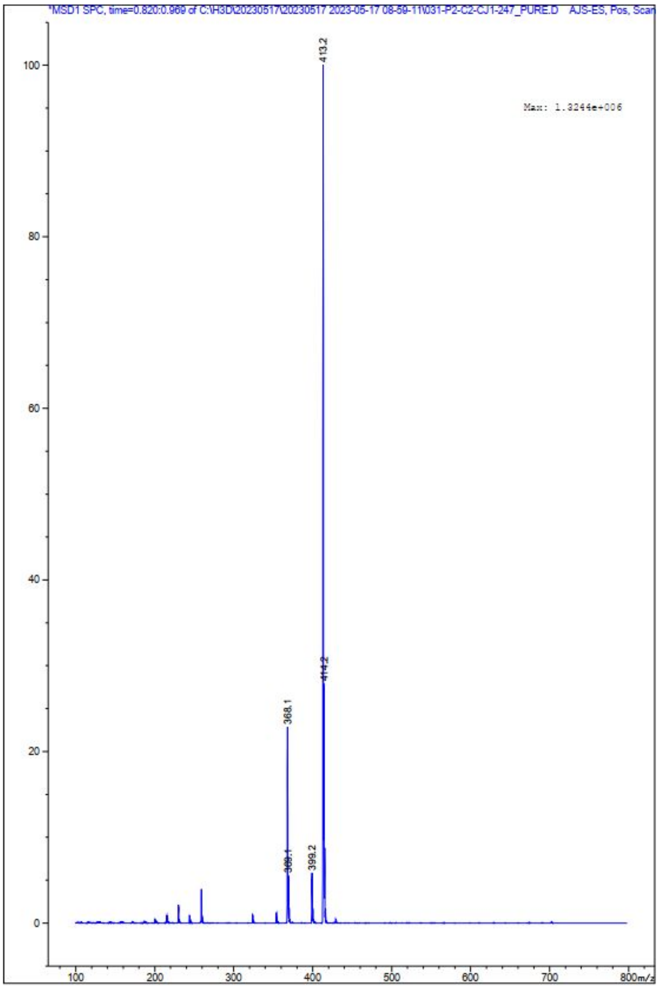

Figure S51. ^{1}H NMR (300 MHz, $CDCl_3$) δ 7.36 (d, 1H, *J* = 9.1 Hz), 7.33 – 7.21 (m, 5H), 7.07 (d, 1H, *J* = 9.1 Hz), 4.58 (s, 2H), 4.22 – 4.14 (m, 4H),* 3.86 (s, 3H), 3.63 (s, 3H), 2.17 (s, 6H), 1.30 (t, 3H, *J* = 7.1 Hz).

* The quartet of the ester and the singlet of the Mannich methylene protons are overlapping to produce a multiplet that integrates for 4 protons.

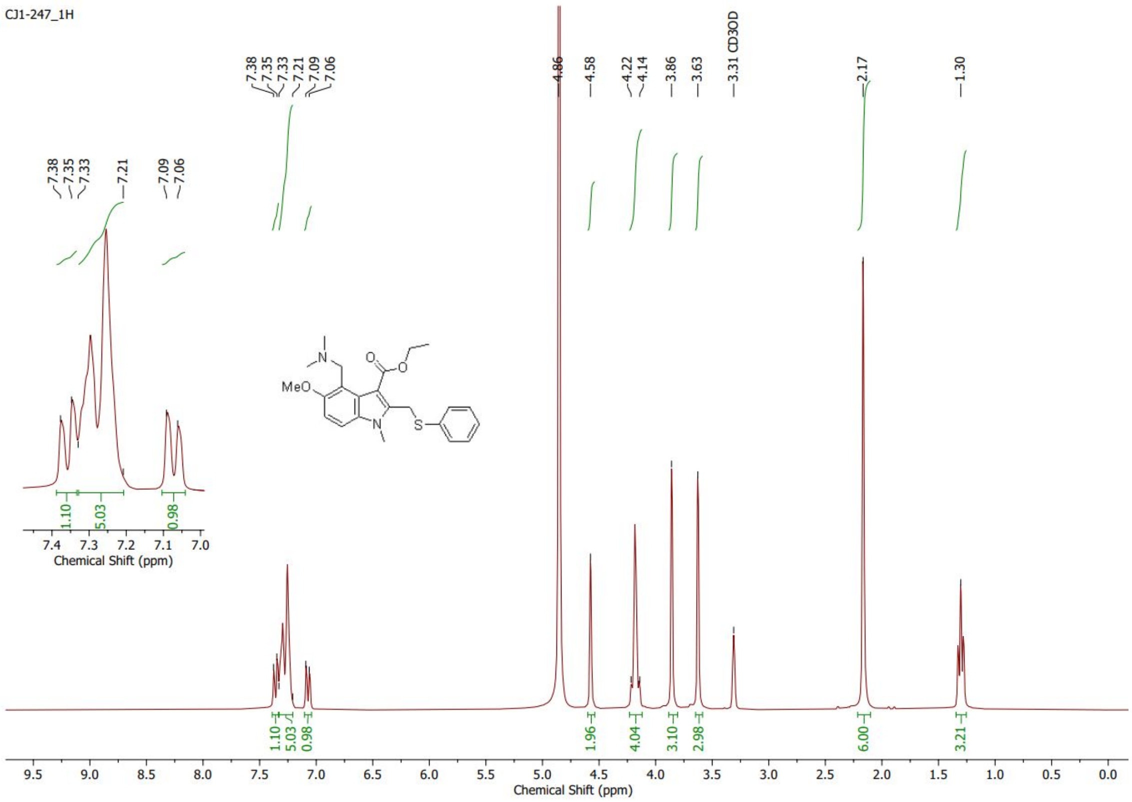

Compound 33 - Ethyl 1-(cyclopropylmethyl)-4-((dimethylamino)methyl)-5-methoxy-2-((p-tolylthio)methyl)-1H-indole-3-carboxylate – off-white solid, 31% yield.

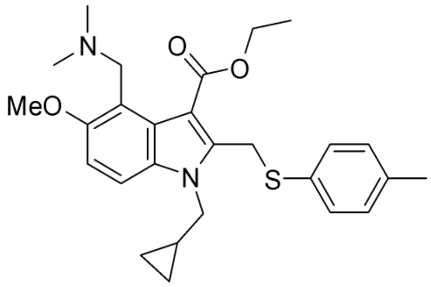

Figure S52. LC-MS ESI$^+$: t_R = 0.949 min (purity 98%); exact mass calcd for $C_{27}H_{34}N_2O_3S$: 466.22, found: *m/z* 467.2 [M + H]$^+$.

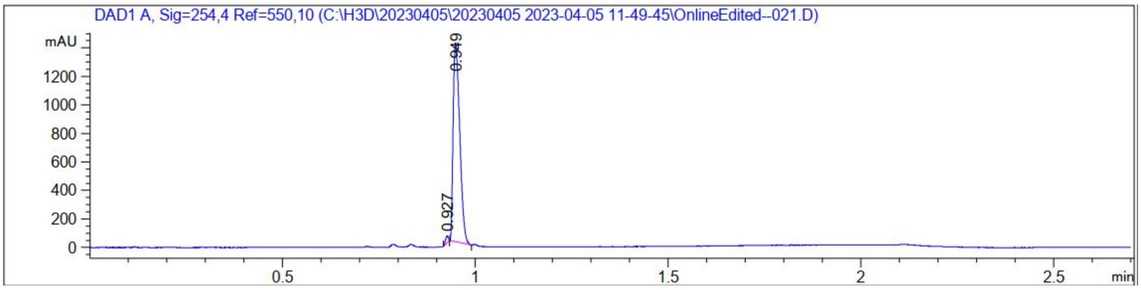

Signal 1: DAD1 A, Sig=254,4 Ref=550,10

Peak #	RetTime [min]	Type	Width [min]	Area [mAU*s]	Height [mAU]	Area %
1	0.927	BB	8.50e-3	24.22588	47.06968	1.4440
2	0.949	BB	0.0189	1653.50281	1392.47241	98.5560
Totals :				1677.72869	1439.54209	

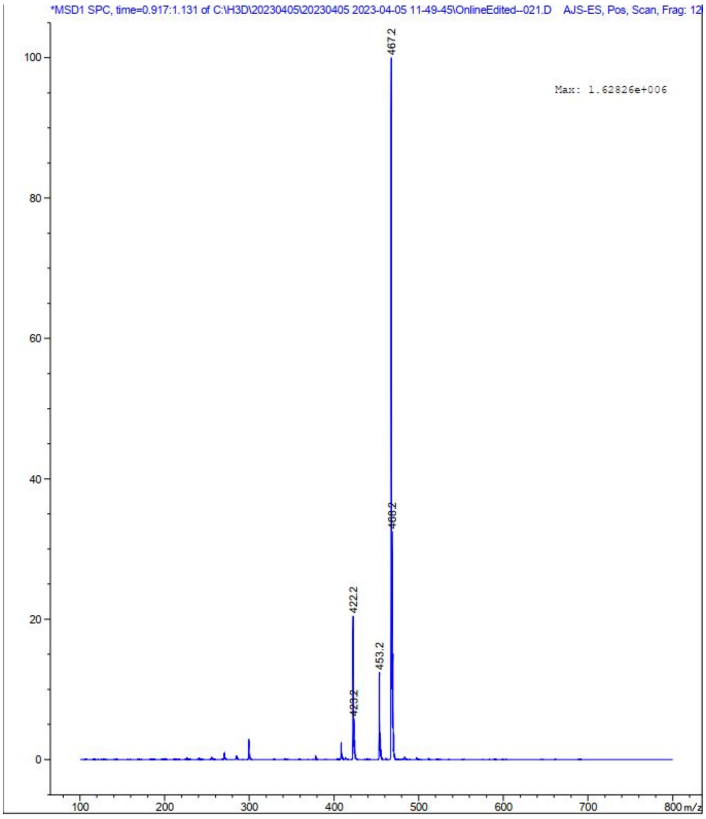

Figure S53. ^{1}H NMR (300 MHz, DMSO-*d6*) δ 7.44 (d, 1H, *J* = 8.8 Hz), 7.26 (d, 2H, *J* = 7.5 Hz), 7.14 (d, 2H, *J* = 7.5 Hz), 7.02 (d, 1H, *J* = 8.8 Hz), 4.54 (s, 2H), 4.16 – 4.05 (m, 4H), 3.80 – 3.71 (m, 5H), 2.28 (s, 3H), 1.91 (s, 6H), 1.28 – 1.15 (m, 4H), 0.50 – 0.38 (m, 4H).

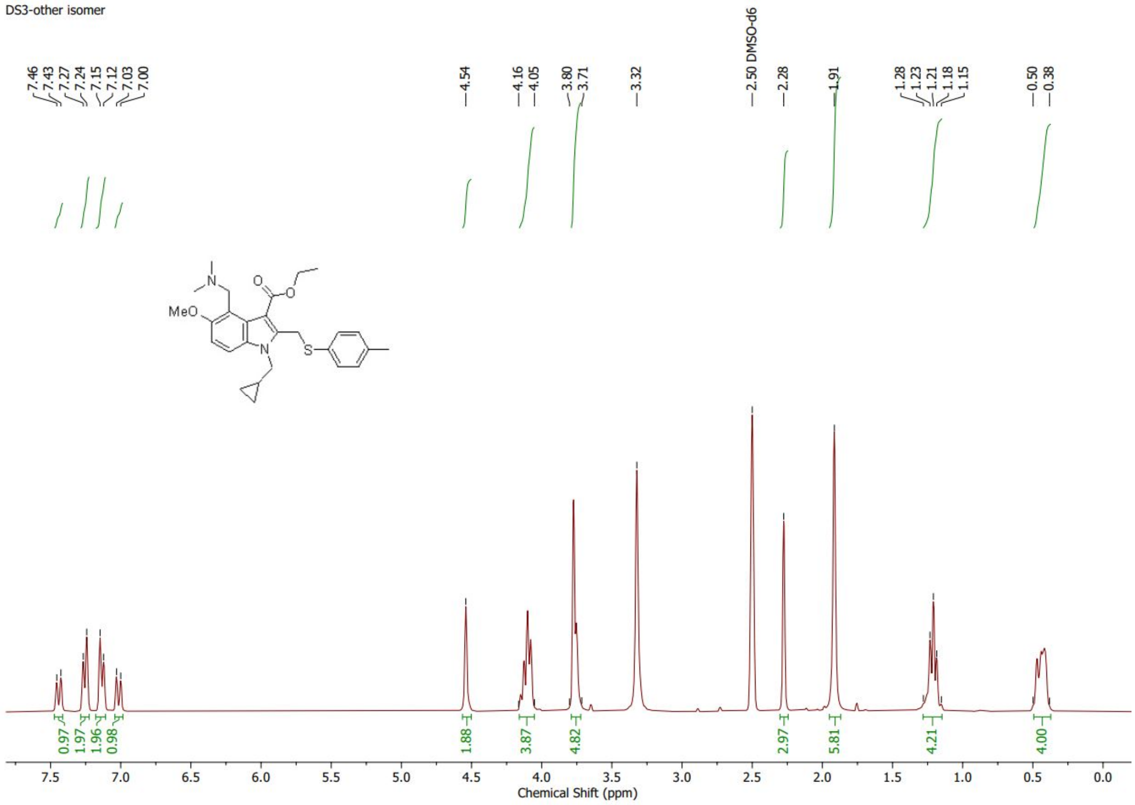

Compound 29 - Ethyl 1-(cyclopropylmethyl)-6-((dimethylamino)methyl)-2-(((4'-fluoro-[1,1'-biphenyl]-4-yl)thio)methyl)-5-methoxy-1H-indole-3-carboxylate – off-white solid, 36% yield.

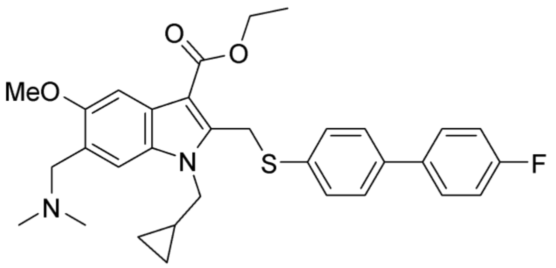

Figure S54. LC-MS ESI$^+$. t_R = 0.993 min (purity 100%); exact mass calcd for $C_{32}H_{35}FN_2O_3S$: 546.23, found: *m/z* 547.2 $[M + H]^+$.

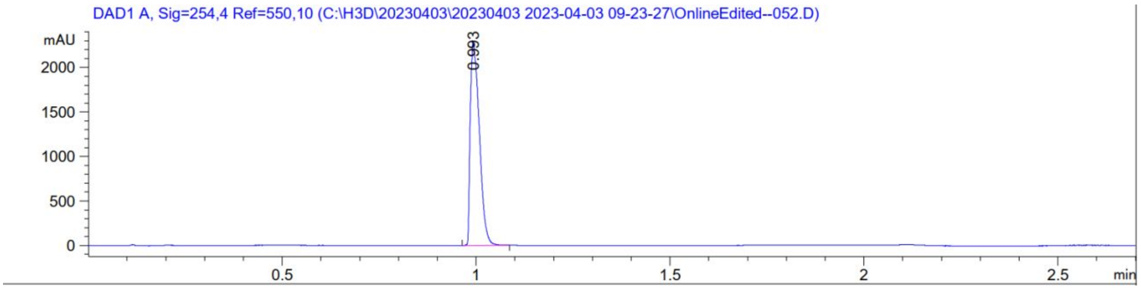

Signal 1: DAD1 A, Sig=254,4 Ref=550,10

Peak #	RetTime [min]	Type	Width [min]	Area [mAU*s]	Height [mAU]	Area %
1	0.993	BB	0.0252	3727.04785	2260.20703	100.0000

Totals : 3727.04785 2260.20703

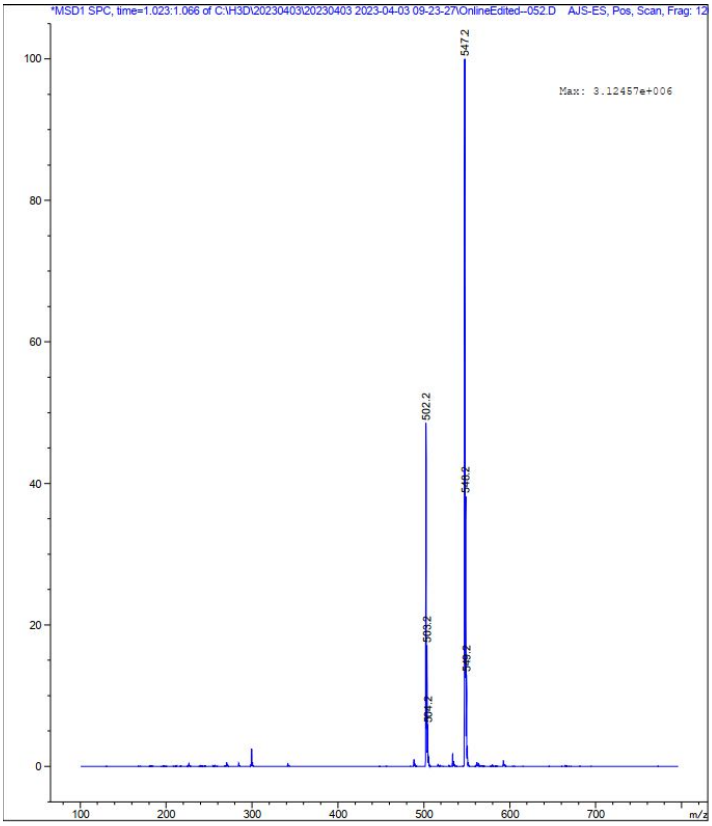

Figure S55. ^{1}H NMR (300 MHz, DMSO) δ 7.74 – 7.65 (m, 2H), 7.60 (d, *J* = 7.6 Hz, 2H), 7.51 – 7.42 (m, 4H), 7.33 – 7.24 (m, 2H), 5.75 (s, 2H), 4.87 (s, 2H), 4.26 – 4.12 (m, 4H), 3.80 (s, 3H), 3.50 (s, 2H), 2.20 (s, 6H), 1.35 – 1.18 (m, 4H), 0.52 – 0.40 (m, 4H).

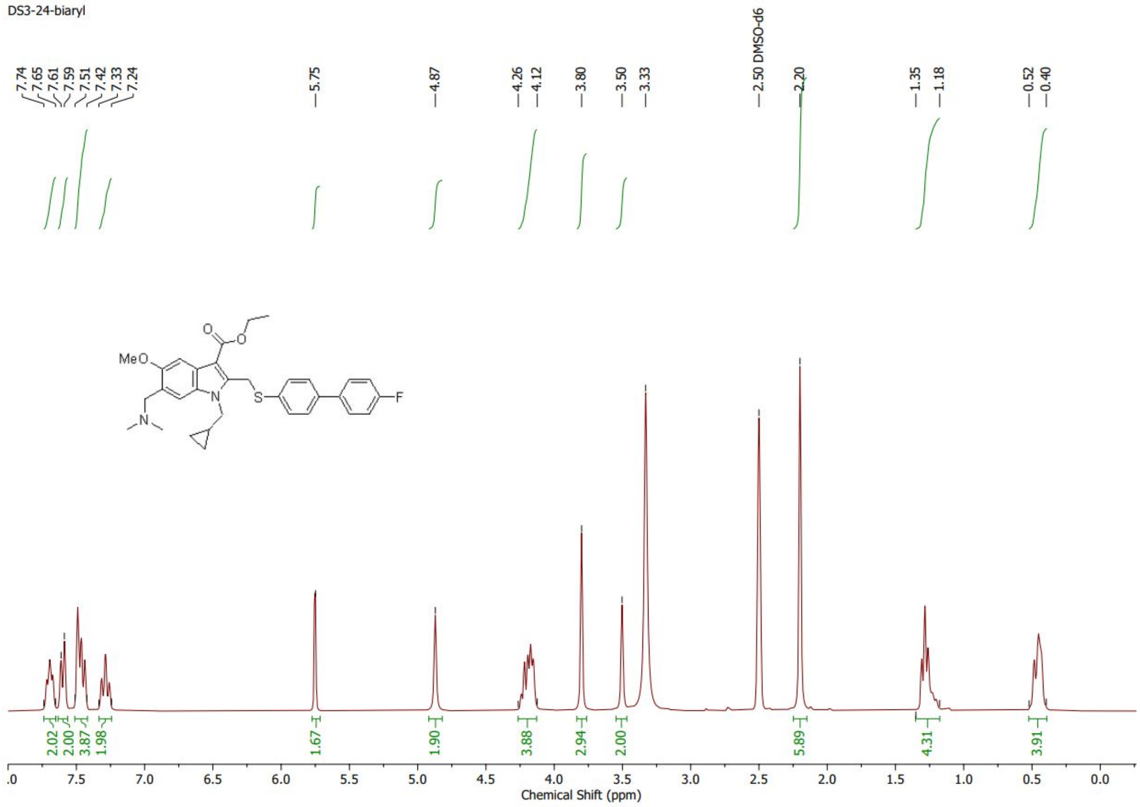

Compound 31 - Ethyl 1-(cyclopropylmethyl)-6-((dimethylamino)methyl)-5-methoxy-2-(((4-(6-(trifluoromethyl)pyridin-3-yl)phenyl)thio)methyl)-1H-indole-3-carboxylate – yellow solid, 53% yield.

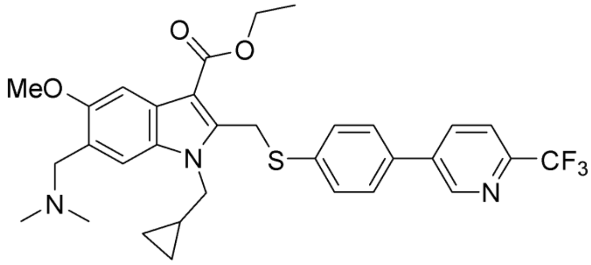

Figure S56. LC-MS ESI$^+$. t_R = 0.981 min (purity 99%); exact mass calcd for $C_{32}H_{34}F_3N_3O_3S$: 597.22, found: *m/z* 598.2 $[M + H]^+$.

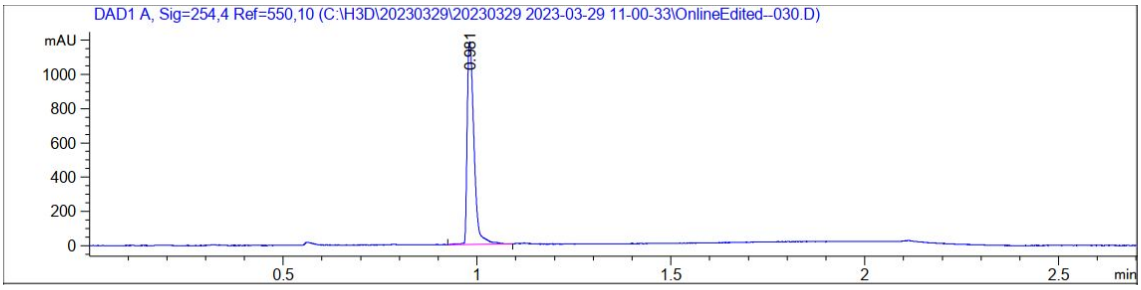

Signal 1: DAD1 A, Sig=254,4 Ref=550,10

Peak #	RetTime [min]	Type	Width [min]	Area [mAU*s]	Height [mAU]	Area %
1	0.981	BB	0.0192	1441.28259	1182.66113	100.0000
Totals :				1441.28259	1182.66113	

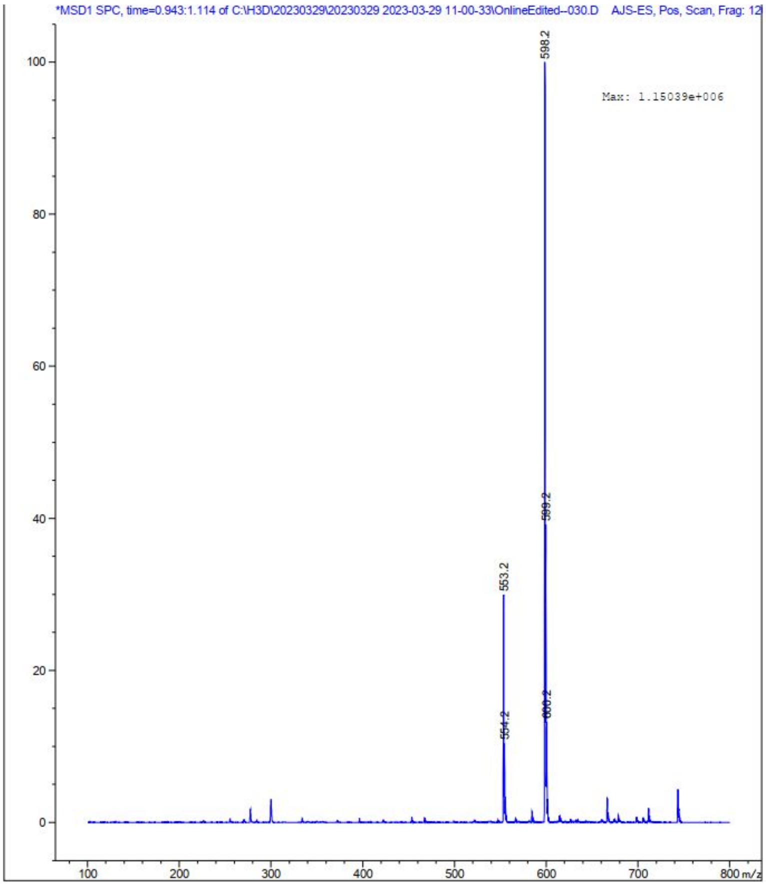

Figure S57. ^{1}H NMR (300 MHz, $CDCl_3$) δ 8.91 (s, 1H), 8.02 (d, 1H, *J* = 8.1 Hz), 7.75 (d, 1H, *J* = 7.5 Hz), 7.66 – 7.60 (m, 2H), 7.53 – 7.48 (m, 4H), 4.84 (s, 2H), 4.30 (q, 2H, *J* = 7.1 Hz), 4.16 (d, 2H, *J* = 6.0 Hz), 3.96 – 3.86 (m, 5H), 2.48 (s, 6H), 1.37 (t, 3H, *J* = 7.1 Hz), 1.29 – 1.20 (m, 1H), 0.63 – 0.54 (m, 2H), 0.45 – 0.37 (m, 2H).

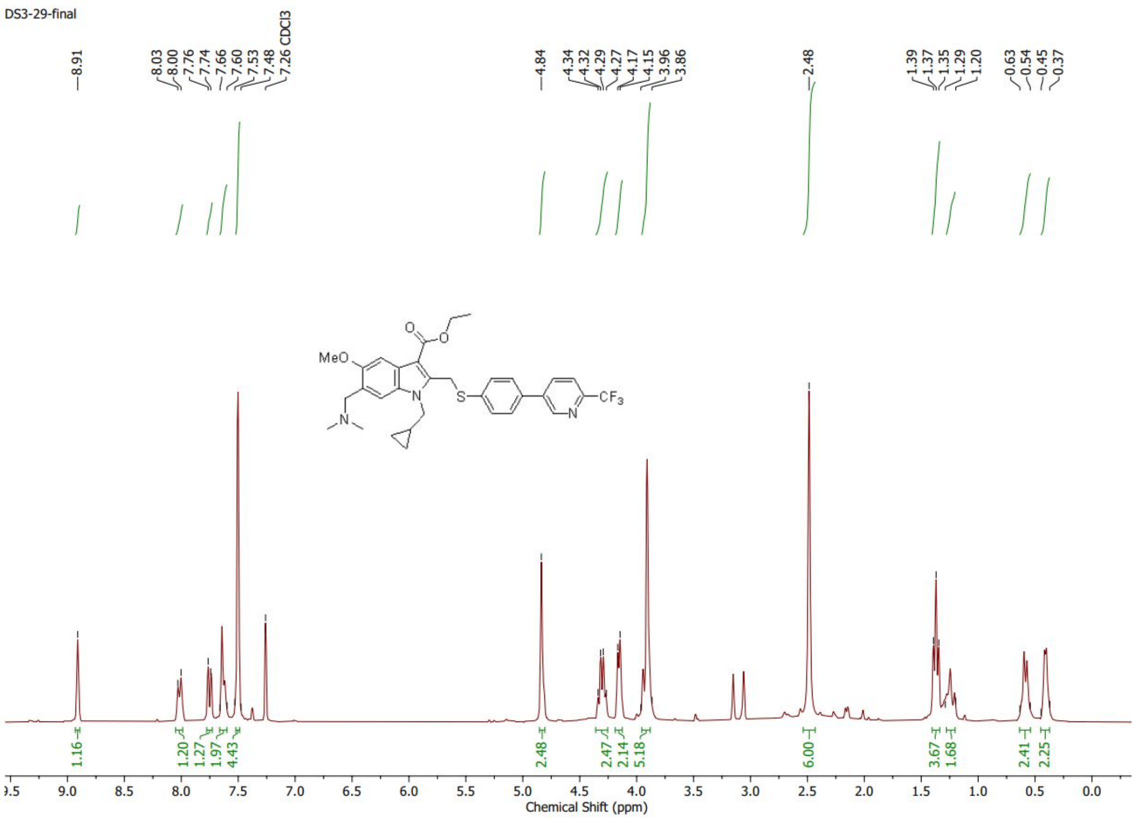

2. Supplementary Tables

Antiviral Assays Against Delta and Omicron (XBB.1.5 Lineage) Variants

For the most promising compounds from the first round, we also performed S-fuse assays against Delta and Omicron (XBB1.5 lineage) variants (Table S1).

Table S1. Compounds antiviral activity against Delta and Omicron variant (XBB1.5 lineage) and cytotoxicity

Compound ID	Chemical Structure	S-Fuse Delta IC_{50}(uM)	S-Fuse Delta CC_{50}(uM)	S-Fuse XBB1.5 IC_{50}(uM)	S-Fuse XBB1.5 CC_{50}(uM)
**1**	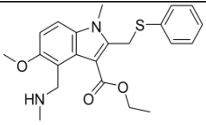			2.34	>50
**2**	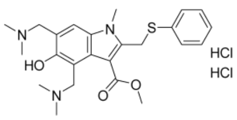	0.5	1.33		

**3**	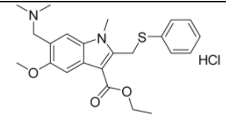	0.41	32.02		
**4**	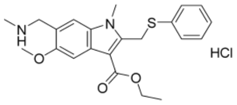			1.69	26.5

For Delta variant, **3** maintained similar activity across the Delta variant (IC_{50} = 0.41 µM), showing promising cross-variant efficacy with a CC_{50} of 32 µM, indicating favorable selectivity. **2** exhibited an EC_{50} value of 0.5 µM and a CC_{50} of 1.3 µM against the Delta variant. Combined with its previously reported activity against the Wuhan variant (EC_{50} = 3.30 µM) and the Omicron BA.2.86.1 variant (EC_{50} = 1.21 µM), these results highlight its potential efficacy across multiple SARS-CoV-2 lineages.

For omicron variant (XBB1.5 lineage) compounds **4** and **1** displayed EC_{50} values of 1.69 µM and 2.34 µM, respectively, with CC_{50} values of 26.5 µM and > 50 µM. The resulting SIs of 16 and > 20 for **4** and **1**, respectively, indicate favorable selectivity for these compounds, making them promising candidates for further development across variants.

4 showed consistent antiviral potency with an EC_{50} of 1.69 µM and a CC_{50} of 26.5 µM against the Delta and Wuhan variants, yielding a favorable selectivity index. Against the Omicron BA.2.86.1 variant, it exhibited slightly reduced potency (EC_{50} = 1.92 µM) but maintained a high CC_{50} value (>50 µM), indicating a safety margin. Against the emerging XBB.1.5 variant, its performance paralleled that seen in Delta and Wuhan, with an IC_{50} of 1.69 µM and a CC_{50} of 26.5 µM.

1, while slightly less potent than 12226119, demonstrated an IC_{50} of 2.34 µM and a CC_{50} of 32.37 µM against the Wuhan variant. Against the Omicron BA.2.86.1 variant, its IC_{50} increased to 5.21 µM, and a reduced CC_{50} of 21.13 µM, indicating somewhat reduced efficacy and selectivity. Against the Delta variant, the compound retained its IC_{50} of 2.34 µM, but its CC_{50} for the XBB.1.5 variant improved significantly (>50 µM), suggesting reduced cytotoxicity against this particular lineage.

In summary, integrating the results from all SARS-CoV-2 variants, compounds **4** and **1** show high selectivity indexes, suggesting potent antiviral effects combined with low cytotoxicity across all SARS-CoV-2 variants.

SARS-CoV-2 Machine Learning Model Cross-validation – used in MegaSyn

Table S2. 5-fold cross-validation statistics for SARS-CoV-2 models. Adaboost (ada), Naïve Bayes (bnn), k-Nearest Neighbors (knn), Logistic Regression (lreg), Random Forest (rf), XGBoost (xgb) and Support Vector Machines (svc).

Model	ACC	F1-Score	AUC	Cohen's Kappa	MCC	Precision	Recall	Specificity
Lreg	0.86	0.31	0.72	0.25	0.27	0.50	0.24	0.96
ada	0.85	0.00	0.67	-0.01	-0.02	0.00	0.00	0.99
rf	0.87	0.24	0.73	0.20	0.23	0.47	0.17	0.98
xgb	0.85	0.19	0.71	0.13	0.16	0.41	0.12	0.97
knn	0.84	0.40	0.66	0.31	0.31	0.42	0.39	0.91
svc	0.70	0.39	0.23	0.24	0.27	0.33	0.58	0.72
bnn	0.86	0.31	0.75	0.25	0.26	0.43	0.25	0.96

3. Supplementary Schemes

Chemical Synthesis - Materials and Methods for Chemical Synthesis Performed at the Research Center of Biotechnology (RAS).

All reagents and solvents were purchased from commercial suppliers and used without further purification. ^{1}H and ^{13}C Spectra were measured on Bruker AC-500 (500 MHz, ^{1}H) or Bruker AC-200 (75 MHz, ^{13}C). Chemical shifts were measured in DMSO-d6 or CDCl3, using tetramethylsilane as an internal standard, and reported as units (ppm) values. The following abbreviations indicate the multiplicity: s, singlet; d, doublet; t, triplet; m, multiplet; dd, doublet of doublets; brs, broad singlet; brm, broad multiplet.

HRMS: spectra were recorded on an Agilent 1290 Infinity II HPLC system coupled to an Agilent 6460 triple-quadrupole HRMS: spectrometer with an electrospray ionization source. The chromatographic separation was carried out on Agilent Eclipse Plus C18 RRHD column (2.1 × 50 mm, 1.8 μm) at 40 ºC with sample injection volume of 0.2 μL. The mobile phase comprising 0.1 % formic acid / water (A), and 0.1 % formic acid and 85 % acetonitrile / water (B) was programmed to do a gradient elution (0.0-3.0 min, 60 % B; 3.0-4.0 min, 60 % to 97 % B; 4.0-6.0 min, 97 % B; 6.0-6.1 min, 97 % to 60 % B) at a flow rate of 0.4 mL/min. The HRMS: spectrometric detection was operated in

a positive ion mode. Optimal parameters were capillary voltages of 3500 V, a nebulizer pressure of 35 psi, a gas temperature of 350 ºC, a gas flow rate of 12 L/min.

Purity was measured by analytical high-performance liquid chromatography (HPLC) on an Elute HPLC system (Bruker Daltonik) equipped with Azura UVD 2.1S UV detector (Knauer) using Acquity HSS T3 column (2.1 × 100 mm, 1.3 µm, 100 Å) at 30 °C, 2 µL injection, 250 µL/min gradient elution 30─95% B (A: 0.1% FA in H_2O, B: 0.1% FA in MeCN) over 9 min with 1 min gradient delay, 1 Hz acquisition rate at 254 nm. Data were processed with Compass DataAnalysis 5.1 (Bruker Daltonik). Purity is > 95% of all final compounds.

Melting points were determined by Electrothermal 9001 (10 ºC per min) and are uncorrected. Merck KGaA silica gel 60 F254 plates were used for analytical thin-layer chromatography. Column chromatography was performed on Merck silica gel 60 (70-230 mesh). Yields refer to purified products and are not optimized. The starting materials and the intermediates of the synthetic reaction schemes were also isolated and purified as desired using conventional techniques, including but not limited to, filtration, distillation, crystallization, chromatography, etc. Such materials were characterized using conventional means, including physical constants and spectral data.

Materials and Methods for Chemical Synthesis Performed at the Holistic Drug Discovery and Development Centre (H3D).

All commercially available chemicals were purchased from either Sigma-Aldrich (South Africa) or Combi-Blocks Limited (USA). These chemicals were of analytical grade and used without further purification. Reactions were monitored by HPLC-MS and thin-layer chromatography (TLC) using Fluka or Merck F254 aluminium-backed precoated silica gel plates and were visualized under ultraviolet light at 254 or 366 nm.

Silica gel flash column chromatography and reverse-phase flash column chromatography were performed using a Teledyne ISCO Combi*Flash* Rf$^+$ system (Teledyne ISCO, Lincoln, USA). Preparative high-performance liquid chromatography was performed using a Teledyne ACCQPrep HP150 system. The mobile phase solvents for column chromatography were purchased as analytical reagent (AR) grade and used without prior distillation.

Final compounds were characterized via ^{1}H NMR spectroscopy and high-performance liquid chromatography-mass spectrometry (HPLC-MS). ^{1}H NMR spectra

were recorded on Varian Mercury (300 MHz), Bruker Ultrashield-Plus (400 MHz), or a Bruker Ascend™ (600 MHz) instrument. Samples were dissolved in deuterated dimethyl sulfoxide (DMSO-*d*6), methanol (MeOH-*d*4) or chloroform ($CDCl_3$). Chemical shifts (δ) are reported in parts per million (ppm) and rounded off to two decimal places. Coupling constants (*J*) are reported in hertz (Hz) and rounded to one decimal place. Abbreviations used in assigning ^{1}H NMR signals are d (doublet), dd (doublet of doublets), ddd (doublet of doublet of doublets), m (multiplet), q (quartet), s (singlet), br. s (broad singlet), t (triplet), or td (triplet of doublets).

Purity was determined using an Agilent 1290 Infinity binary pump, Agilent 1290 Infinity diode array detector (DAD), Agilent 1290 Infinity column compartment, Agilent 1290 Infinity standard autosampler, and Agilent 6150 quadrupole (single) mass spectrometer, equipped with ESI source. All compounds tested for biological activity were confirmed to have ≥ 95% purity. LC purity analyses were performed using the following method:

Kinetex 1.7 μm EVO C-18 column (50 x 2.1 mm, 100 Å), 1 μL injection volume, flow 1.2 mL/min; gradient: 5–100% B in 1.5 min (hold 0.4 min), 100-5% in 0.3 min (hold 0.5 min) (Mobile phase A: 0.1% formic acid in H_2O and Mobile phase B: 0.1 % formic acid in Acetonitrile);

All synthesized intermediates, except enaminones, were characterized by LC-MS, while final compounds were confirmed by LC-MS and ^{1}H NMR spectroscopy.

Synthesis and characterization of compounds with R1, R2, and R3 modifications

The starting materials for the synthesis of compounds with R1, R2, and R3 modifications, 1-methyl-2-phenylmercaptomethyl-3-carbethoxy-5-methoxyindole and 1-methyl-2-phenylmercaptomethyl-3-carbethoxy-5-hydroxyindole, were synthesized according to the procedures reported by Trofimov et al[2]. All these procedures for synthesizing and characterizing compounds with modifications to the R1, R2 and R3 groups, were performed at the Research Center of Biotechnology, in Moscow, Russia.

Scheme S1

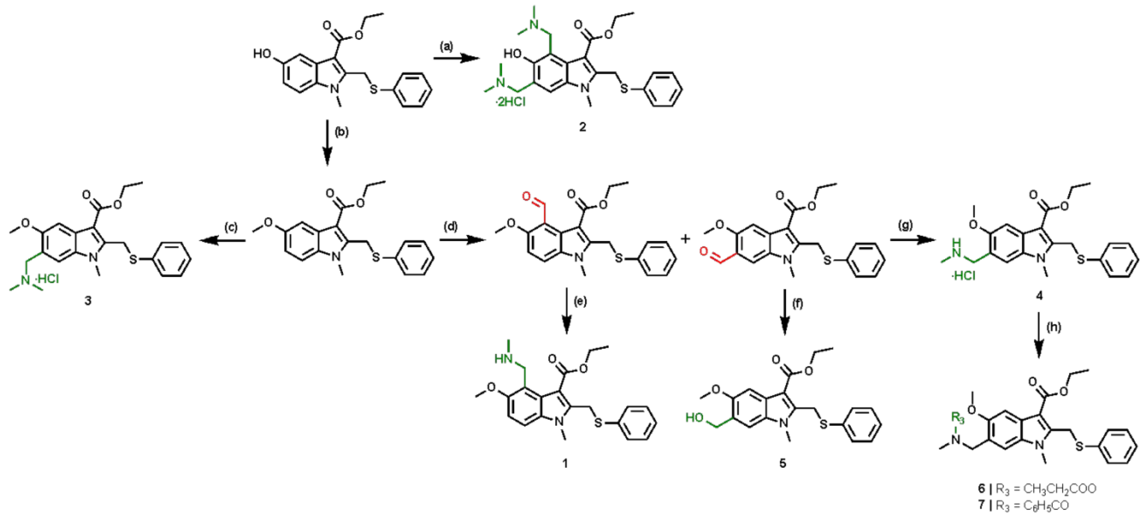

Reagents and conditions: (a) 1) 40% Me_2NH (aq.), 38% HCOH (aq.), AcOH, 70 °C, 7 h, 2) HCl/EtOH; **(b)** Trofimov *et al.* 1969; **(c)** 1) $Me_2NCH_2NMe_2$, AcOH, 85 °C, 6 h, 2) HCl/EtOH; **(d)** $POCl_3$, DMF, reflux, 6 h; **(e)** 1) 33% $MeNH_2$, EtOH, $NaBH_4$, rt, 1.5 h, 2) HCl/EtOH; **(f)** $NaBH_4$, EtOH, reflux, 5 min, then rt, 20 min; **(g)** 1) 33% $MeNH_2$, EtOH, $NaBH_4$, 50 °C → rt, 15 min, 2) HCl/EtOH; **(h)** RCOCl, Et_3N, CH_2Cl_2, 0 °C → rt, 30 min.

Synthesis of 1-methyl-2-phenylmercaptomethyl-3-carbethoxy-5-methoxy-6-dimethylaminomethylindole hydrochloride (compound 3)

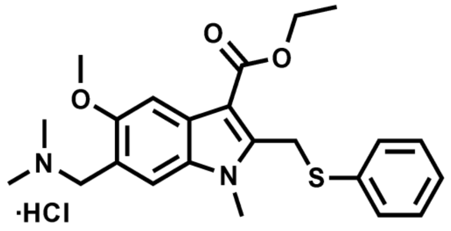

A mixture of 1-methyl-2-phenylmercaptomethyl-3-carbethoxy-5-methoxyindole (178 mg, 0.50 mmol) and bis(dimethylamino)methane (77 mg, 0.75 mmol) in acetic acid (1.5 mL) was stirred at 85 °C for 1.5 h. The reaction mixture was then cooled to ~30 °C, and an additional 1.5 eqv of bis(dimethylamino)methane was added followed by stirring at 85 °C for another 1.5 h. This procedure was repeated three times. The reaction mixture was evaporated in vacuo. Water (2 mL) and activated carbon were added to the residue, and the mixture was stirred for 4 h and filtered. To the filtrate was added 10% aqueous KOH solution up to pH ~12. The organic phase was extracted with ethyl acetate (2×20 mL), the extracts were combined, washed with water (3×10 mL) and dried over Na_2SO_4. The combined organic phase was evaporated in vacuo, and the residue was treated with ether-hexane (1:3) mixture. To a solution of the residue (75 mg) in ethanol (2 mL), a

hydrogen chloride/ethanol solution was added to adjust pH ~3, and the resulting mixture was stirred at room temperature (rt) for 1 h. The mixture was then evaporated in vacuo, and the residue was treated with ether-acetone (5:1) mixture. The precipitate was filtered, washed with ether, and air-dried. Yield 75 mg (33 %) of **3**, mp. 146-8 °C. ^{1}H NMR (200 MHz; DMSO-d$_6$; δ, ppm): 1.30 (t, *J* = 7.0 Hz, 3H, OCH$_2$C$\underline{H}_3$), 2.73 (s, 6H, N(CH$_3$)$_2$), 3.74 (s, 3H, NCH$_3$), 3.88 (s, 3H, OCH$_3$), 4.21 (q, *J* = 7.2 Hz, 2H, OC$\underline{H}_2$CH$_3$), 4.34 (s, 2H, NCH$_2$), 4.85 (s, 2H, SCH$_2$), 7.25-7.44 (m, 5H, Ph), 7.58 (s, 1H, HC(4)), 7.74 (s, 1H, HC(7)), 9.7 (s, 1H, NH). ^{13}C NMR (50 MHz; DMSO-d$_6$; δ, ppm): 14.15 (OCH$_2\underline{C}$H$_3$), 28.17 (SCH$_2$), 30.23 (NCH$_3$), 41.95 (N(CH$_3$)$_2$), 55.59 (NCH$_2$), 55.66 (OCH$_3$), 59.24 (O$\underline{C}$H$_2$CH$_3$), 101.63 (C(4)), 103.75 (C(3)), 114.33 (C(7)), 114.79 (C(6)), 127.22 (C(4')), 127.49 (C(3a)), 128.97 (C(3', 5')), 130.77 (C(7a)), 130.92 (C(2', 6')), 134.18 (C(1')), 143.89 (C(2)), 153.60 (C(5)), 164.14 (C=O). HRMS (ESI): calcd for $C_{23}H_{28}N_2O_3S$ ([M+H]$^+$) 413.1893; found 413.1896.

Synthesis of intermediate 1-methyl-2-phenylmercaptomethyl-3-carbethoxy-5-methoxy-6-formylindole and 1-methyl-2-phenylmercaptomethyl-3-carbethoxy-4-formyl-5-methoxyindole

To a suspension of 1-methyl-2-phenylmercaptomethyl-3-carbethoxy-5-methoxyindole (7.81 g, 22 mmol) in DMF (37 mL), POCl$_3$ (3 mL) was added under stirring. The reaction mixture was stirred at rt for 15 min and then at 80 °C for 3 h. After cooling to 30 °C, an additional amount of POCl$_3$ (2 mL) was added to the mixture, and it was stirred at 80 °C for additional 3 h. The mixture was cooled to rt overnight. The reaction mixture was poured under stirring into water (350 mL) and kept stirring at 50 °C. To this heated resulting suspension, a 40% aqueous KOH solution is cautiously added up to pH ~9. The resulting mixture was stirred at reflux for 1 h and then cooled rt. The precipitate was filtered and washed cold water. The isomers were separated on a silica gel column using dichloromethane-acetone (40:1) as eluent.

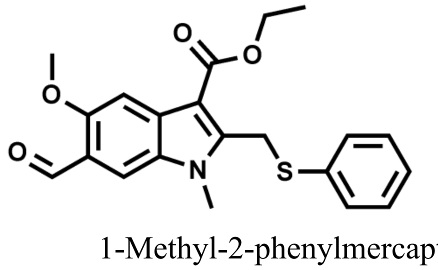

1-Methyl-2-phenylmercaptomethyl-3-carbethoxy-5-methoxy-6-formylindole: yield 2.77 g (33 %), mp 142-3 °C. HRMS calcd for $C_{21}H_{21}NO_4S$ ([M+H]$^+$) 384.1264; found 384.1267.

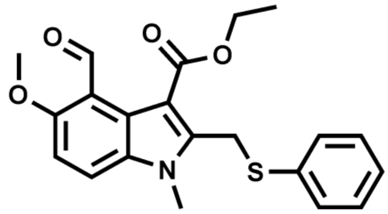

1-Methyl-2-phenylmercaptomethyl-3-carbethoxy-4-formyl-5-methoxyindole: yield 3.55 g (42 %), mp 148-9 °C. HRMS calcd for $C_{21}H_{21}NO_4S$ ($[M+H]^+$) 384.1264; found 384.1261.

Synthesis of 1-methyl-2-phenylmercaptomethyl-3-carbethoxy-5-methoxy-6-methylaminomethylindole hydrochloride (compound 4)

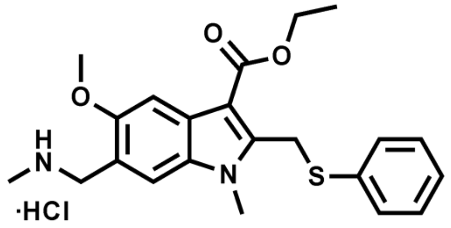

To a suspension of 1-methyl-2-phenylmercaptomethyl-3-carbethoxy-5-methoxy-6-formylindole (383 mg, 1 mmol) in ethanol (10 mL), a 33% methylamine solution in ethanol (2 mL) was added under stirring. After stirring at rt for 30 min, sodium borohydride (38 mg, 1 mmol) was added to the suspension, and the reaction mixture was stirred at 50 °C for 5 min, cooled to rt, and stirred for an additional 10 min. The mixture was evaporated in vacuo, and the residue was dissolved in ethyl acetate (30 mL), washed with water (2x10 mL) and dried over Na_2SO_4. The organic phase was evaporated in vacuo, and the residue was dissolved in acetone (5 mL) and acidified with a hydrogen chloride/ethanol solution to adjust pH ~3. The solvent was then removed in vacuo, and the residue was treated with acetone-ether mixture (1:1; 6 mL) and then stirred at 5 °C for 30 min. The precipitate was filtered, washed with cold acetone-ether mixture (1:1), ether, and dried. Yield 310 mg (71 %) of **4**, mp 179-182 °C. ^{1}H NMR (200 MHz; DMSO-d_6; δ, ppm): 1.30 (t, J = 7.1 Hz, 3H, $OCH_2C\underline{H}_3$), 3.72 (s, 3H, NCH_3), 3.87 (s, 3H, OCH_3), 4.10-4.28 (m, 4H, $OC\underline{H}_2CH_3$, $NH_2C\underline{H}_2$), 4.83 (s, 2H, SCH_2), 7.16-7.43 (m, 5H, Ph), 7.54 (s, 1H, HC(4)), 7.77 (d, J = 3.0 Hz, 1H, HC(7)), 9.20 (brs, 2H, NH_2). ^{13}C NMR (50 MHz; DMSO-d_6; δ, ppm): 14.00 ($OCH_2\underline{C}H_3$), 28.16 (SCH_2), 30.01 (NCH_3), 31.74 ($NH_2\underline{C}H_3$), 46.95 (NCH_2), 55.35 (OCH_3), 59.02 ($O\underline{C}H_2CH_3$), 101.25 (C(4)), 103.58 (C(3)), 113.51 (C(7)), 115.74 (C(6)), 126.77 (C(3a)), 127.10 (C(41)), 128.79 (C(3',5')), 130.62 (C(7a)), 130.95 (C(2',6')), 133.96 (C(1')), 143.28 (C(2)), 153.18 (C(5)), 164.03 (C=O). HRMS (ESI): calcd for $C_{22}H_{26}N_2O_3S$ ($[M+H]^+$) 399.1737; found 398.1739.

Synthesis of 1-methyl-2-phenylmercaptomethyl-3-carbethoxy-5-methoxy-6-hydroxymethylindole (compound 5)

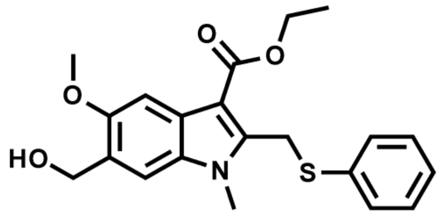

A suspension of 1-methyl-2-phenylmercaptomethyl-3-carbethoxy-5-methoxy-6-formylindole (383 mg, 1 mmol) and sodium borohydride (38 mg, 1 mmol) in ethanol (5 mL) was stirred at reflux for 2 min, then cooled and stirred at rt for 20 min and at 5 °C for 30 min. The precipitate was filtered, washed with ice-cold ethanol, 50% aqueous ethanol, water, and recrystallized from ethanol. Yield 315 mg (82 %) of **5**, mp 89-90 °C. ^{1}H NMR (200 MHz; DMSO-d_6; δ, ppm): 1.30 (t, *J* = 7.0 Hz, 3H, $OCH_2C\underline{H}_3$), 3.71 (s, 3H, NCH_3), 3.80 (s, 3H, OCH_3), 4.20 (q, *J* = 7.0 Hz, 2H, $OC\underline{H}_2CH_3$), 4.58 (s, 2H, $HOC\underline{H}_2$), 4.83 (s, 2H, SCH_2), 7.22-7.43 (m, 5H, Ph), 7.45 (s, 1H, HC(4)), 7.49 (s, 1H, HC(7)). ^{13}C NMR (50 MHz; DMSO-d_6; δ, ppm): 14.18 ($OCH_2\underline{C}H_3$), 28.28 (SCH_2), 30.01 (NCH_3), 55.12 (OCH_3), 58.33 ($HOCH_2$), 58.99 ($O\underline{C}H_2CH_3$), 100.83 (C(4)), 103.56 (C(3)), 108.42 (C(7)), 124.64 (C(6)), 127.09 (C(4)), 127.32 (C(3a)), 128.91 (C(3',5')), 130.89 (C(2',6')), 131.22 (C(7a)), 134.37 (C(1')), 141.65 (C(2)), 152.48 (C(5)), 164.43 (C=O). HRMS (ESI): calcd for $C_{21}H_{23}NO_4S$ ($[M+H]^+$) 386.1421; found 386.1420.

General procedure for the synthesis of 6-substituted 1-methyl-2-phenylmercaptomethyl-3-carbethoxy-5-methoxyindoles

To a solution of **4** (135 mg, 0.31 mmol) in dichloromethane (2mL), triethylamine (94 mg) was added under stirring. The mixture was cooled with ice water, and the corresponding acyl chloride (0.39 mmol) was added. The reaction mixture was stirred at rt for 30 min, and the mixture was then evaporated in vacuo. The residue was treated with 50% aqueous ethanol (2 mL), and the precipitate was filtered, washed with water, and recrystallized from the corresponding solvents.

1-Methyl-2-phenylmercaptomethyl-3-carbethoxy-5-methoxy-6-((benzoyl-(methyl)amino)methyl)indole compound 5 *E*/*Z* amide bond isomers (compound 7)

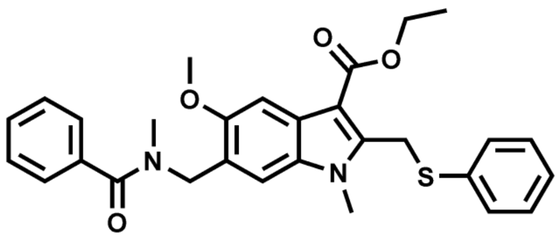

Yield 128 mg (82 %), 127-9 °C (ethanol). ^{1}H NMR (200 MHz; DMSO-d_6; δ, ppm): 1.30 (t, *J* = 6.9 Hz, 3H, $OCH_2C\underline{H}_3$), 2.90 (brs, 3H, $CONCH_3$), 3.75 (s, 3H, NCH_3), 3.72 and 3.85 (s, 3H, OCH_3), 4.20 (q, *J* = 7.0 Hz, 2H, $OC\underline{H}_2CH_3$),), 4.52 and 4.75 (s, 2H, NCH_2), 4.84 (s, 2H, SCH_2), 7.14-7.83 (m, 11H, 2Ph, HC(4)), 7.94 and 8.15 (d, *J* = 7.4 Hz, 1H, HC(7)). ^{13}C NMR (50 MHz; DMSO-d_6; δ, ppm): 14.00 ($OCH_2\underline{C}H_3$), 28.01 and 29.98 (SCH_2), 32.53 and 32.63 ($CON\underline{C}H_3$), 45.84 and 50.69 (NCH_2), 54.94 and 55.20 (OCH_3), 58.93 ($O\underline{C}H_2CH_3$), 101.33 (C(4)), 103.45 (C(3)), 109.73 (C(7)), 121.14 (C(6)), 125.31 (C(4’’)), 126.56 and 126.86 (C(3a)), 128.04 (C(2’’,6’’)), 128.78 (C(3’’,5’’)), 129.13 (C(3’,5’)), 130.16 (C(4’)), 130.47 and 131.00 (C(2’,6’)), 134.26 (C(7a)), 134.95 (C(1’)), 136.53 (C(1’’)), 142.14 (C(2)), 153.45 (C(5)), 164.14 (C=O), 170.73 and 171.77 (NC=O). HRMS (ESI): calcd for $C_{29}H_{30}N_2O_4S$ ($[M+H]^+$) 503.1999; found 503.2002.

1-Methyl-2-phenylmercaptomethyl-3-carbethoxy-5-methoxy-6-((ethoxycarbo-nyl(methyl)amino)methyl)indole (compound 6)

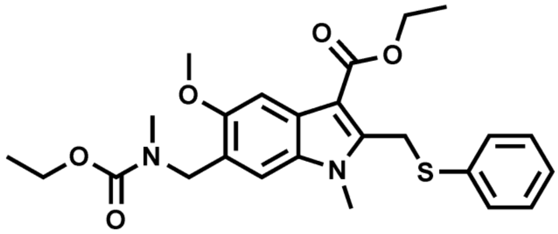

Yield 121 mg (83 %), 85-7 °C (toluene:heptane 1:1). ^{1}H NMR (200 MHz; DMSO-d_6; δ, ppm): 1.19 (brt, *J* = 7.1 Hz, 3H, $C\underline{H}_3CH_2OCON$), 1.30 (t, *J* = 7.1 Hz, 3H, $OCH_2C\underline{H}_3$), 2.85 (s, 3H, $CONCH_3$), 3.70 (s, 3H, NCH_3), 3.82 (s, 3H, OCH_3), 4.07 (q, *J* = 7.1 Hz, 2H, $CH_3C\underline{H}_2OCON$), 4.20 (q, *J* = 7.1 Hz, 2H, $OC\underline{H}_2CH_3$), 4.49 (s, 2H, NCH_2), 4.82 (s, 2H, SCH_2), 7.19 (s, 1H, HC(4)), 7.23-7.42 (m, 5H, Ph), 7.51 (s, 1H, HC(7)). ^{13}C NMR (50 MHz; DMSO-d_6; δ, ppm): 14.15 ($OCH_2\underline{C}H_3$), 14.64 ($\underline{C}H_3CH_2OCON$), 28.19 (SCH_2), 30.04 (NCH_3), 33.89 and 34.43 ($CON\underline{C}H_3$), 47.54 (NCH_2), 55.30 (OCH_3), 59.06 ($O\underline{C}H_2CH_3$), 60.61 ($CH_3\underline{C}H_2OCON$), 101.44 (C(4)), 103.57 (C(3)), 109.63 (C(7)), 122.08 (C(6)), 125.34 (C(3a)), 127.04 (C(4’)), 128.92 (C(3’,5’)), 130.76 (C(2’,6’)), 131.09 (C(7a)), 134.40 (C(1’)), 142.20 (C(2)), 153.28 (C(5)), 155.84 (O(CO)N), 164.33 (C=O). HRMS (ESI): calcd for $C_{25}H_{30}N_2O_5S$ ($[M+H]^+$) 471.1948; found 471.1945.

Synthesis of 1-methyl-2-phenylmercaptomethyl-3-carbethoxy-5-methoxy-6-methylaminomethylindole (compound 1)

To a suspension of 1-methyl-2-phenylmercaptomethyl-3-carbethoxy-4-formyl-5-methoxyindole (383 mg, 1 mmol) in 10 mL of ethanol, a 33% methylamine solution in ethanol (2 mL) was added under stirring. The suspension was stirred at rt for 30 min, and sodium borohydride (38 mg, 1 mmol) was added to the mixture and then stirred at rt for 45 min. The mixture was evaporated in vacuo, and the precipitate was filtered, washed with ethanol and water, and recrystallized from an acetone-ethanol (1:1) mixture.

The filtrate collected before the washing step with water was evaporated in vacuo, and to the residue, water (10 mL), acetic acid to pH 4, and activated carbon were added. The mixture was stirred at rt for 10 min and then filtered. A 10% aqueous KOH solution was added to the obtained solution up to pH 12, and the product's base was extracted with ethyl acetate (25 mL). The organic phase was washed with water (3x10 mL), dried over sodium sulfate, and evaporated in vacuo. The residue was dissolved in acetone (1 mL) and acidified with a hydrogen chloride/ethanol solution to adjust pH ~3. The solvent was then removed in vacuo, and the residue was dissolved in an acetone-ether (1:1) mixture and stirred at 5 °C for 30 min. The precipitate was filtered and washed with an acetone-ether (1:1) mixture and ether.

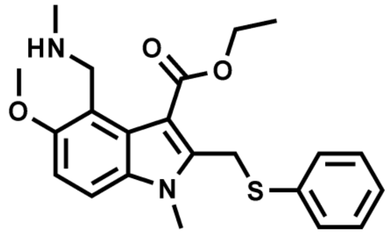

Yield 95 mg (22 %) of **3**, mp 172-184 °C. ^{1}H NMR (200 MHz; DMSO-d$_6$; δ, ppm): 1.25 (t, *J* = 7.1 Hz, 3H, $OCH_2C\underline{H}_3$), 3.34 (s, 3H, $NH_2C\underline{H}_3$), 3.71 (s, 3H, NCH_3), 3.89 (s, 3H, OCH_3), 4.20 (q, *J* = 7.1 Hz, 2H, $OC\underline{H}_2CH_3$), 4.61 (brs, 2H, NCH_2), 4.76 (s, 2H, SCH_2), 7.18 (d, *J* = 9.1 Hz, 1H, HC(6)), 7.26-7.42 (m, 5H, Ph), 7.67 (d, *J* = 9.1 Hz, 1H, HC(7)), 8.69 (brs, 2H, NH_2). ^{13}C NMR (50 MHz; DMSO-d$_6$; δ, ppm): 13.91 ($OCH_2\underline{C}H_3$), 29.56 (SCH_2), 30.25 (NCH_3), 32.03 (CH_3NH_2), 43.27 (NCH_2), 56.73 (OCH_3), 60.11 ($O\underline{C}H_2CH_3$), 104.70 (C(3)), 108.45 (C(7)), 109.87 (C(4)), 112.81 (C(6)), 125.68 (C(3a)), 127.34 (C(4')), 129.10 (C(3',5')), 131.01 (C(2',6')), 132.47 (C(7a)), 134.50 (C(1')), 143.94 (C(2)), 154.47 (C(5)), 165.03 (C=O). HRMS (ESI): calcd for $C_{22}H_{26}N_2O_3S$ ($[M+H]^+$) 399.1737; found 399.1735.

Synthesis of 1-methyl-2-phenylmercaptomethyl-3-carbethoxy-4-dimethylaminomethyl-5-methoxy-6-dimethylaminomethylindole dihydrochloride (compound 2)

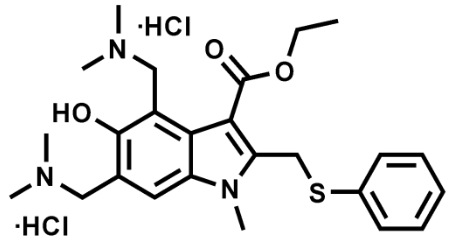

A mixture of ethyl 1-methyl-2-phenylmercaptomethyl-3-carbethoxy-5-hydroxyindole (171 mg, 0.5 mmol), a 40% dimethylamine aqueous solution (0.44 mL), and a 38% formaldehyde aqueous solution (182 mg) in acetic acid (1.7 mL) was stirred at 70 °C for 7 h. To the cooled reaction mixture, an additional 38% formaldehyde aqueous solution (95 mg) was added, and the mixture was then heated stirred at the same temperature overnight. Next day, the mixture was cooled to rt and and poured into ice-cold water (10 mL). To the cooled solution, 25% aqueous ammonia (3.5 mL) was added dropwise, and the resulting oil was extracted with ethyl acetate (30 mL). The organic layer was washed with water (3x10 mL), dried over Na_2SO_4, and evaporated in vacuo. The residue was dissolved in ethanol (3 mL) and acidified with a hydrogen chloride/ethanol solution to adjust pH ~3. The solvent was evaporated in vacuo, and the residue was treated with acetone. The precipitate was filtered, washed with an acetone-ether (1:1) mixture, and ether, and dried. Yield 170 mg (64 %) of **2**, mp 200 °C (decomp.). ^{1}H NMR (200 MHz; DMSO-d$_6$; δ, ppm): 1.25 (t, J = 7.1 Hz, 3H, OCH$_2$C$\underline{H}_3$), 2.76 (s, 12H, 2N(CH$_3$)$_2$), 3.74 (s, 3H, NOCH$_3$), 4.20 (q, J = 7.0 Hz, 2H, OC$\underline{H}_2$CH$_3$), 4.52 (s, 2H, NCH$_2$), 4.78 (s, 2H, SCH$_2$), 4.93 (s, 2H, NCH$_2$), 7.17-7.52 (m, 5H, Ph), 8.06 (s, 1H, HC(7)), 9.09 (brs, 1H, NH), 9.59 (s, 1H, OH), 10.78 (brs, 1H, NH). ^{13}C NMR (50 MHz; DMSO-d$_6$; δ, ppm): 13.85 (OCH$_2$$\underline{C}H_3$), 29.65 (SCH$_2$), 30.40 (NCH$_3$), 41.54 (N(CH$_3$)$_2$), 42.14 (N(CH$_3$)$_2$), 52.65 (NCH$_2$), 55.53 (NCH$_2$), 60.33 (O$\underline{C}H_2CH_3$), 105.12 (C(3)), 111.30 (C(7)), 116.70 (C(4)), 117.21 (C(6)), 126.79 (C(3a)), 127.40 (C(4’)), 129.13 (C(3’,5’)), 131.04 (C(2’,6’)), 131.83 (C(7a)), 134.41 (C(1’)), 144.60 (C(2)), 151.36 (C(5)), 165.09 (C=O). HRMS (ESI): calcd for $C_{25}H_{33}N_3O_3S$ ([M+H]$^+$) 456.2315; found 456.2317.

Synthesis and characterization of compounds with R4 and R5 modifications

All procedures for synthesizing and characterizing compounds with modifications to the R4 and R5 groups, including their isomers, were performed at the Holistic Drug Discovery and Development Center, University of Cape Town, South Africa.

General procedures

The first step towards accessing the targeted **UMF** analogs was the synthesis of β-enamino esters that bore the chosen *N*-alkyl substituents, i.e., methyl, cyclopropyl, and cyclopropyl methane. These β-enamino ester indole precursors were prepared via aqueous enamine synthesis using ethyl acetoacetate and the appropriate primary amine **(Scheme 2)**.[3] β-Enamino esters were then condensed with 1,4-benzoquinone in dichloroethane using Nenitzescu indolisation conditions to afford the desired 5-hydroxyindoles.[4,5] 5-hydroxyindoles were methylated using iodomethane under basic conditions to afford 5-methoxyindoles, and radical chlorination with *N*-chlorosuccinimide (NCS) using 1,1'-azobis(cyclohexanecarbonitrile) (ABCN) as an initiator afforded the key chloromethyl indole intermediates in excellent yields (85 – 95%). Thioether intermediates were obtained by adding the desired chloromethyl indole to a solution of the appropriate *in situ*-generated potassium thiophenolate. The synthesis of 6-(dimethylamino)methyl-5-methoxyindoles **8, 11–14, 16** and **18–27** was achieved using Mannich reaction conditions by heating the appropriate thioethers in acetic acid at 140 °C with bis(dimethylamino)methane as an amine source. The addition of the Mannich moiety occurred primarily at position 6 of indoles **8, 11–14, 16** and **18–27** under these conditions. Although a trace amount of the 4-substituted regioisomer formed as a minor product, tedious high-performance liquid chromatography (HPLC) allowed for the separation of the two isomers. Regrettably, this method produced final compounds in meager yields (1 – 6%), which meant that reactions had to be performed on a large scale (500 mg – 1 gram) to generate sufficient material for biological evaluation.

An alternative, higher-yielding method that did not require purification via HPLC, i.e., formylation and reductive amination, was later found to be a more economical way to synthesize the remaining target molecules. This strategy allowed for the synthesis of both 4-substituted and 6-substituted **UMF** analogs due to the formation of two formyl regioisomers that were easily separable via column chromatography **(Scheme 2)**. 6- and

4-formyl indole intermediates were synthesized using a Vilsmeier-Haack formylation reaction by heating under reflux with phosphorus oxychloride ($POCl_3$) and dimethylformamide (DMF). The remaining 6-(dimethylamino)methyl-5-methoxyindoles **9, 10, 15** and **17** were synthesized via reductive amination with dimethylamine and sodium triacetoxyborohydride ($NaBH(OAc)_3$) in dichloromethane (DCM).

Scheme S2

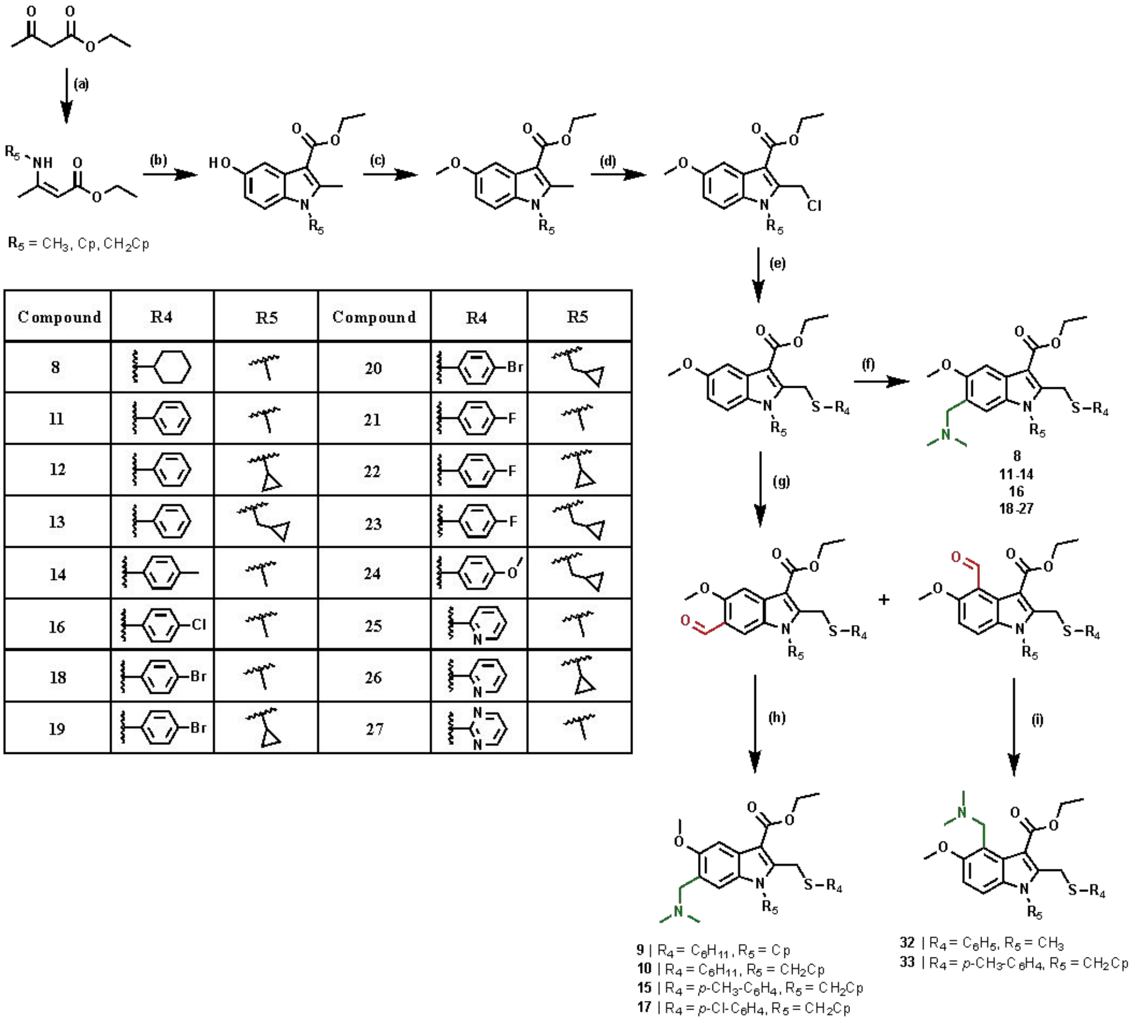

Reagents and conditions: (a) R_5NH_2, H_2O, rt, 16 h; **(b)** 1,4-benzoquinone, DCE, 90 °C, 16 h; **(c)** MeI, K_2CO_3, DMF, rt, 16 h; **(d)** NCS, ABCN, $CHCl_3$, 90 °C, 1.5 h; **(e)** 1) R_4SH, KOH, MeOH, rt, 30 min, 2) chloroindole, rt, 4 h; **(f)** $Me_2NCH_2NMe_2$, AcOH, 140 °C, 2 h; **(g)** 1) $POCl_3$, DMF, rt, 40 min, 2)100 °C, 1.25 h; **(h)** $Me_2NH{\cdot}HCl$, Et_3N, $NaBH(OAc)_3$, DCM, rt, 2 h; **(i)** $Me_2NH{\cdot}HCl$, Et_3N, $NaBH(OAc)_3$, DCM, rt, 2 h.

Shifting the focus to the 4-substituted **UMF** analogs, the appropriate 4-formyl indole intermediates were subsequently converted to 4-(dimethylamino)methyl indoles

32 and **33**, 4-substituted regioisomers of indoles **11** and **15**, via reductive amination using dimethylamine hydrochloride, Et_3N and $NaBH(OAc)_3$ in DCM.

Four additional **UMF** analogs were synthesized by extending the thiophenol fragment of the methyl and cyclopropyl methane aryl bromide intermediates **(Scheme 3)**. This was achieved by subjecting each aryl bromide to a Suzuki-Miyaura cross-coupling reaction with two different boronic acids, namely (6-(trifluoromethyl)pyridin-3-yl)boronic acid and (4-fluorophenyl)boronic acid. This yielded biaryl indole intermediates which were formylated using the previously described Vilsmeier-Haack reaction conditions to furnish 6-formyl biaryl indoles. Finally, reductive amination with dimethylamine hydrochloride as described previously in **Scheme 2** afforded 6-(dimethylamino)methyl-5-methoxyindoles **28 – 31** with extended biaryl moieties.

Scheme S3

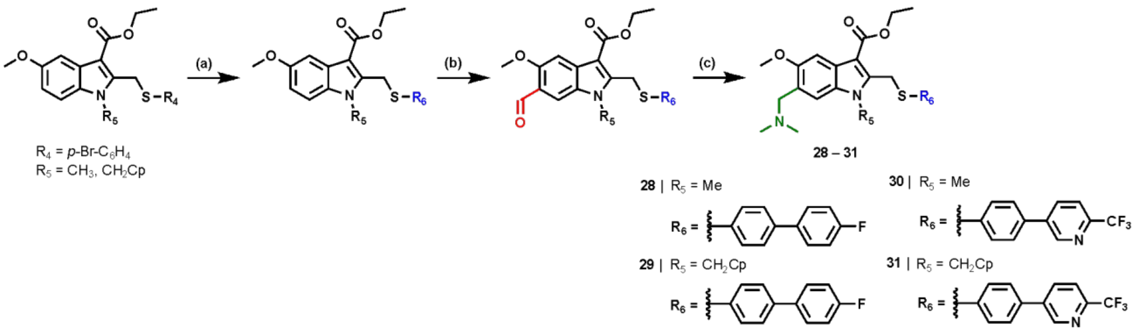

Reagents and conditions: (a) R_2-$B(OH)_2$, $Pd(dppf)Cl_2 \cdot DCM$, K_3PO_4, 1,4-dioxane:H_2O (5:1), 100 °C, MW, 1 h; **(b)** $POCl_3$, DMF, reflux, 6 h; **(c)** $Me_2NH \cdot HCl$, Et_3N, $NaBH(OAc)_3$, DCM, rt, 2 h.

Synthesis of Enaminones

The appropriate amine (76.8 mmol, 2.0 equiv)[6] was added to a stirring solution of ethyl acetoacetate (4.86mL, 38.4 mmol) in water (192 mL) in a 250 mL round-bottom flask. The mixture turned milky almost immediately. The reaction mixture was stirred at room temperature for 15 hours, after which brine (150 mL) was added to the clear solution. The product was extracted with DCM (3 x 100 mL), and the combined organic extracts were dried over $MgSO_4$ before being concentrated under reduced pressure to afford the desired enaminone (82 – 97%) used in the next step.

The product's LC-MS analysis did not produce the desired mass, so 1H NMR spectroscopic analysis was used to confirm its formation.

Ethyl (Z)-3-(methylamino)but-2-enoate, yellowish oil, 90% yield.

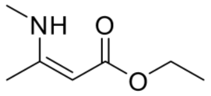

* A solution of 2.0 M methylamine in THF (39.00 mL, 76.84 mmol, 2.0 equiv) in 154 mL H_2O.

^{1}H NMR (300 MHz, $CDCl_3$) δ 8.46 (br. s, 1H), 4.44 (s, 1H), 4.05 (q, 2H, *J* = 7.1 Hz), 2.88 (d, 3H, *J* = 7.1 Hz), 1.89 (s, 3H), 1.22 (t, 3H, *J* = 7.1 Hz).

Ethyl (Z)-3-(cyclopropylamino)but-2-enoate, yellowish oil, 97% yield.

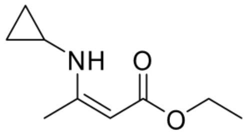

^{1}H NMR (300 MHz, $CDCl_3$) δ 8.51 (br. s, 1H), 4.50 (s, 1H), 4.04 (q, 2H, *J* = 7.1 Hz), 2.58 – 2.50 (m, 1H), 2.03 (s, 3H), 1.21 (t, 3H, *J* = 7.1 Hz), 0.75 – 0.69 (m, 2H), 0.58 – 0.53 (m, 2H).

Ethyl (Z)-3-((cyclopropylmethyl)amino)but-2-enoate, orange oil, 82% yield.

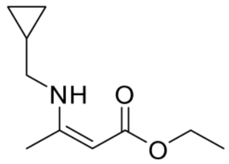

^{1}H NMR (300 MHz, $CDCl_3$) δ 8.61 (br. s, 1H), 4.42 (s, 1H), 4.07 (q, 2H, *J* = 7.1 Hz), 3.06 (t, 2H, *J* = 6.3 Hz), 1.89 (s, 3H), 1.24 (t, 3H, *J* = 7.1 Hz), 1.04 – 0.91 (m, 1H), 0.57 – 0.51 (m, 2H), 0.23 – 0.18 (m, 2H).

Nenitzescu Indolisation

The appropriate enaminone (1.1 equiv) was added to a solution of *p*-benzoquinone (1.0 equiv) in DCE (c = 0.83 M) in a 100 mL round-bottom flask. The reaction mixture was heated under reflux at 90 °C for 16 h under nitrogen before being cooled to room temperature. The solvent was removed under reduced pressure, and the resultant residue was diluted with cold DCM. The precipitate that formed was filtered and dried to afford the desired indole (35 – 51%), which was used as is in the next step.

Ethyl 5-hydroxy-1,2-dimethyl-1*H*-indole-3-carboxylate, dark-brown solid, 40% yield.

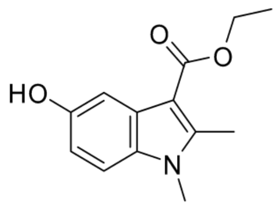

LC-MS ESI$^+$: t_R = 0.857 min (purity 88%); exact mass calcd for $C_{13}H_{15}NO_3$: 233.11, found: *m/z* 234.2 [M + H]$^+$.

Ethyl 1-cyclopropyl-5-hydroxy-2-methyl-1*H*-indole-3-carboxylate, light-brown powder, 51% yield.

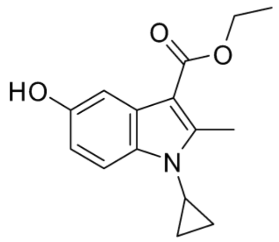

LC-MS ESI$^+$: t_R = 1.018 min (purity 94%); exact mass calcd for $C_{15}H_{17}NO_3$: 259.12, found: *m/z* 260.2 [M + H]$^+$.

Ethyl 1-(cyclopropylmethyl)-5-hydroxy-2-methyl-1*H*-indole-3-carboxylate, dark-brown solid, 35% yield.

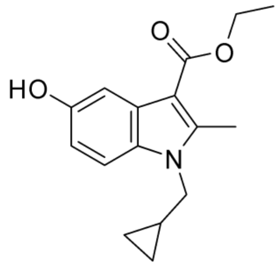

LC-MS ESI$^+$: t_R = 1.050 min (purity 92%); exact mass calcd for $C_{16}H_{19}NO_3$: 273.14, found: *m/z* 274.2 [M + H]$^+$.

Methylation Procedure

K_2CO_3 (2.3 equiv) and iodomethane (2.3 equiv) were added to a solution of the appropriate indole (1.0 equiv) in DMF (C = 0.4 M) in a sealable vial. The vial was sealed, and the reaction mixture was stirred at room temperature overnight. LC-MS analysis indicated reaction completion, so the reaction mixture was diluted with EtOAc and H_2O. The layers were separated, and the aqueous phase was extracted with EtOAc (3X). The combined organic extracts were washed with H_2O (5X) and dried over $MgSO_4$ before being concentrated under reduced pressure. The crude residue was purified via silica gel flash column chromatography using a Teledyne ISCO CombiFlash system eluting a

gradient of EtOAc in hexane (0 – 30%) to afford the desired methylated indole (82 – 93%), which was used as is in the next step.

Ethyl 5-methoxy-1,2-dimethyl-1*H*-indole-3-carboxylate, cream-coloured crystals, 85% yield

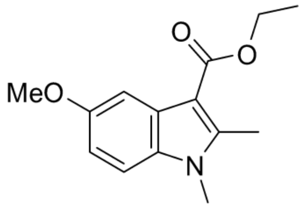

LC-MS ESI$^+$: t_R = 1.166 min (purity 96%); exact mass calcd for $C_{14}H_{17}NO_3$: 247.12, found: *m/z* 248.2 $[M + H]^+$.

Ethyl 1-cyclopropyl-5-methoxy-2-methyl-1*H*-indole-3-carboxylate, cream-coloured crystals, 93% yield.

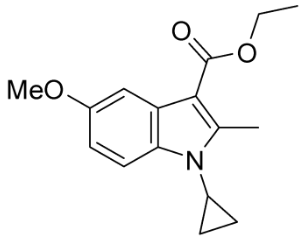

LC-MS ESI$^+$: t_R = 1.133 min (purity 100%); exact mass calcd for $C_{16}H_{19}NO_3$: 273.14, found: *m/z* 274.2 $[M + H]^+$.

Ethyl 1-(cyclopropylmethyl)-5-methoxy-2-methyl-1*H*-indole-3-carboxylate, peach-coloured crystals, 82% yield.

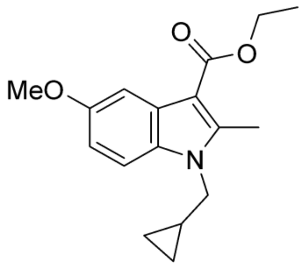

LC-MS ESI$^+$: t_R = 1.219 min (purity 99%); exact mass calcd for $C_{17}H_{21}NO_3$: 287.15, found: *m/z* 288.2 $[M + H]^+$.

Radical Chlorination Reaction

N-Chlorosuccinimide (1.1 equiv) was added to a solution of the appropriate methylated indole (1.0 eq.) and 1-[(*E*)-(1-cyanocyclohexyl)diazenyl]cyclohexane-1-carbonitrile (0.10 equiv) in $CHCl_3$ (C = 0.43 M) at room temperature. The reaction mixture was then heated at 80 °C for 1 h before cooling to room temperature. The solvent

was removed under reduced pressure, and the residue was redissolved in DCM. The organic phase was washed with H_2O and dried over Na_2SO_4 before being concentrated under reduced pressure to afford the desired chlorinated indole which was of sufficient purity for the subsequent step.

Ethyl 2-(chloromethyl)-5-methoxy-1-methyl-1*H*-indole-3-carboxylate, greenish-yellow crystals, 92% yield.

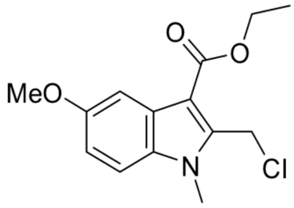

LC-MS ESI$^+$: t_R = 0.889 min (purity 85%); exact mass calcd for $C_{14}H_{16}ClNO_3$: 281.08, found: *m/z* 246.1 [M – Cl]$^+$.

^{1}H NMR (300 MHz, CDCl$_3$) δ 7.67 (d, 1H, *J* = 2.1 Hz), 7.25 (d, 1H, *J* = 9.1 Hz), 6.96 (dd, 1H, *J* = 9.1, 2.1 Hz), 5.25 (s, 2H), 4.43 (q, 2H, *J* = 7.1 Hz), 3.89 (s, 3H), 3.80 (s, 3H), 1.47 (t, 3H, *J* = 7.1 Hz).

Ethyl 2-(chloromethyl)-1-cyclopropyl-5-methoxy-1*H*-indole-3-carboxylate, yellowish-orange crystals, 86% yield.

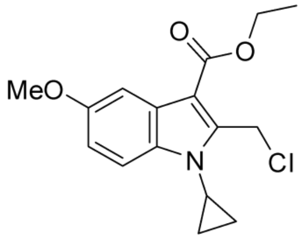

LC-MS ESI$^+$: t_R = 0.999 min (purity 80%); exact mass calcd for $C_{16}H_{18}ClNO_3$: 307.1, found: *m/z* 272.2 [M – Cl]$^+$.

^{1}H NMR (400 MHz, CDCl$_3$) δ 7.66 (d, 1H, *J* = 2.1 Hz), 7.51 (d, 1H, *J* = 9.1 Hz), 6.94 (dd, 1H, *J* = 9.1, 2.1 Hz), 5.35 (s, 2H), 4.43 (q, 2H, *J* = 7.1 Hz), 3.88 (s, 3H), 3.33 – 3.26 (m, 1H), 1.46 (t, 3H, *J* = 7.1 Hz), 1.30 – 1.24 (m, 2H), 1.23 – 1.18 (m, 2H).

* Residual NCS and other trace impurities observed in ^{1}H NMR spectrum.

Ethyl 2-(chloromethyl)-1-(cyclopropylmethyl)-5-methoxy-1*H*-indole-3-carboxylate, greenish-yellow crystals, 94% yield.

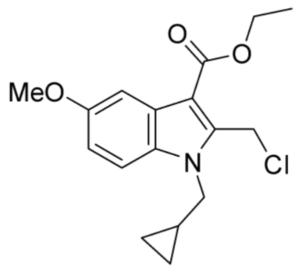

LC-MS ESI$^+$: t_R = 1.255 min (purity 92%); exact mass calcd for $C_{17}H_{20}ClNO_3$: 321.11, found: *m/z* 286.2 [M – Cl]$^+$.

^{1}H NMR (300 MHz, CDCl$_3$) δ 7.68 (d, 1H, *J* = 2.1 Hz), 7.29 (d, 1H, *J* = 9.1 Hz), 6.95 (dd, 1H, *J* = 9.1, 2.1 Hz), 5.27 (s, 2H), 4.43 (q, 2H, *J* = 7.1 Hz), 4.16 (d, 2H, *J* = 6.4 Hz), 3.89 (s, 3H), 3.80 (s, 3H), 1.47 (t, 3H, *J* = 7.1 Hz), 1.31 – 1.21 (m, 1H), 0.64 – 0.58 (m, 2H), 0.43 – 0.38 (m, 2H).

Synthesis of Substituted ((Phenylthio)methyl)indole Derivatives

The appropriate thiophenol (1.1 equiv) was added dropwise/portionwise to a stirring solution of KOH (1.0 equiv) in MeOH (*C* = 1 M) at room temperature. The reaction mixture was stirred at room temperature for 30 min, after which the appropriate chloromethyl indole (1.0 equiv) was added portion wise to the solution with rapid stirring. Stirring was continued for 2 – 4 h at room temperature (reaction monitored by LC-MS) before removal of the solvent under reduced pressure. The crude residue was then purified directly via silica gel flash column chromatography using a Teledyne ISCO CombiFlash system eluting a gradient of EtOAc in hexane (typically 0 – 60%) to afford the desired product (51 – 93%), which was used as is in the next step.

Ethyl 5-methoxy-1-methyl-2-((phenylthio)methyl)-1*H*-indole-3-carboxylate – light-yellow solid, 87% yield.

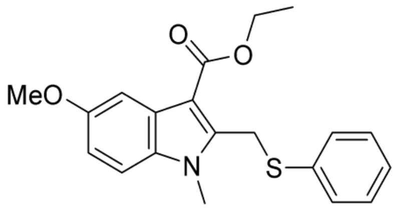

LC-MS ESI$^+$: t_R = 1.306 min (purity 97%); exact mass calcd for $C_{20}H_{21}NO_3S$: 355.12, found: *m/z* 356.1 [M + H]$^+$.

^{1}H NMR (300 MHz, $CDCl_3$) δ 7.66 (d, 1H, *J* = 2.3 Hz), 7.38 – 7.35 (m, 2H), 7.26 – 7.24 (m, 3H), 7.19 (d, 1H, *J* = 8.8 Hz), 6.92 (dd, 1H, *J* = 8.8, 2.3 Hz), 4.71 (s, 2H), 4.29 (q, 2H, *J* = 7.1 Hz), 3.89 (s, 3H), 2.63 (s, 3H), 1.38 (t, 3H, *J* = 7.1 Hz).

Ethyl 2-(((4-bromophenyl)thio)methyl)-5-methoxy-1-methyl-1*H*-indole-3-carboxylate – yellow solid, 84% yield.

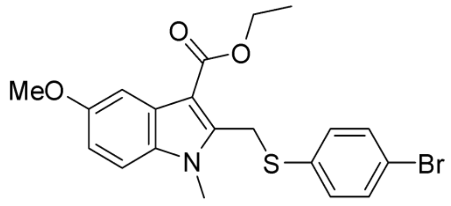

LC-MS ESI$^+$: t_R = 1.322 min (purity 99%); exact mass calcd for $C_{20}H_{20}BrN_2O_3S$: 433.03, found: *m/z* 434.0 $[M + H]^+$.

Ethyl 2-(((4-fluorophenyl)thio)methyl)-5-methoxy-1-methyl-1*H*-indole-3-carboxylate – light-yellow solid, 82% yield.

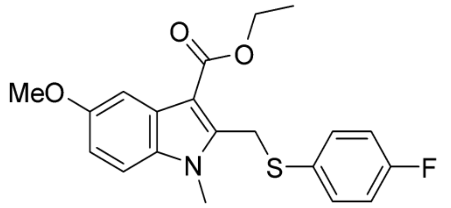

LC-MS ESI$^+$: t_R = 1.252 min (purity 98%); exact mass calcd for $C_{20}H_{20}FNO_3S$: 373.11, found: *m/z* 374.1 $[M + H]^+$.

Ethyl 2-(((4-chlorophenyl)thio)methyl)-5-methoxy-1-methyl-1*H*-indole-3-carboxylate – yellow solid, 74% yield.

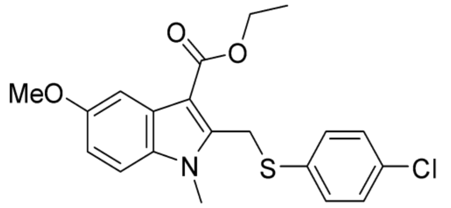

LC-MS ESI$^+$: t_R = 1.304 min (purity 98%); exact mass calcd for $C_{20}H_{20}ClNO_3S$: 389.09, found: *m/z* 390.1 $[M + H]^+$.

Ethyl 5-methoxy-1-methyl-2-((pyridin-2-ylthio)methyl)-1*H*-indole-3-carboxylate – yellow solid, 92% yield.

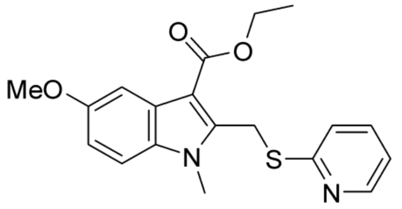

LC-MS ESI$^+$: t_R = 1.171 min (purity 97%); exact mass calcd for $C_{19}H_{20}N_2O_3S$: 356.12, found: *m/z* 357.1 [M + H]$^+$.

Ethyl 5-methoxy-1-methyl-2-((pyrimidin-2-ylthio)methyl)-1*H*-indole-3-carboxylate - yellow solid, 82% yield.

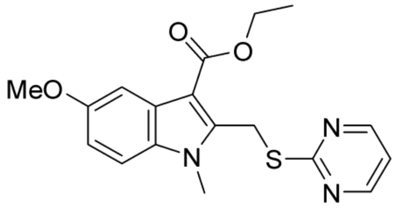

LC-MS ESI$^+$: t_R = 1.100 min (purity 92%); exact mass calcd for $C_{18}H_{19}N_3O_3S$: 357.11, found: *m/z* 358.1 [M + H]$^+$.

Ethyl 5-methoxy-1-methyl-2-((*p*-tolylthio)methyl)-1*H*-indole-3-carboxylate – light-yellow oil, 93% yield.

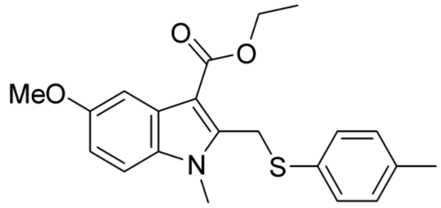

LC-MS ESI$^+$: t_R = 1.296 min (purity 98%); exact mass calcd for $C_{21}H_{23}NO_3S$: 369.14, found: *m/z* 370.1 [M+H]$^+$.

Ethyl 2-((cyclohexylthio)methyl)-5-methoxy-1-methyl-1*H*-indole-3-carboxylate – white solid, 89% yield.

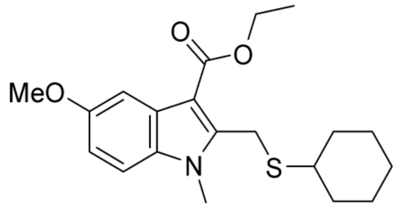

LC-MS ESI$^+$: t_R = 1.403 min (purity 100%);exact mass calcd for $C_{20}H_{27}NO_3S$: 361.17, found: *m/z* 362.2 [M + H]$^+$.

Ethyl 1-cyclopropyl-5-methoxy-2-((phenylthio)methyl)-1*H*-indole-3-carboxylate – yellow solid, 81% yield.

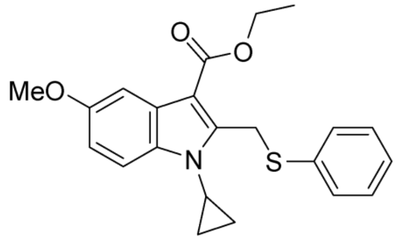

LC-MS ESI⁺: t_R = 1.308 min (purity 95%);exact mass calcd for $C_{22}H_{23}NO_3S$: 381.13, found: *m/z* 382.1 [M + H]⁺.

Ethyl 2-(((4-bromophenyl)thio)methyl)-1-cyclopropyl-5-methoxy-1*H*-indole-3-carboxylate – yellow gum, 59% yield.

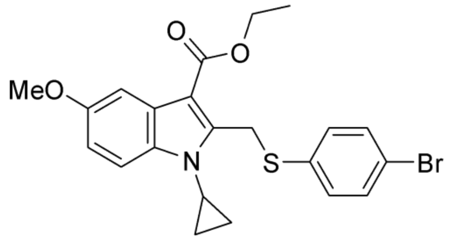

LC-MS ESI⁺: t_R = 1.367 min (purity 95%); exact mass calcd for $C_{22}H_{22}BrNO_3S$: 459.05, found: *m/z* 460.0 [M + H]⁺.

Ethyl 1-cyclopropyl-2-(((4-fluorophenyl)thio)methyl)-5-methoxy-1*H*-indole-3-carboxylate – yellow solid, 78% yield.

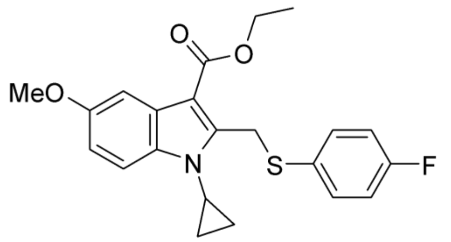

LC-MS ESI⁺: t_R = 1.306 min (purity 95%); exact mass calcd for $C_{22}H_{22}FNO_3S$: 399.13, found: *m/z* 400.1 [M + H]⁺.

Ethyl 1-cyclopropyl-5-methoxy-2-((pyridin-2-ylthio)methyl)-1*H*-indole-3-carboxylate – yellow solid, 72% yield.

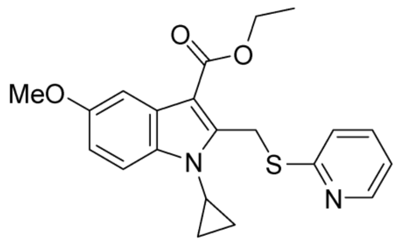

LC-MS ESI⁺: t_R = 1.221 min (purity 99%); exact mass calcd for $C_{21}H_{22}N_2O_3S$: 382.13, found: *m/z* 383.1 [M + H]⁺.

Ethyl 2-((cyclohexylthio)methyl)-1-cyclopropyl-5-methoxy-1*H*-indole-3-carboxylate – yellow oil, 38% yield.

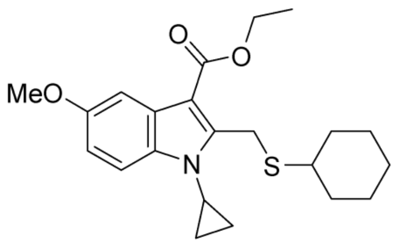

LC-MS ESI$^+$: t_R = 1.629 min (purity 90%); exact mass calcd for $C_{22}H_{29}NO_3S$: 387.19, found: *m/z* 388.2 $[M + H]^+$.

Ethyl 1-(cyclopropylmethyl)-5-methoxy-2-((phenylthio)methyl)-1H-indole-3-carboxylate – yellow solid, 93% yield.

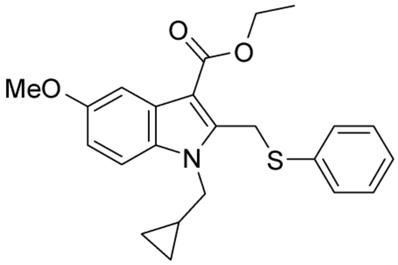

LC-MS ESI$^+$: t_R = 1.154 min (purity 98%);exact mass calcd for $C_{23}H_{25}NO_3S$: 395.15, found: *m/z* 396.2 $[M + H]^+$.

Ethyl 2-(((4-bromophenyl)thio)methyl)-1-(cyclopropylmethyl)-5-methoxy-1H-indole-3-carboxylate – yellow solid, 95% yield.

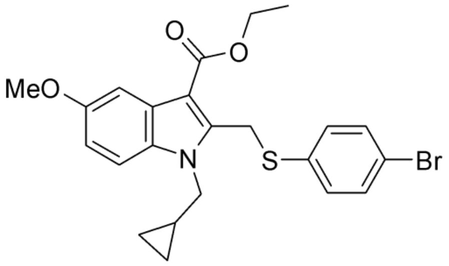

LC-MS ESI$^+$: t_R = 1.515 min (purity 94%); exact mass calcd for $C_{23}H_{24}BrNO_3S$: 473.07, found: *m/z* 474.1 $[M + H]^+$.

Ethyl 1-(cyclopropylmethyl)-2-(((4-fluorophenyl)thio)methyl)-5-methoxy-1H-indole-3-carboxylate – yellow solid, 82% yield.

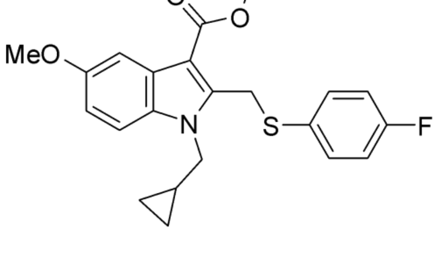

LC-MS ESI⁺: t_R = 1.337 min (purity 95%); exact mass calcd for $C_{23}H_{24}FNO_3S$: 413.14, found: *m/z* 414.1 [M + H]⁺.

Ethyl 1-(cyclopropylmethyl)-5-methoxy-2-(((4-methoxyphenyl)thio)methyl)-1*H*-indole-3-carboxylate – yellow solid, 91% yield.

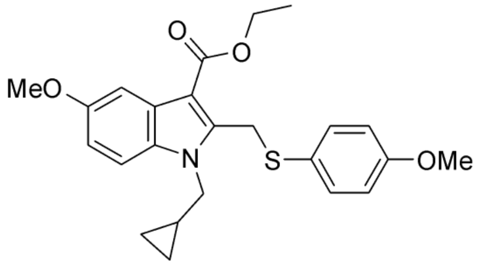

LC-MS ESI⁺: t_R = 1.411 min (purity 97%);exact mass calcd for $C_{24}H_{27}NO_4S$: 425.17, found: *m/z* 426.1 [M + H]⁺.

Ethyl 1-(cyclopropylmethyl)-5-methoxy-2-((*p*-tolylthio)methyl)-1H-indole-3-carboxylate – yellow oil, 88% yield.

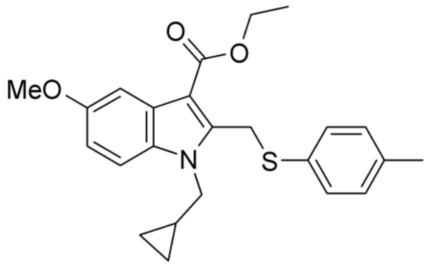

LC-MS ESI⁺: t_R = 1.369 min (purity 95%);exact mass calcd for $C_{24}H_{27}NO_3S$: 409.17, found: *m/z* 410.1 [M + H]⁺.

Ethyl 2-((cyclohexylthio)methyl)-1-(cyclopropylmethyl)-5-methoxy-1H-indole-3-carboxylate – pale-orange solid, 81% yield.

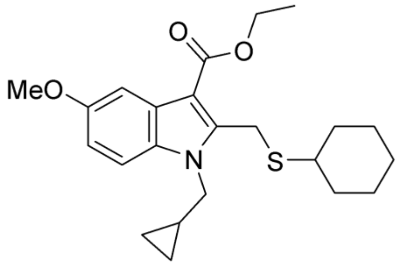

LC-MS ESI⁺: t_R = 1.216 min (purity 100%);exact mass calcd for $C_{23}H_{31}NO_3S$: 401.20, found: *m/z* 286.2 [M – SCy]⁺.

Ethyl 2-(((4-chlorophenyl)thio)methyl)-1-(cyclopropylmethyl)-5-methoxy-1H-indole-3-carboxylate – brown oil, 51%

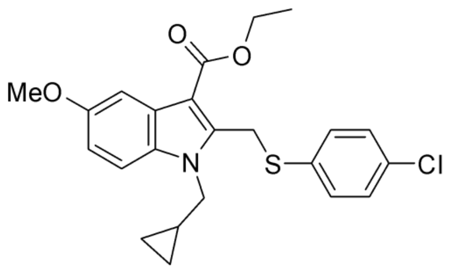

LC-MS ESI⁺: t_R = 1.414 min (purity 74%); exact mass calcd for $C_{23}H_{24}ClNO_3S$: 429.11, found: *m/z* 430.1 $[M + H]^+$.

Suzuki-Miyaura Coupling Reaction

The appropriate aryl bromide (methyl or cyclopropyl methane) (2.99 mmol), K_3PO_4 (1.27 g, 5.99 mmol, 2.0 equiv), 1,4-dioxane (10 mL) and H_2O (2 mL) were added to a large microwave tube. The reaction mixture was then degassed with nitrogen before the addition of the appropriate boronic acid (3.59 mmol, 1.2 equiv) and [1,1′-Bis(diphenylphosphino)ferrocene]dichloropalladium(II) dichloromethane complex (245 mg, 0.299 mmol, 0.10 equiv). After degassing once more, the reaction mixture was heated at 100 °C for 1 h in a microwave reactor. LC-MS analysis indicated reaction completion, so the reaction mixture was filtered through a plug of Celite before removal of the solvent under reduced pressure. The crude residue was purified via silica gel flash column chromatography using a Teledyne ISCO Combiflash system eluting a gradient of EtOAc in hexane (typically 0 – 90%) to afford the desired product (63 – 78%), which was used as is in the next step.

Ethyl 2-(((4'-fluoro-[1,1'-biphenyl]-4-yl)thio)methyl)-5-methoxy-1-methyl-1*H*-indole-3-carboxylate – yellow solid, 63% yield.

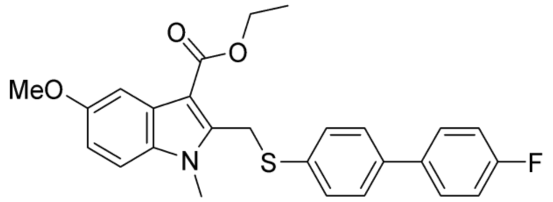

LC-MS ESI⁺: t_R = 1.421 min (purity 83%); exact mass calcd for $C_{26}H_{24}FNO_3S$: 449.15, found: *m/z* 450.1 $[M + H]^+$.

Ethyl 5-methoxy-1-methyl-2-(((4-(6-(trifluoromethyl)pyridin-3-yl)phenyl)thio)methyl)-1*H*-indole-3-carboxylate – orange solid, 79% yield.

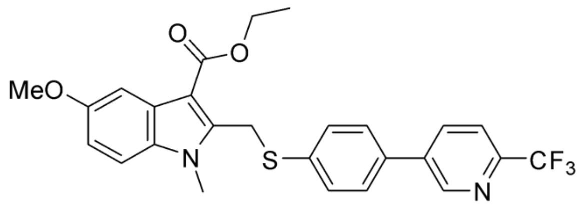

LC-MS ESI$^+$: t_R = 1.393 min (purity 90%); exact mass calcd for $C_{26}H_{23}F_3N_2O_3S$: 500.13, found: *m/z* 501.1 [M + H]$^+$.

Ethyl 1-(cyclopropylmethyl)-2-(((4'-fluoro-[1,1'-biphenyl]-4-yl)thio)methyl)-5-methoxy-1H-indole-3-carboxylate – brown oil, 84% yield.

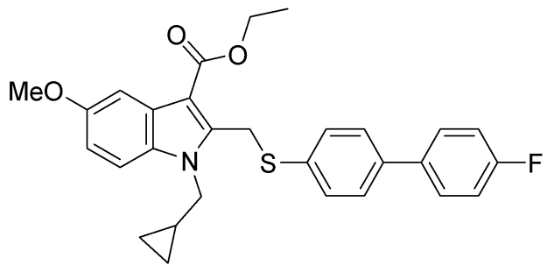

LC-MS ESI$^+$: t_R = 1.423 min (purity 94%); exact mass calcd for $C_{29}H_{28}FNO_3S$: 489.17, found: *m/z* 490.2 [M + H]$^+$.

Ethyl 1-(cyclopropylmethyl)-5-methoxy-2-(((4-(6-(trifluoromethyl)pyridin-3-yl)phenyl)thio)methyl)-1H-indole-3-carboxylate – brown solid, 61% yield.

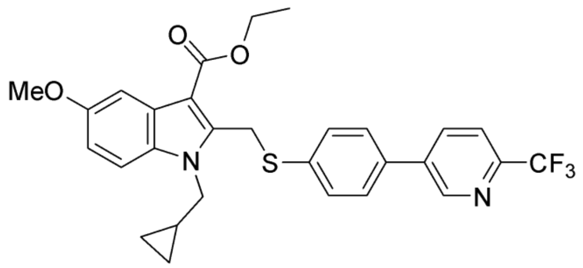

LC-MS ESI$^+$: t_R = 1.566 min (purity 95%); exact mass calcd for $C_{29}H_{27}F_3N_2O_3S$: 541.2, found: *m/z* 490.2 [M + H]$^+$.

4. References

(1) Alves, J.; Engel, L.; de Vasconcelos Cabral, R.; Rodrigues, E. L.; de Jesus Ribeiro, L.; Higa, L. M.; da Costa Ferreira Júnior, O.; Castiñeiras, T. M. P. P.; de Carvalho Leitão, I.; Tanuri, A.; Goueli, S. A.; Zegzouti, H. A Bioluminescent and Homogeneous SARS-CoV-2 Spike RBD and HACE2 Interaction Assay for Antiviral Screening and Monitoring Patient Neutralizing Antibody Levels. *Sci Rep* **2021**, *11* (1). https://doi.org/10.1038/s41598-021-97330-3.

(2) Trofimov, F. A.; Tsyshkova, N. G.; Grinev, A. N.; Shadurskii, K. S. Studies in the Quinone Field XLVI. Synthesis of Thioethers and Sulfones in the 5-Hydroxyindole Series. *Pharm Chem J* **1969**, *3* (11). https://doi.org/10.1007/BF00759819.

(3) Stefani, H. A.; Costa, I. M.; Silva, O. An Easy Synthesis of Enaminones in Water as Solvent. **2000**.

(4) Monti, S. A. *The Nentizescu Condensation of Ethyl 3-Aminocrotonate and 1,4-Benzoquinone1*; 1962; Vol. 37.

(5) Chai, H.; Zhao, Y.; Zhao, C.; Gong, P. Synthesis and in Vitro Anti-Hepatitis B Virus Activities of Some Ethyl 6-Bromo-5-Hydroxy-1H-Indole-3-Carboxylates. *Bioorg Med Chem* **2006**, *14* (4), 911–917. https://doi.org/10.1016/j.bmc.2005.08.041.

(6) Stefani, H. A.; Costa, I. M.; De Silva, D. O. An Easy Synthesis of Enaminones in Water as Solvent. *Synthesis (Stuttg)* **2000**, No. 11. https://doi.org/10.1055/s-2000-7608.
